# DNA-barcoded labeling probes for highly multiplexed Exchange-PAINT imaging†
†Electronic supplementary information (ESI) available. See DOI: 10.1039/c6sc05420j



**DOI:** 10.1039/c6sc05420j

**Published:** 2017-01-30

**Authors:** Sarit S. Agasti, Yu Wang, Florian Schueder, Aishwarya Sukumar, Ralf Jungmann, Peng Yin

**Affiliations:** a Wyss Institute for Biologically Inspired Engineering , Harvard University , Boston , Massachusetts , USA . Email: py@hms.harvard.edu ; Email: jungmann@biochem.mpg.de; b Department of Systems Biology , Harvard Medical School , Boston , Massachusetts , USA; c New Chemistry Unit and Chemistry & Physics of Materials Unit , Jawaharlal Nehru Centre for Advanced Scientific Research (JNCASR) , Bangalore , India; d Program of Biological and Biomedical Science , Harvard Medical School , Boston , Massachusetts , USA; e Department of Physics and Center for Nanoscience , Ludwig Maximilian University , 80539 Munich , Germany; f Max Planck Institute of Biochemistry , 82152 Martinsried near Munich , Germany

## Abstract

Recent advances in super-resolution fluorescence imaging allow researchers to overcome the classical diffraction limit of light, and are already starting to make an impact in biology. However, a key challenge for traditional super-resolution methods is their limited multiplexing capability, which prevents a systematic understanding of multi-protein interactions on the nanoscale. Exchange-PAINT, a recently developed DNA-based multiplexing approach, in theory facilitates spectrally-unlimited multiplexing by sequentially imaging target molecules using orthogonal dye-labeled ‘imager’ strands. While this approach holds great promise for the bioimaging community, its widespread application has been hampered by the availability of DNA-conjugated ligands for protein labeling. Herein, we report a universal approach for the creation of DNA-barcoded labeling probes for highly multiplexed Exchange-PAINT imaging, using a variety of affinity reagents such as primary and secondary antibodies, nanobodies, and small molecule binders. Furthermore, we extend the availability of orthogonal imager strands for Exchange-PAINT to over 50 and assay their orthogonality in a novel DNA origami-based crosstalk assay. Using our optimized conjugation and labeling strategies, we demonstrate nine-color super-resolution imaging *in situ* in fixed cells.

## Abstract 2

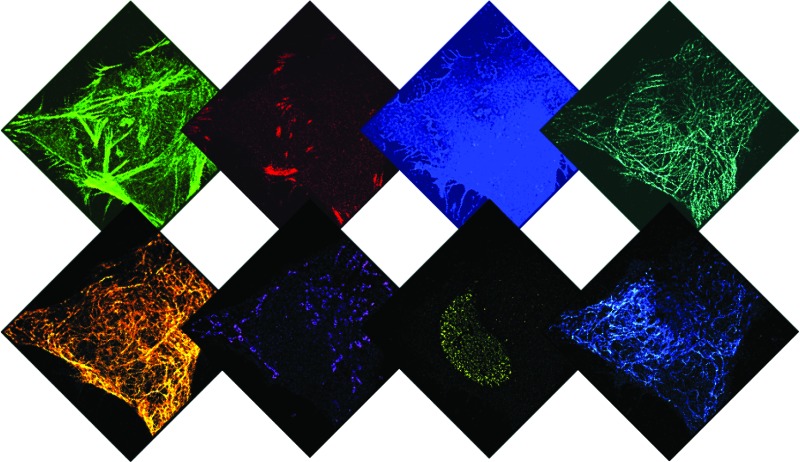
We report the development of multiplexed cellular super-resolution imaging using DNA-barcoded binders.

## Introduction

Fluorescence microscopy has become a standard method for *in situ* characterization of molecular details in both biological and clinical samples. Compared to complementary characterization methods such as electron microscopy,^1^ fluorescence imaging allows the efficient and specific detection of targets like proteins or nucleic acids using affinity labeling reagents such as antibodies.^2^ However, the spatial resolution of conventional fluorescence microscopy is limited, by the diffraction limit of light, to ∼200 nm. Large efforts have been devoted to overcome this limitation, resulting in a number of so-called super-resolution methods that can nowadays readily achieve sub-20 nm resolution in cells.^3^ Most super-resolution microscopy techniques, such as Structured Illumination Microscopy (SIM),^4^ Stimulated Emission Depletion (STED) microscopy,^5^ (fluorescent) Photo-Activated Localization Microscopy ((f)PALM)^6,7^ and (direct) Stochastic Optical Reconstruction Microscopy ((d)STORM),^8,9^ to this date rely on target labeling using static or fixed fluorescent tags. This labeling is usually achieved *via* either genetically encoded fusion proteins (PALM) or immunolabeling using dye-conjugated antibodies (STED, STORM). While these super-resolution approaches have already enabled new biological findings, some limitations persist. Two of the major limitations of single-molecule localization-based techniques such as PALM or STORM are the hard-to-control photophysical properties of fluorophores and the limited photon budget of fixed target labels.

**Fig. 1:**
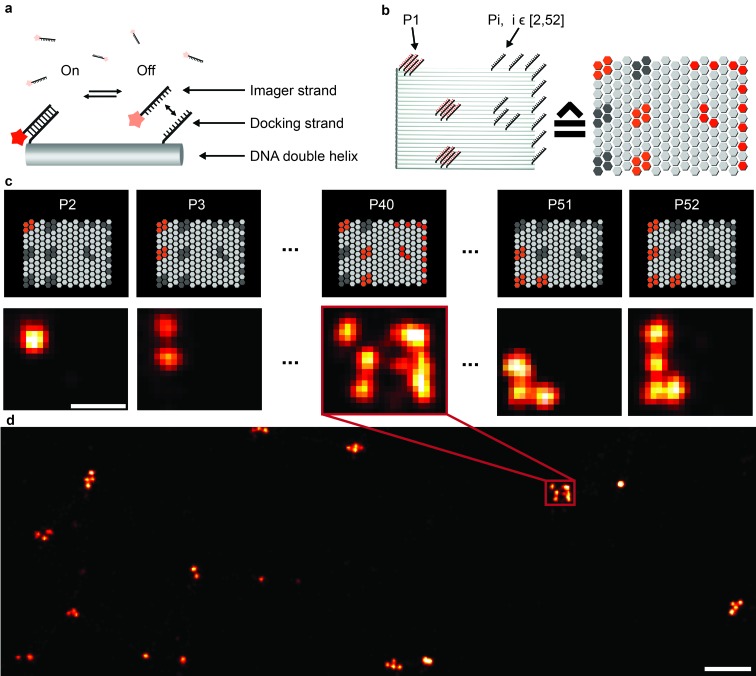
Crosstalk experiment to check the orthogonality of 52 docking sequences. (a) DNA origami carries single-stranded extensions (docking strands), which can transiently bind fluorescently labeled oligonucleotides (imagers) in solution. (b) Rectangular origami with modified extended staples (left side); a schematic representation of the structure is located on the right side; each hexagon represents a staple position that can be extended for DNA-PAINT imaging. Each origami contains a unique 6-bit barcode, addressable with the sequence P1 (left side), and single-stranded extensions that will act as docking sites for the imagers to be tested (P2–P52). Together, these extensions form a mirrored “F” shape (right side). (c) Crosstalk check for sequence P40. The upper row shows schematic representations of the barcode structures for each sequence. The bottom row shows the experimental data. The mirrored “F” appears only next to the barcode for the P40 sequence. This shows the orthogonality of the P40 sequence to all other sequences. (d) Overview image of the crosstalk experiment for P40. Scale bars: 50 nm (c), 200 nm (d).

A different approach to create “blinking” target molecules is implemented in the so-called Points Accumulation in Nanoscale Topography (PAINT) technique.^10^ In this technique, fluorescently labeled ligands freely diffuse in solution and bind either statically or transiently to targets of interest.^10,11^ This binding is detected as an apparent “blinking” of the target molecule or structure of interest. This enables the decoupling of blinking from the photophysical dye switching properties and thus alleviates one issue of STORM or PALM. However, the binding of diffusing ligands to their targets is achieved by electrostatic or hydrophobic interactions and is thus hard to program for different target species in a single cell, thus preventing easy-to-implement multiplexed detection. DNA-PAINT,^12–17^ a variation of PAINT, achieves stochastic switching of fluorescence signals between the ON- and OFF-states by the repetitive, transient binding of short fluorescently labeled oligonucleotides (“imager” strands) to complementary “docking” strands that are conjugated to targets (Fig. 1a). Upon binding of an imager strand, its fluorescence emission is detected and subsequently localized with nanometer precision. Importantly, the transient binding properties of these short DNA strands enable the facile removal of imager strands. Hence, orthogonal imager strands can be used to sequentially visualize multiple targets of interest. This so-called Exchange-PAINT^15^ approach in principle enables the spectrally-unlimited multiplexed super-resolution imaging of potentially hundreds of target molecules in the same sample, in a simpler and more straightforward fashion than other multiplexing approaches,^18–22^ such as those based on sequential immunostaining, imaging, and dye bleaching or inactivation.

The original Exchange-PAINT study demonstrated sequential 4-color imaging of cellular protein targets labeled with DNA-modified antibodies using different imager strands conjugated with a single-color dye. While successful, this labeling approach was based on biotinylated primary antibodies in combination with streptavidin and biotinylated docking strands to form an ‘antibody-streptavidin-DNA’ sandwich. This labeling procedure leads to two disadvantages; on one hand, the ‘linkage-error’, that is, the distance between the true target and labeled DNA docking site, is increased due to the addition of streptavidin, which ultimately leads to a localization offset from the true target position.^23^ On the other hand, the large sizes of these complexes leads to steric hindrance in the labeling process, which impedes the achievable labeling density and efficiency. Both of these effects can reduce the achievable spatial resolution.

Here, we introduce a general framework for labeling protein targets using DNA-PAINT docking strands, which are directly coupled to various labeling probes, thus addressing the aforementioned issues. First, we design and evaluate the performance and orthogonality of 52 DNA sequences for Exchange-PAINT. Next, we directly conjugate DNA oligonucleotides to antibodies, avoiding the biotin–streptavidin sandwich, and then extend the platform to small-sized binders, including nanobodies and small molecules, to further enhance the achievable labeling density and spatial accuracy. Finally, we successfully use our labeling platform to demonstrate nine-target super-resolution imaging in fixed biological samples.

## Results and discussion

### Design of >50 orthogonal imager strands and DNA origami crosstalk assay

To extend the multiplexing capabilities of Exchange-PAINT, we designed 37 sequences in addition to the previously published 15 strands,^15^ to theoretically enable 52-plex super-resolution imaging. We started with strand design using the “CANADA” software,^24^ employing the following conditions: the length of the docking site is 9 base pairs (bp), the GC-content is 40% (3 out of these 9), and there should be no sequence homology with more than 3 bases. To ensure the experimental orthogonality of the designed sequences and to test their performances in DNA-PAINT (*e.g.* achievable resolution), we conducted a series of 52 *in vitro* experiments (Fig. 1). We designed 52 unique barcoded DNA origami structures. Fig. 1b shows an example of one of these “barcodes”. In the schematic representation of the structure (Fig. 1b, right), each hexagon represents a potential DNA-PAINT docking site. The left-hand side of the origami structure features a 6-bit barcode, which is unique for each of the 52 origami structures. The barcode staple strands are universally extended with the DNA-PAINT sequence P1 for all structures. The right-hand side of each origami structure carries a geometric pattern, created with unique docking strand sequences for each of the 52 barcoded origami structures, *i.e.* docking strands Pi, with *i* ∈ [2, 52]. The crosstalk experiment was then conducted as follows. We prepared 52 samples, all of which contained 52 barcoded DNA origami structures and imager strand species P1 in solution. Furthermore, each unique sample additionally contained one orthogonal DNA-PAINT imager sequence out of the remaining 52 imagers in the sequence pool (*i.e.* either P2, P3, P4, and so on). As an example, the result for the experiment with the imager sequence P40 is depicted in Fig. 1c. If there is no crosstalk, the motive on the right side of the structure is only visible for one origami species with the 6-bit barcode for sequence P40. For the remaining structure, only the respective 6-bit barcode is visible, imaged with P1. A larger view image is shown in Fig. 1d, underlining the fact that there is indeed no crosstalk between the imager strand P40 and all remaining sequences. This experiment was repeated 51 times for the remaining sequences and resulted in no detectable crosstalk of all 52 imager strands (see ESI Table 1† for the DNA origami sequences, ESI Table 2† for the imager sequences, ESI Fig. 1† for a schematic overview of all 52 barcoded DNA origami structures, and ESI Fig. 2–52† for the respective crosstalk imaging rounds).

### Synthesis of direct DNA-antibody conjugates for DNA-PAINT imaging

To translate this large multiplexing capability *in situ*, we next describe the synthesis of barcoded DNA-antibody conjugates for DNA-PAINT imaging. Antibodies (Abs) are the most widely used labeling probes. High specificity and affinity for target antigens coupled with the large repertoire of commercially available antibodies make them an integral component in life science research.

They are routinely used in diverse immunoassay applications, including immunofluorescence (IF) imaging and immunohistochemistry (IHC). These attributes prompted us to first adopt the antibody-labeling platform for DNA-PAINT imaging and develop a general DNA conjugation approach that builds upon the vast array of available antibodies to complement the high multiplexing capability of Exchange-PAINT.

**Fig. 2:**
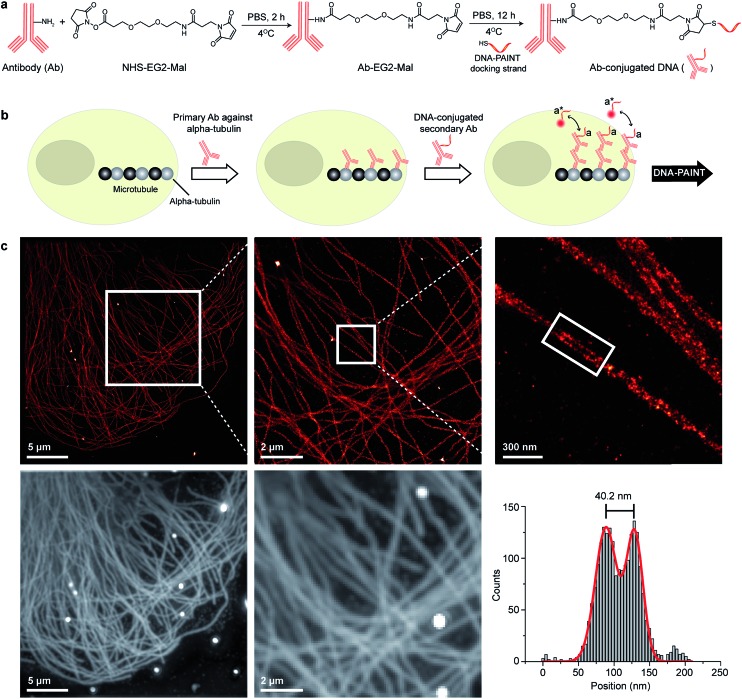
Antibody-DNA conjugation method and super-resolution imaging with a DNA-conjugated secondary antibody. (a) Synthesis scheme for DNA-conjugated antibody preparation. Note that SM(PEG)_2_ is depicted here as NHS-EG2-Mal. (b) Labeling strategy for the DNA-conjugated secondary antibody. (c) Secondary antibody-based DNA-PAINT super-resolution imaging of microtubules inside a fixed BSC-1 cell. Zooming in of the highlighted area shows the resolution improvement compared to the diffraction-limited micrographs of the same area. The cross-sectional histogram of a hollow microtubule structure clearly shows two distinct lines with a separation of ∼40 nm. This is in good agreement with earlier reports.^26^

There are a few criteria to be considered for selecting antibody conjugation methods for DNA-PAINT. Firstly, the conjugation chemistry should be versatile such that it is applicable to various antibody isotypes. Secondly, the method should work for commercially available antibodies. Hence, conjugation techniques involving technically involved genetic engineering of antibodies, such as unnatural amino acid incorporation, are not favored. Lastly, the method should be simple, economical, high yield and easily accessible to researchers. Based on these criteria, we chose to conjugate thiol-modified DNA oligonucleotides to lysine residues on antibodies *via* SM(PEG)_2_ (PEGylated succinimidyl 4-(*N*-maleimidomethyl) cyclohexane-1-carboxylate) crosslinkers (Fig. 2a), and optimized the protocol for DNA-PAINT imaging. In this strategy, the small ‘footprint’ of SM(PEG)_2_ ensures minimum steric hindrance for antigen binding while placing the DNA label in close proximity to the antibody in order to achieve high-resolution. In addition, the use of the PEG spacer helps to reduce nonspecific binding.^25^ For conjugation, a phosphate-buffered solution of antibody was first incubated with SM(PEG)_2_ crosslinkers. In this step, the *N*-hydroxysuccinimide (NHS) ester group of SM(PEG)_2_ reacts with the amine groups present on the lysine residues and anchors the maleimide group on the antibody surface. After removing the excess cross-linker using size-exclusion chromatography, maleimide-functionalized antibodies were reacted with thiol-modified DNA oligonucleotides to form stable DNA-antibody conjugates. The antibody conjugates were purified using a molecular weight cut-off filter (100 kDa). The successful conjugation of DNA strands to antibodies was verified using matrix-assisted laser desorption/ionization-mass spectrometry (MALDI-MS). We have optimized the protocols to yield conjugates with close to 1 DNA label/Ab (ESI Fig. 53† and the corresponding calculation based on the MALDI mass shift). Limiting the number of conjugated DNA oligonucleotides per antibody has two advantages: first, it helps to decrease nonspecific binding, which is potentially mediated by interactions of conjugated DNA with other cellular compartments; secondly it reduces the probability that lysine residues in the antigen recognition sites are labeled with DNA, which could otherwise decrease the antigen binding affinity. It should be noted that even though only about one DNA oligonucleotide is conjugated per Ab on average, a close to 100% label readout could still be achieved by DNA-PAINT, as imager strands are continuously targeting the docking strand on the antibody, leading to high imaging efficiency. This is in contrast to traditional imaging methods, which use fluorophore-conjugated antibodies, where photobleaching of a low density of fluorophores can lead to a loss of target visualization due to insufficient sampling.

A detailed step-by-step description of the preparation of DNA-antibody conjugates and subsequent characterization can be found in ESI Protocols 1 and 2.†

### DNA-PAINT imaging with DNA-conjugated secondary antibodies

To test the super-resolution imaging capabilities of the directly conjugated antibody probes, we first used a DNA-conjugated secondary antibody and performed single-color DNA-PAINT imaging of the microtubule network in BSC-1 cells. Among the various fibrous cytoskeleton protein networks, microtubules were selected as a model system for evaluation of the imaging performance due to their well-defined structure, shape and importantly their nanoscale, subdiffraction dimensions (diameter ∼25 nm).^23^ To perform DNA-PAINT imaging, at first we fixed the microtubule network in the BSC-1 cells using methanol and stained it with primary antibodies against alpha-tubulin followed by the DNA-conjugated secondary antibody (Fig. 2b). Next, DNA-PAINT imaging was performed using ATTO655-conjugated imager strands and using highly inclined and laminated optical sheet (HILO) illumination. Afterwards, a super-resolved DNA-PAINT image was reconstructed using a custom spot-finding and 2D-Gaussian fitting algorithm. In addition, fiducial-based drift correction was performed using gold nanoparticles to compensate for any sample movement during image acquisition.

As shown in Fig. 2c, the resulting DNA-PAINT image shows a significant resolution increase compared to the diffraction-limited representation. The increased resolution could be easily observed by visualizing a dense region of the microtubule network where individual microtubule filaments could be clearly distinguished, which are impossible to distinguish in the standard diffraction-limited micrograph. More importantly, when a single microtubule fiber was magnified, DNA-PAINT was able to resolve the hollow tubular structure.^26^ This underlines a substantial improvement of the labeling density and size over previously published DNA-PAINT cell data,^15^ where biotin–streptavidin-mediated DNA conjugated antibodies failed to resolve this hollow tubular structure. To semi-quantitatively assess the achievable resolution, we measured the cross-sectional profile of localizations of a “hollow” microtubule structure. As depicted in Fig. 2c, the cross-sectional profiles show two well-resolved peaks with a separation of ∼40 nm between them, which is in good agreement with the previous reports.^27^ We also tested our direct DNA-conjugated antibodies for dual-color super-resolution imaging (ESI Fig. 54†). Here, we co-stained Tom20, a mitochondrial outer membrane protein, and HSP60, a mitochondrial matrix protein in fixed HeLa cells. The image was taken using ATTO655- and Cy3B-conjugated imager strands for Tom20 and HSP60, respectively. This dual-color DNA-PAINT image shows Tom20 localizing on the outer mitochondrial membrane, while HSP60 localizes on the inside of the mitochondria.

### DNA-PAINT imaging with DNA-conjugated primary antibodies

Although secondary antibodies are widely used as indirect immunostaining approaches, they are not the ideal choice for highly multiplexed super-resolution imaging for two primary reasons, one of which is the limited availability of primary antibodies from different species. Additionally, due to the increased size of the primary-secondary antibody sandwich, the resulting larger ‘linkage-error’ could lead to lower spatial accuracy. Therefore, we next turned to more direct immunostaining approaches, involving only primary antibodies.

**Fig. 3:**
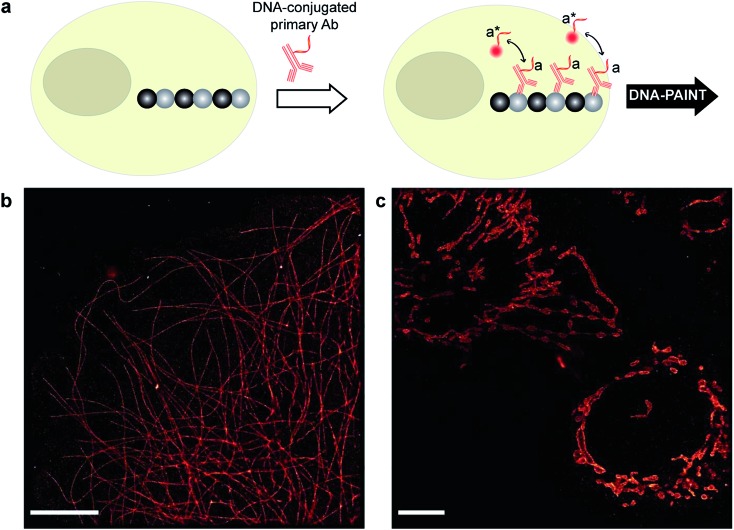
DNA-PAINT imaging with a DNA-conjugated primary antibody. (a) Labeling scheme with a DNA-conjugated primary antibody. (b) Primary antibody-based DNA-PAINT imaging of microtubules inside a fixed BSC-1 cell. (c) Primary antibody-based DNA-PAINT imaging of Tom20 in mitochondria. Tom20 localizes to the mitochondrial membrane, which is clearly resolved. Scale bars: 5 μm.

To test primary antibody-based DNA-PAINT imaging, we used two model systems: microtubules and mitochondria. The microtubule network was stained with DNA-conjugated primary antibodies against alpha-tubulin, whereas the mitochondria were stained for Tom20 (Fig. 3a). Fig. 3b and c show the resulting DNA-PAINT images of directly-labeled microtubules and mitochondria structures, respectively. As can be seen in Fig. 3b, individual microtubules are clearly visible in the super-resolved image, similar to the image obtained using secondary antibody-based staining. On the other hand, as shown in Fig. 3c, the DNA-PAINT imaging revealed the outer mitochondrial membrane localization of the Tom20 protein.

### DNA-PAINT imaging with DNA-conjugated nanobodies

IgG antibodies, typically used in immunofluorescence studies, are ∼150 kDa in MW and ∼10 nm in size. Although the large commercially available repertoire of antibodies is advantageous for their use in highly multiplexed imaging, their rather large sizes are ultimately a concern when highly accurate localization of the target is necessary or when high density labeling^23,28^ is required for molecular counting. To address this issue, we used antibody-like affinity molecules with smaller sizes, including nanobodies and high affinity small molecule binders. Nanobodies are derived from heavy chain-only antibodies generated by camelids.^29^ They are small in size (∼1.5 nm × 2.5 nm), only ∼13 kDa in MW and have high affinity for their target molecule.^23,28^ Previous reports have demonstrated the enhanced resolving power of nanobodies for super-resolution imaging of microtubules.^23,30^

**Fig. 4:**
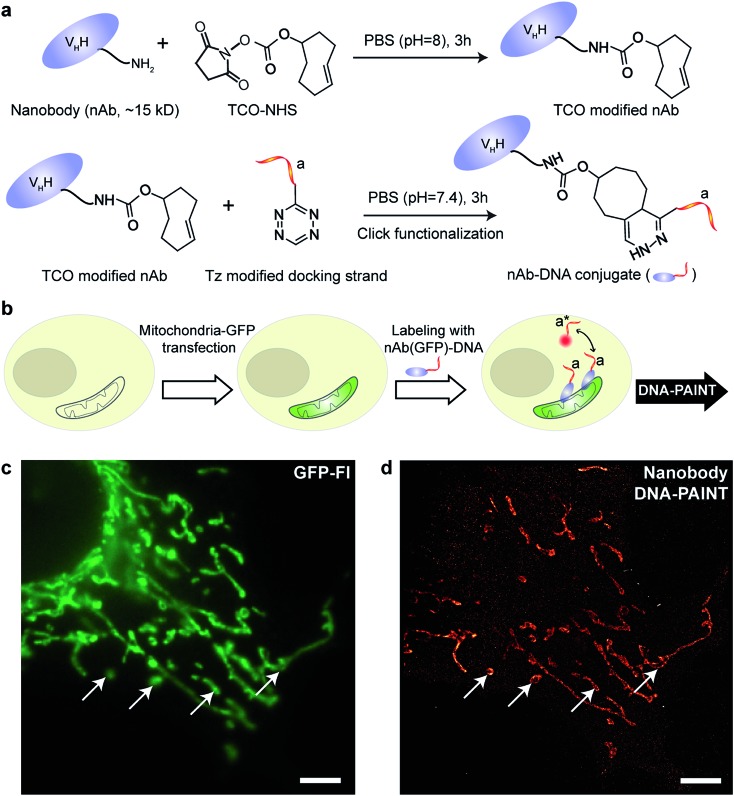
Synthesis of DNA-conjugated nanobodies for DNA-PAINT imaging. (a) Synthesis scheme for DNA-conjugated nanobody preparation. (b) Labeling scheme using the DNA-conjugated nanobody. (c, d) Nanobody-based DNA-PAINT super-resolution imaging of the mitochondrial network inside a fixed HeLa cell. A comparison of the diffraction-limited image (c) to the DNA-PAINT image (d) underlines the achieved resolution increase. Scale bars: 5 μm.

We began with optimizing the conjugation chemistry for DNA-labeled nanobodies. We used a cycloaddition reaction between 1,2,4,5-tetrazine (Tz) and *trans*-cyclooctene (TCO) to couple DNA-PAINT docking strands to a model anti-GFP nanobody (Fig. 4a). The strain-promoted [4+2] cycloaddition reaction between Tz and TCO is fast with a rate constant of up to 10^6^ (Ms)^–1^, quantitative and can proceed in physiological conditions, which helps to rapidly and efficiently conjugate DNA while preserving the functionality of the nanobodies.^31^ In brief, a TCO-NHS ester was used to react with the primary amine groups of lysine residues on nanobodies in PBS (pH = 8) for 3 hours. Simultaneously, amine-modified DNA-PAINT docking strands were reacted with Tz and subsequently purified using HPLC. The TCO-modified nanobodies were then coupled with the Tz-modified DNA-PAINT docking strands during a reaction in PBS (pH = 7.4) for 3 hours. As for the case of antibody conjugation discussed above, we have optimized the protocols to yield conjugates with close to 1 DNA label/nanobody (see ESI Fig. 55† and the corresponding calculation based on the MALDI mass shift).

Next, we tested the performance of our DNA-conjugated nanobodies for DNA-PAINT super-resolution imaging in HeLa cells expressing the mitochondria-green fluorescent protein (GFP). HeLa cells were transfected with a baculoviral vector containing mitochondrial leader sequence-fused GFP (BacMam2.0),^32^ and the expression of GFP was detected after 2 days of transfection (Fig. 4b and c). The transfected cells were stained with DNA-conjugated anti-GFP nanobodies after PFA fixation. The DNA-PAINT image (Fig. 4d) shows a specific signal and a clear resolution increase when resolving the mitochondrial structures. The shape of the mitochondria as detected with DNA-PAINT (Fig. 4d) correlated well with their corresponding GFP signals detected using conventional fluorescence microscopy (Fig. 4c). We note that some mitochondria in Fig. 4c did not show up in Fig. 4d. These “missing” mitochondria were actually out-of-focus when imaged in HILO mode, and hence did not generate enough localization events for super-resolution image reconstruction.

A step-by-step protocol for nanobody-DNA conjugation is described in ESI Protocol 3.†

### DNA-PAINT imaging with small molecule binders

Small molecule binders represent another important class of targeting reagents for high-density protein labeling. To test the compatibility of DNA-PAINT imaging with small molecule probes, we selected phalloidin, a bicyclic heptapeptide, to selectively target F-actin.^33^ Actin filaments are usually present in high density in cells with individual fiber diameters as small as 5–10 nm.^34^ Imaging of the actin cytoskeleton structure using DNA-conjugated phalloidin probes will not only allow investigation of the compatibility of small molecule probes with DNA-PAINT, but also demonstrate the benefit of employing a smaller targeting agent to resolve high density sub-10 nm structures using DNA-based imaging.

**Fig. 5:**
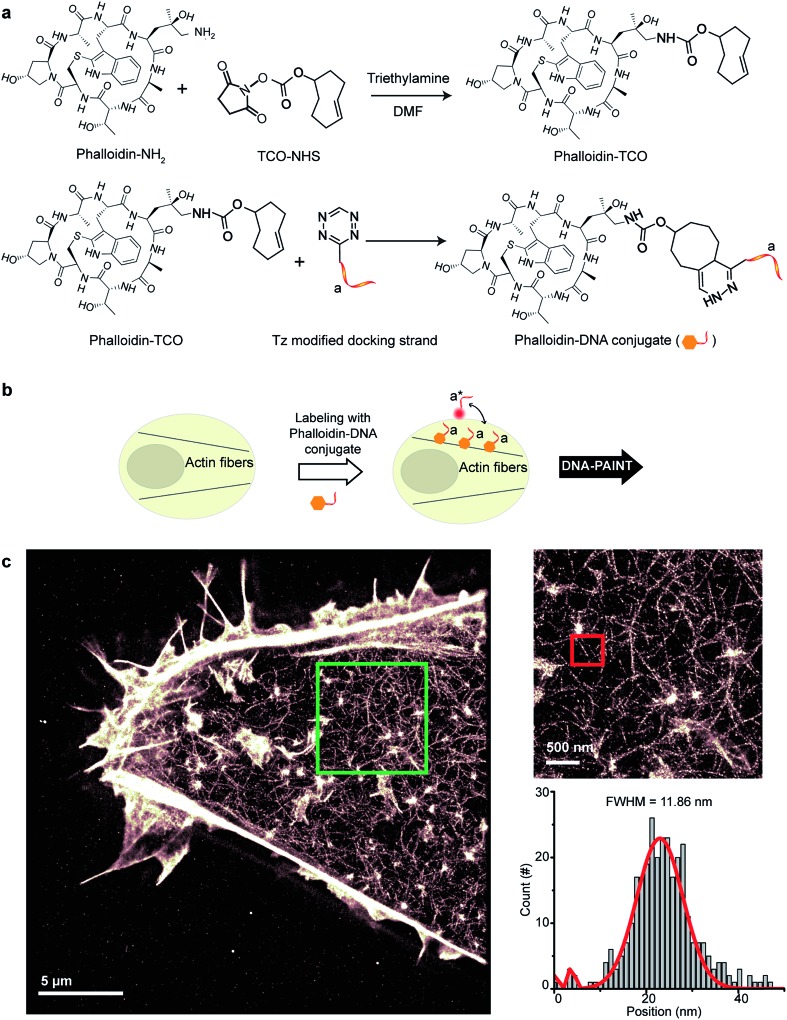
Conjugation of DNA oligos to phalloidin for actin imaging with DNA-PAINT. (a) Synthesis scheme for DNA-conjugated phalloidin. (b) Labeling strategy for phalloidin using the DNA-phalloidin conjugate. (c) Resulting DNA-PAINT image of the actin network inside a fixed HeLa cell. Zooming in to the highlighted area (green) highlights the achievable resolution. A Gaussian distribution was fitted to the cross-sectional histogram of an actin fiber (selected from the highlighted red region). FWHM of the distribution: ∼12 nm.

To create DNA-conjugated phalloidin probes, we used the Tz and TCO-based conjugation method (Fig. 5a), similar to the method described for the nanobodies. Here, a TCO-NHS ester was first reacted with amine-modified phalloidin molecules to form a phalloidin-TCO conjugate. After HPLC purification, the TCO-phalloidin conjugates were incubated with Tz-modified DNA-PAINT docking strands to form DNA-phalloidin conjugates, whose identity was then verified using MALDI-MS spectroscopy (see ESI Fig. 56†).

We tested the performance of our DNA-phalloidin probes in HeLa cells. To preserve the cytoskeleton ultrastructure, we fixed HeLa cells using 0.1% glutaraldehyde along with 3% PFA. Staining of the actin filaments was achieved by incubating cells with 1 μM of DNA-phalloidin probes (Fig. 5b). After removing excess probes, DNA-PAINT imaging was performed using ATTO655-labeled imager strands. Fig. 5c shows the super-resolved DNA-PAINT image of actin cytoskeletons, where the individual actin filaments are well resolved and clearly visible. For a more “quantitative” determination of the imaging resolution, we measured the cross-section of a single filament (Fig. 5c), yielding an apparent filament width of ∼12 nm (FWHM), a dimension which is consistent with earlier reports.^35^

A step-by-step protocol for Phalloidin-DNA conjugation is described in ESI Protocol 4.†

### Highly multiplexed Exchange-PAINT imaging using a pool of orthogonally labeled antibodies

Protein interaction networks mediate cellular responses to various environmental stimuli. It is increasingly evident that the spatial heterogeneity of protein distribution in cells leads to intracellular functionality differences among distinct compartments and intercellular variance among cells located in different regions. Mapping the heterogeneity of protein networks is challenging for three reasons: (1) the location information of proteins needs to be well preserved; (2) comprehensive studies probing multiple protein targets need to be performed in order to understand the whole network; (3) high spatial accuracy is required to achieve subcellular mapping, rendering conventional diffraction-limited fluorescence imaging unsuitable.

The development of Exchange-PAINT imaging enables highly multiplexed super-resolution detection in single cells and is hence desirable for protein network mapping directly *in situ*. By synergistically combining optimized DNA probe design and improved DNA-antibody conjugation, we here report the thus far unprecedented nine-target super-resolution imaging in biological samples.

**Fig. 6:**
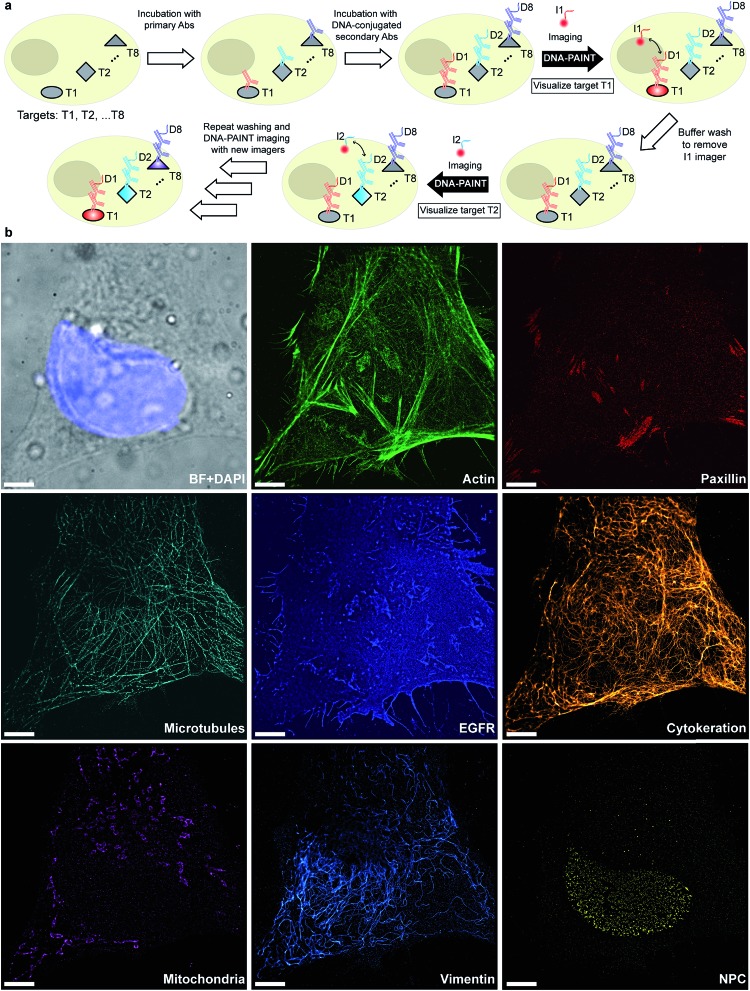
Secondary antibody-based labeling for multiplexing with Exchange-PAINT. (a) Schematic representation of Exchange-PAINT. Target proteins (T1···T8) are labeled with DNA (D1···D8)-conjugated secondary antibodies using an indirect immunostaining approach. Complementary ATTO655-dye-labeled DNA strands (I1···I8) are sequentially applied to the sample. Post-acquisition, a washing buffer with reduced ionic strength was used to efficiently remove the imagers. Eight imaging rounds were performed using orthogonal imager strands with the same dye. (b) Eight-target DNA-PAINT image of fixed HeLa cells acquired in eight sequential rounds. Scale bars: 5 μm.

Given that indirect immunostaining approaches are most widely used and present a cost-effective method for labeling protein targets, we first tested the multiplexed super-resolution imaging using DNA-conjugated secondary antibodies. Here, we stained fixed HeLa cells with phalloidin and primary antibodies from seven different species followed by DNA-conjugated secondary antibodies from the donkey species. Seven rounds of probe exchange were performed to image all eight targets. The results showed that eight cellular structures could be clearly visualized with Exchange-PAINT, and there was minimal-to-no crosstalk of signals among the different antibodies (Fig. 6). It can be seen that paxillin localized at the tip of the actin filaments, which is consistent with the fact that paxillin is an actin regulation protein in focal adhesions.^36^ The nuclear pore complex signal was present specifically in the nucleus which was indicated by DAPI staining of the nucleus.

**Fig. 7:**
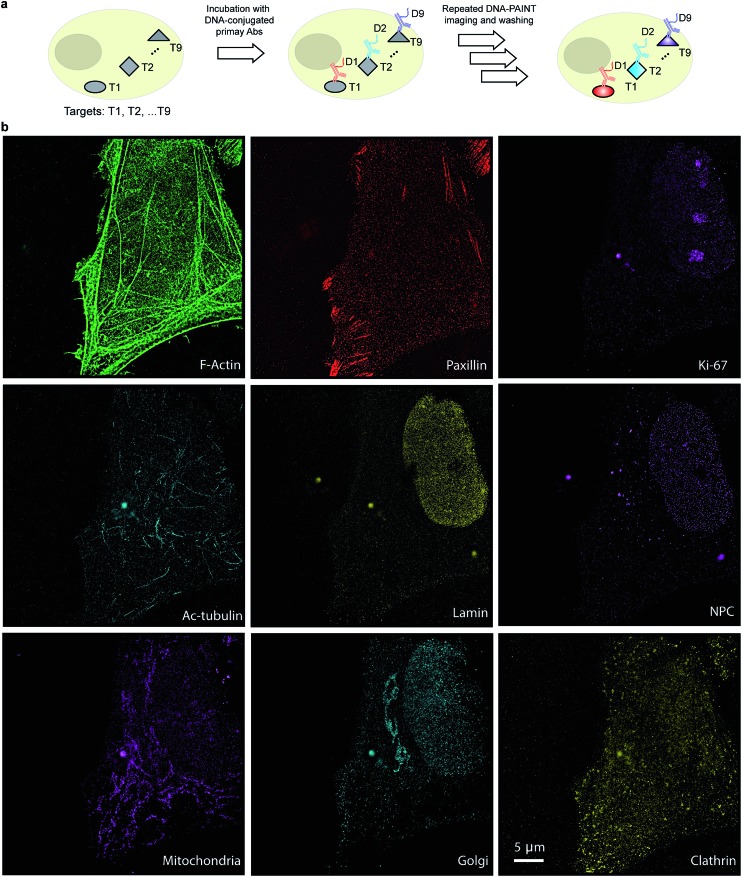
Primary antibody-based labeling for multiplexing with Exchange-PAINT. (a) Labeling strategy for primary antibody-based imaging. The target proteins (T1···Tn) were labeled with DNA (D1···Dn)-conjugated primary antibodies using a direct immunostaining approach. Complementary imager strands (labeled with ATTO655) were sequentially introduced to the sample for super-resolution imaging as before. Post-acquisition, a washing buffer with reduced ionic strength was introduced to remove all imagers. Nine imaging rounds were performed using orthogonal imager strands conjugated to the same dye. (b) Nine-target super-resolution image of proteins in fixed HeLa cells acquired using nine rounds of Exchange-PAINT.

The use of secondary antibodies for multiplexed detection, however, is limited by the availability of primary antibodies from different species. Therefore, we next used directly DNA-labeled primary antibodies and small molecule binders, and achieved nine-target super-resolution visualization (Fig. 7). Nuclear protein Ki67 signals were mostly located in the nucleus while Lamin and Nuclear Pore Complex (NPC) marked the nuclear membrane. Clathrin signals indicated the distribution of coated-vesicles in the cytoplasm. We note that the super-resolution signal in the reconstructed images obtained using primary antibodies was lower compared to the signal obtained by indirect labeling using secondary antibodies, which is expected due to the lack of signal amplification in the primary antibody only case. We anticipate that this fact can be improved by increasing the imaging time to obtain more localization events. This, again, is unique to Exchange-PAINT, due to its resistance to photobleaching and replenishable imaging probes.

Detailed information regarding the primary and secondary antibodies and imager sequences can be found in ESI Tables 3–7.† The immunostaining protocols with PFA, PFA and glutaraldehyde, and methanol are detailed in ESI Protocols 5–7.†

## Conclusion

In summary, we have developed a versatile labeling platform for the conjugation of DNA oligonucleotides to various labeling probes for DNA-PAINT and Exchange-PAINT with high labeling density, spatial accuracy and achievable resolution. We also demonstrated the use of our labeled probes for highly multiplexed imaging in biological samples with nanoscale resolution. The conjugation method is efficient and simple to implement, and should be easily adopted in common biological labs. We anticipate that the conjugation methods developed here can make Exchange-PAINT accessible to a broader scientific community, and will consequently be used to solve more complex biological questions.

## References

[cit1] Knoll M., Ruska E. (1932). Z. Phys..

[cit2] Coons A. H., Creech H. J., Jones R. N. (1941). Exp. Biol. Med..

[cit3] Hell S. W. (2015). J. Phys. D: Appl. Phys..

[cit4] Gustafsson M. G. (2000). J. Microsc..

[cit5] Hell S. W., Wichmann J. (1994). Opt. Lett..

[cit6] Hess S. T., Girirajan T. P. K., Mason M. D. (2006). Biophys. J..

[cit7] Betzig E. (2006). Science.

[cit8] Heilemann M. (2008). Angew. Chem., Int. Ed..

[cit9] Rust M. J., Bates M., Zhuang X. (2006). Nat. Methods.

[cit10] Sharonov A., Hochstrasser R. M. (2006). Proc. Natl. Acad. Sci. U. S. A..

[cit11] Giannone G. (2010). Biophys. J..

[cit12] Jungmann R. (2010). Nano Lett..

[cit13] Lin C. (2012). Nat. Chem..

[cit14] Iinuma R. (2014). Science.

[cit15] Jungmann R. (2014). Nat. Methods.

[cit16] Jungmann R. (2016). Nat. Methods.

[cit17] Dai M., Jungmann R., Yin P. (2016). Nat. Nanotechnol..

[cit18] Duose D. Y., Schweller R. M., Hittelman W. N., Diehl M. R. (2010). Bioconjugate Chem..

[cit19] Schweller R. M. (2012). Angew. Chem., Int. Ed..

[cit20] Tam J., Cordier G. A., Borbely J. S., Sandoval Álvarez A., Lakadamyali M. (2014). PLoS One.

[cit21] Valley C. C., Liu S., Lidke D. S., Lidke K. A. (2015). PLoS One.

[cit22] Yi J. (2016). Mol. Biol. Cell.

[cit23] Ries J., Kaplan C., Platonova E., Eghlidi H., Ewers H. (2012). Nat. Methods.

[cit24] Feldkamp U. (2010). J. Comput. Chem..

[cit25] He Q. (2010). Biomaterials.

[cit26] Dempsey G. T., Vaughan J. C., Chen K. H., Bates M., Zhuang X. (2011). Nat. Methods.

[cit27] Bates M., Huang B., Dempsey G. T., Zhuang X. (2007). Science.

[cit28] Rothbauer U. (2006). Nat. Methods.

[cit29] Muyldermans S. (2009). Vet. Immunol. Immunopathol..

[cit30] Mikhaylova M. (2015). Nat. Commun..

[cit31] Haun J. B., Devaraj N. K., Hilderbrand S. A., Lee H., Weissleder R. (2010). Nat. Nanotechnol..

[cit32] Kost T. A., Condreay J. P., Ames R. S., Rees S., Romanos M. A. (2007). Drug Discovery Today.

[cit33] Wulf E., Deboben A., Bautz F. A., Faulstich H., Wieland T. (1979). Proc. Natl. Acad. Sci. U. S. A..

[cit34] Grazi E. (1997). FEBS Lett..

[cit35] Xu K., Babcock H. P., Zhuang X. (2012). Nat. Methods.

[cit36] Turner C. E. (2000). J. Cell Sci..

