# Supplementary material for: DNA-barcoded labeling probes for highly multiplexed Exchange-PAINT imaging
Source: Chem Sci. 2017 Jan 30;8(4):3080–91. doi: 10.1039/c6sc05420j (PMC5380918; doi:10.1039/c6sc05420j)
Supplement: Supplementary file 1 [file SC-008-C6SC05420J-s001.pdf]

Electronic Supplementary Material (ESI) for Chemical Science.
This journal is © The Royal Society of Chemistry 2017

Supplementary Information

Materials and Methods

Materials

Unless otherwise stated, all chemicals and solvents were purchased from commercial suppliers and used as received. PEGylated SMCC (succinimidyl 4-(N-maleimidomethyl)cyclohexane-1-carboxylate) cross-linker (SM(PEG)$_2$), trans-4-Cyclooctеn-1-yl 2,5-dioxo-1-pyrrolidinyl carbonate (TCO-NHS) and benzylamino tetrazine N-hydroxysuccinimidyl ester (Tz-NHS) were purchased from Sigma-Aldrich. Commercial sources of the antibodies were listed in the **Supplementary Tables 3, 4,** and **6**. GFP nanobodies were purchased from ChromoTek. Amicon Ultra Centrifugal Filter (100 kDa MWCO) was purchased from Merck Millipore. Zeba spin desalting column (7000 MWCO) was purchased from Thermo Fisher Scientific. NAP-5 columns were purchased from GE Healthcare. Dulbecco's Phosphate-Buffered Saline (PBS, pH 7.4) without calcium and magnesium was purchased from Life Technologies. Unmodified, dye-labeled and biotinylated DNA oligonucleotides were purchased from Integrated DNA Technologies (IDT). Streptavidin was purchased from Invitrogen (catalog number: S-888). BSA-biotin was obtained from Sigma-Aldrich (catalog number: A8549). Coverslips were purchased from VWR (coverslips 18 × 18 mm, #1.5). The glass slides were purchased from VWR (25 × 75 × 1 mm). M13mp18 scaffold was obtained from New England BioLabs (N4040s). Freeze ‘N Squeeze columns were ordered from Bio-Rad (catalog number: 7326165).
The following buffers were used:

- Buffer A: 10 mM Tris-HCl, 100 mM NaCl, 0.05 % Tween 20, pH 7.5
- Buffer B: 5 mM Tris-HCl, 10 mM $MgCl_2$, 1 mM EDTA, 0.05 % Tween 20, pH 8.0

Microscope setup

Fluorescence imaging was carried out on an inverted Nikon Eclipse Ti microscope (Nikon Instruments) with the Perfect Focus System, applying an objective-type TIRF configuration with an oil-immersion objective (CFI Apo TIRF 100×, NA 1.49, Oil). For excitation of ATTO655 fluorophores, a 639 nm laser (150 mW nominal, Toptica iBeam Smart) was used. The laser beam was passed through a cleanup filter (ZET 642/20x, Chroma Technology, Bellows Falls, VT) and coupled into the microscope objective using a single-band beam splitter (ZT647rdc, Chroma Technology). Fluorescence light was spectrally filtered with an emission filter (ET6651p, ET705lp, Chroma Technology).
For excitation of Cy3B fluorophores, a 561 nm laser (200 mW nominal, Coherent Sapphire) was used. The laser beam was passed through a cleanup filter (ZET 561/10x, Chroma Technology, Bellows Falls, VT) and coupled into the microscope objective using a single-band beam splitter (ZT561rdc, Chroma Technology). Fluorescence light was spectrally filtered with an emission filter (ET600/50n, Chroma Technology). Single molecule fluorescence signals were imaged on an EMCCD camera (iXon Ultra 897 EMCCD, Andor Technology). Data acquisition was performed without additional magnification in the detection path and yielding a pixel size of 160 nm.

DNA origami self-assembly

DNA origami for crosstalk experiments were formed in a one-pot reaction with a 40 µl total volume containing 10 nM scaffold strand (M13mp18), 100 nM folding staples, 12 nM biotin-modified staples and 1000 nM DNA-PAINT docking strands in folding buffer (1× TE buffer with 12.5 mM $MgCl_2$). The solution was annealed using a thermal ramp cooling from 90 °C to 4 °C over the course of 3 h.

DNA origami sample preparation and imaging

For sample preparation, a piece of coverslip (No. 1.5, 18 × 18 mm , 0.17 mm thick) and a glass slide (3 × 1 inch^2, 1 mm thick) were sandwiched together by two strips of double-sided tape to form a flow chamber with inner volume of ≈20 µl. First, 20 µl of biotin-labeled bovine albumin (Sigma A8549, 1 mg/ml, dissolved in buffer A) was flown into the chamber and incubated for 2 min. The chamber was then washed using 40 µl of buffer A. 20 µl of streptavidin (Thermo, S888, 0.5 mg/ml, dissolved in buffer A) was then flown through the chamber and was allowed to bind for 2 min. After washing with 40 µl of buffer A and subsequently with 40 µl of buffer B, 20 µl of a mix of all 51 DNA origami multiplexing structures in buffer B (dilution of 1 in 25) were finally flown into the chamber and incubated for 2 min. The chamber was washed using 40 µl of buffer B. The final imaging buffer solution contained 3 nM ATTO655-labeled (P1) imager strands and 6 nM ATTO655-labeled (Pi, $i \in [2, 52]$) imager strands in buffer B. The chamber was sealed with epoxy before subsequent imaging. The EMCCD readout bandwidth was set to 3 MHz (no EM Gain) at 16 Bit. Imaging was performed using oblique illumination with an excitation intensity of ~300 W/cm^2 at 642 nm. Images were acquired for 10000 frames (200 ms integration time, total imaging time ~33 min).

Cell Culture

Cells (HeLa, BSC-1 and U2OS) were cultured in Dulbecco's Modified Eagle Medium (DMEM), supplemented with fetal bovine serum (FBS; 10%), penicillin and streptomycin (1%), and L-glutamine (1%). Cell lines were maintained at 37 °C in a humidified atmosphere containing 5% CO_2.

EGFR Transfection

At confluence, before plating, cells were washed, trypsinized and suspended in culture media. Cells were counted. In a typical experiment, ~50000 cells/well were plated in 8-well Nunc™ Lab-Tek™ Chamber Slides. 24 h post plating, when the cells achieved ~70% confluency, 2 µg or 4 µg of EGFR plasmid DNA along with P3000 were mixed together in 5 µl of Opti-MEM for each well in the chamber. At the same time, Lipofectamine 3000 transfection agent was mixed separately in 10 µl of Opti-MEM. The Lipofectamine and the DNA reagents were mixed in a 1:1 ratio and incubated at room temperature for 5 minutes to form complexes. This was added dropwise to cells and the cells were incubated at 37 °C in a humidified atmosphere containing 5% CO2. After 24 h, the media was replaced with Dulbecco's Modified Eagle Medium (DMEM), supplemented with fetal bovine serum (FBS; 10%), penicillin and streptomycin (1%), and L-glutamine (1%). Typically 48 h following transfection cells were used in the indicated assays.

CellLight Mitochondria-GFP, BacMam 2.0 Transfection

At confluence, before plating, HeLa cells were washed, trypsinized and suspended in culture medium. Cells were counted and ~50000 cells were plated in a Labtek Chamber. After 24 h, 10 µl of CellLight Mitochondria-GFP, BacMam 2.0 Transfection reagent was added to each well in the chamber and cells were incubated at 37 °C in a humidified atmosphere containing 5% CO_2. The transfection efficiency was checked 24 h after transfection and the cells were used typically 48 h after transfection.

Supplementary Protocols

Supplementary Protocol 1. Preparation of DNA-antibody conjugates

1. Antibodies were purchased from commercial vendors and initially concentrated to ~2.5 mg/ml using Amicon Ultra Centrifugal Filters (100 kDa MWCO).
2. Azide or any other preservatives were removed, and the antibody was buffer-exchanged to phosphate buffered saline (PBS, pH 7.4) using Zeba spin columns (7000 MWCO).
3. The concentration of the antibody was adjusted and in a typical conjugation experiment 200 µg of antibody in 95 µl of PBS was used in the next step.
4. 200 µg antibody in 95 µl PBS was mixed with 7.5 eq of PEGylated SMCC (succinimidyl 4-(N-maleimidomethyl)cyclohexane-1-carboxylate) cross-linker (SM(PEG)$_2$) in 5 µl of DMF (dimethyl formamide). The solution was then incubated at 4 °C for 3 h.
5. Excess PEGylated SMCC cross-linker was removed from maleimide-activated antibodies using Zeba spin columns (7000 MWCO, eluent: PBS, pH 7.4).
6. In parallel, thiol-modified DNA oligos (20 nmole) were reduced using dithiothreitol (DTT, 100 mM) in 0.1 ml PBS (1 mM EDTA, pH 8.0) for 2 h at room temperature. The reduced DNA oligos were purified using NAP-5 columns (GE Healthcare). Deionized water was used as eluent.
7. The maleimide-activated antibodies were mixed with the reduced form of their respective DNA oligos (15 eq) in PBS solution. The reaction was allowed to proceed for 12 h at 4 °C.
8. DNA-antibody conjugates were purified and concentrated using Amicon Ultra Centrifugal Filters (100 kDa MWCO).

Supplementary Protocol 2. Characterization of DNA-conjugated antibodies

Matrix-assisted laser desorption/ionization time of flight (MALDI-TOF) mass spectrometry was used to verify successful conjugation of DNA to the antibody as well as to quantify the number of DNA conjugated to each antibody. The DNA-modified antibody (DNA-Ab, conc. of ~1 mg/ml) was transferred to Milli-Q water using Zeba spin columns (7000 MWCO). A matrix solution was prepared by dissolving sinapinic acid (1 mg) in acetonitrile (70 µl) and water with 0.1% trifluoroacetic acid (30 µl). 1 µl of the DNA-antibody solution was deposited onto the MALDI plate and then mixed with 1 µl of MALDI matrix. The plate was allowed to dry at room temperature for ~4-5 h. The MALDI-TOF mass data was collected using the AB SCIEX 4800 MALDI-TOF/TOF analyzer.

Supplementary Protocol 3. Nanobody-DNA conjugate preparation

<u>Preparation of TCO conjugated GFP nanobody</u>

1. GFP nanobody (250 µl, 1 mg/ml) was first buffer-exchanged to PBS (pH7.4) containing 10% 1 M NaHCO3 (v/v) using Zeba spin columns (7000 MWCO).
2. 25 eq TCO-NHS in 12.5 µl was added into the GFP nanobody solution.
3. The reaction was incubated at RT for 3 h.
4. Excess TCO-NHS was removed from nanobodies using Zeba spin columns (7000 MWCO, eluent: PBS, pH 7.4).

<u>Coupling of TCO modified GFP (TCO-GFP) with tetrazine (Tz) modified DNA</u>

1. TCO-GFP was incubated with 3 eq of tetrazine (Tz) modified DNA.
2. The reaction mixture was incubated at RT for 3 h.
3. Excess Tz modified DNA was removed from DNA conjugated nanobodies using Zeba spin columns (7000 MWCO, eluent: PBS, pH 7.4).

4. DNA modified nanobodies were further purified using Amicon Ultra Centrifugal Filter (10 kDa MWCO).

Supplementary Protocol 4. Phalloidin-DNA conjugate preparation

Preparation of TCO conjugated phalloidin

1. Phalloidin-amine (0.25 mg), bought from commercial sources, was first dissolved in anhydrous DMF.
2. 5 eq triethyl amine and 5 eq of TCO-NHS were added into the Phalloidin-amine solution.
3. The reaction was stirred at RT overnight.
4. After completion of the reaction, TCO-conjugated phalloidin was purified using HPLC.
5. The successful conjugation was verified using ESI mass spectrometry.

Preparation of tetrazine (Tz) conjugated DNA

1. Amine-modified DNA was dissolved in water and buffered using 1 M NaHCO3 (10% v/v).
2. 5× Tz-NHS was added into the amine-modified DNA solution.
3. The reaction mixture was incubated at RT for 3 h.
4. The excess Tz-NHS or its hydrolyzed product was removed from Tz-conjugated DNA oligos using NAP-5 column (GE Healthcare) using deionized water as eluent.
5. Tz-conjugated DNA was further purified using HPLC.
6. The successful conjugation was verified using MALDI mass spectrometry.

Coupling of TCO-Phalloidin with tetrazine (Tz) modified DNA

1. TCO-Phalloidin was incubated with tetrazine (Tz) modified DNA in a 1:1 ration.
2. The reaction mixture was incubated at RT for 3 h.
3. Phalloidin-conjugated DNA oligo was purified using NAP-5 column (GE Healthcare) using deionized water was used as eluent.
4. Phalloidin-conjugated DNA was further purified using HPLC.
5. The successful conjugation was verified using MALDI mass spectrometry.

Supplementary Protocol 5. Immunostaining protocol with only PFA.

1. 24 h before incubation, ~25,000 cells/well was plated in a Lab-Tek chamber.
2. Culture medium was removed and proceed to fixation.
3. Fixation for 10 min with 4% paraformaldehyde in PBS.
4. Washing with PBS (3×).
5. Permeabilization with 0.25% v/v Triton X-100 in PBS for 10 min.
6. Washing with PBS (3×).
7. Blocking for 2 h with 3% bovine serum albumin and 0.1% v/v Triton X-100 in PBS.
8. Staining for overnight at 4C with primary antibody (10 µg/ml) diluted in 3% bovine serum albumin and 0.1% v/v Triton X-100 in PBS.
9. Washing with PBS (3×) with 5 min incubation each time.
10. Incubation for 1 h with secondary antibodies (10 µg/ml) in 3% bovine serum albumin and 0.1% v/v Triton X-100 in PBS to a concentration.
11. Washing with PBS (3×) with 5 min incubation each time.
12. Proceed to DNA-PAINT imaging.

Supplementary Protocol 6. Immunostaining protocol with PFA+glutaraldehyde.

1. 24 h before incubation, ~25,000 cells/well was plated in a Lab-Tek chamber.
2. Culture media was removed and proceed to fixation.
3. Fixation for 10 min with 3% paraformaldehyde and 0.1% glutaraldehyde in PBS.
4. Washing with PBS (3×).
5. Reduction for 7 min with 0.1% sodium borohydride in PBS to reduce background fluorescence.
6. Washing with PBS (3×) with 5 min incubation each time.
7. Blocking for 2 h with 3% bovine serum albumin and 0.25% v/v Triton X-100 in PBS.
8. Staining for overnight at 4C with primary antibody (10 μg/ml) against tubulin diluted in 3% bovine serum albumin and 0.1% v/v Triton X-100 in PBS to a concentration of 10 μg/mL.
9. Washing with PBS (3×) with 5 min incubation each time.
10. Incubation for 1 h with secondary antibodies (10 μg/ml) at a concentration of ~5-10 μg/mL in 3% bovine serum albumin and 0.1% v/v Triton X-100 in PBS to a concentration.
11. Washing with PBS (3×) with 5 min incubation each time.
12. Proceed to DNA-PAINT imaging.

Supplementary Protocol 7. Immunostaining protocol with methanol.

1. 24 h before incubation, ~25,000 cells/well was plated in a Lab-Tek chamber.
2. Culture media was removed and proceed to fixation.
3. Fixation for 15 min with 100% methanol at -20°C.
4. Washing with PBS (3×) with 5 min incubation each time.
5. Blocking for 3 h with 3% bovine serum albumin.
6. Staining for overnight at 4 °C with primary antibody (10 μg/ml) against tubulin diluted in 3% bovine serum albumin and 0.1% v/v Triton X-100 in PBS to a concentration of 10 μg/mL.
7. Washing with PBS (3X) with 5 min incubation each time.
8. Incubation for 1 h with secondary antibodies (10 μg/ml) at a concentration of ~5-10 μg/mL in 3% bovine serum albumin and 0.1% v/v Triton X-100 in PBS to a concentration.
9. Washing with PBS (3×) with 5 min incubation each time.
10. Proceed to DNA-PAINT imaging.

Supplementary Table 1. DNA origami sequences

**Name**	**Sequences**
21[32]23[31]Cus2	TTTTCACTCAAAGGGCGAAAAACCATCACC
19[32]21[31]Cus2	GTCGACTTCGGCCAACGCGCGGGGTTTTTC
17[32]19[31]Cus1	TGCATCTTTCCCAGTCACGACGGCCTGCAG
15[32]17[31]Cus1	TAATCAGCGGATTGACCGTAATCGTAACCG
13[32]15[31]Cus1	AACGCAAAATCGATGAACGGTACCGGTTGA
11[32]13[31]Cus2	AACAGTTTTGTACCAAAAACATTTTATTTC

9[32]11[31]Cus2	TTTACCCCAACATGTTTTAAATTTCCATAT
7[32]9[31]Cus1	TTTAGGACAAATGCTTTAAACAATCAGGTC
5[32]7[31]Cus1	CATCAAGTAAAACGAACTAACGAGTTGAGA
3[32]5[31]Cus1	AATACGTTTGAAAGAGGACAGACTGACCTT
1[32]3[31]Cus2	AGGCTCCAGAGGCTTTGAGGACACGGGTAA
0[47]1[31]Cus2	AGAAAGGAACAACTAAAGGAATTCAAAAAAA
23[32]22[48]Cus2	CAAATCAAGTTTTTTGGGGTCGAAACGTGGA
22[47]20[48]Cus2	CTCCAACGCAGTGAGACGGGCAACCAGCTGCA
20[47]18[48]Cus1	TTAATGAACTAGAGGATCCCCGGGGGGTAACG
18[47]16[48]Cus1	CCAGGGTTGCCAGTTTGAGGGGACCCGTGGGA
16[47]14[48]Cus1	ACAAACGGAAAAGCCCCAAAAACACTGGAGCA
14[47]12[48]Cus2	AACAAGAGGGATAAAAATTTTTAGCATAAAGC
12[47]10[48]Cus2	TAAATCGGGATTCCCAATTCTGCGATATAATG
10[47]8[48]Cus1	CTGTAGCTTGACTATTATAGTCAGTTCATTGA
8[47]6[48]Cus1	ATCCCCCTATACCACATTCAACTAGAAAAATC
6[47]4[48]Cus1	TACGTTAAAGTAATCTTGACAAGAACCGAACT
4[47]2[48]Cus2	GACCAACTAATGCCACTACGAAGGGGGTAGCA
2[47]0[48]Cus2	ACGGCTACAAAAGGAGCCTTTAATGTGAGAAT
21[56]23[63]Cus1	AGCTGATTGCCCTTCAGAGTCCACTATTAAAGGGTGCCGT
18[63]20[56]Cus8	ATTAAGTTTACCGAGCTCGAATTCGGGAAACCTGTCGTGC
15[64]18[64]Cus1	GTATAAGCCAACCCGTCGGATTCTGACGACAGTATCGGCCGCAAGGCG
13[64]15[63]Cus1	TATATTTTGTCATTGCCTGAGAGTGGAAGATT
11[64]13[63]Cus1	GATTTAGTCAATAAAGCCTCAGAGAACCCTCA
9[64]11[63]Cus1	CGGATTGCAGAGCTTAATTGCTGAAACGAGTA
7[56]9[63]Cus1	ATGCAGATACATAACGGGAATCGTCATAAATAAAGCAAAG
4[63]6[56]Cus8	ATAAGGGAACCGGATATTCATTACGTCAGGACGTTGGGAA
1[64]4[64]Cus1	TTTATCAGGACAGCATCGGAACGACACCAACCTAAAACGAGGTCAATC
0[79]1[63]Cus1	ACAACTTTCAACAGTTTCAGCGGATGTATCGG
23[64]22[80]Cus1	AAAGCACTAAATCGGAACCCTAATCCAGTT
22[79]20[80]Cus1	TGGAACAACCGCCTGGCCCTGAGGCCCGCT
20[79]18[80]Cus1	TTCCAGTCGTAATCATGGTCATAAAAGGGG
18[79]16[80]Cus1	GATGTGCTTCAGGAAGATCGCACAATGTGA
16[79]14[80]Cus1	GCGAGTAAAAATATTTAAATTGTTACAAAG

14[79]12[80]Cus1	GCTATCAGAAATGCAATGCCTGAATTAGCA
12[79]10[80]Cus1	AAATTAAGTTGACCATTAGATACTTTTGCG
10[79]8[80]Cus1	GATGGCTTATCAAAAAGATTAAGAGCGTCC
8[79]6[80]Cus1	AATACTGCCCAAAAGGAATTACGTGGCTCA
6[79]4[80]Cus1	TTATACCACCAAATCAACGTAACGAACGAG
4[79]2[80]Cus1	GCGCAGACAAGAGGCAAAAGAATCCCTCAG
2[79]0[80]Cus1	CAGCGAAACTTGCTTTCGAGGTGTTGCTAA
21[96]23[95]Cus2	AGCAAGCGTAGGGTTGAGTGTTGTAGGGAGCC
19[96]21[95]Cus2	CTGTGTGATTGCGTTGCGCTCACTAGAGTTGC
17[96]19[95]Cus1	GCTTTCCGATTACGCCAGCTGGCGGCTGTTTC
15[96]17[95]Cus1	ATATTTTGGCTTTCATCAACATTATCCAGCCA
13[96]15[95]Cus1	TAGGTAAACTATTTTTGAGAGATCAAACGTTA
11[96]13[95]Cus2	AATGGTCAACAGGCAAGGCAAAGAGTAATGTG
9[96]11[95]Cus2	CGAAAGACTTTGATAAGAGGTCATATTTCGCA
7[96]9[95]Cus1	TAAGAGCAAATGTTTAGACTGGATAGGAAGCC
5[96]7[95]Cus1	TCATTCAGATGCGATTTTAAGAACAGGCATAG
3[96]5[95]Cus1	ACACTCATCCATGTTACTTAGCCGAAAGCTGC
1[96]3[95]Cus2	AAACAGCTTTTTGCGGGATCGTCAACACTAAA
0[111]1[95]Cus2	TAAATGAATTTTCTGTATGGGATTAATTTCTT
23[96]22[112]Cus2	CCCGATTTAGAGCTTGACGGGGAAAAAGAATA
22[111]20[112]Cus2	GCCCGAGAGTCCACGCTGGTTTGCAGCTAACT
20[111]18[112]Cus1	CACATTAAAATTGTTATCCGCTCATGCGGGCC
18[111]16[112]Cus1	TCTTCGCTGCACCGCTTCTGGTGCGGCCTTCC
16[111]14[112]Cus1	TGTAGCCATTAAAATTCGCATTAAATGCCGGA
14[111]12[112]Cus2	GAGGGTAGGATTCAAAAGGGTGAGACATCCAA
12[111]10[112]Cus2	TAAATCATATAACCTGTTTAGCTAACCTTTAA
10[111]8[112]Cus1	TTGCTCCTTTCAAATATCGCGTTTGAGGGGGT
8[111]6[112]Cus1	AATAGTAAACACTATCATAACCCTCATTGTGA
6[111]4[112]Cus1	ATTACCTTTGAATAAGGCTTGCCCAAATCCGC
4[111]2[112]Cus2	GACCTGCTCTTTGACCCCCAGCGAGGGAGTTA
2[111]0[112]Cus2	AAGGCCGCTGATACCGATAGTTGCGACGTTAG
21[120]23[127]Cus1	CCCAGCAGGCGAAAAATCCCTTATAAATCAAGCCGGCG
18[127]20[120]Cus8	GCGATCGGCAATTCCACACAACAGGTGCCTAATGAGTG

15[128]18[128]Cus1	TAAATCAAAATAATTCGCGTCTCGGAAACCAGGCAAAGGGAAGG
13[128]15[127]Cus1	GAGACAGCTAGCTGATAAATTAATTTTTGT
11[128]13[127]Cus1	TTTGGGGATAGTAGTAGCATTAAAAGGCCG
9[128]11[127]Cus1	GCTTCAATCAGGATTAGAGAGTTATTTTCA
7[120]9[127]Cus1	CGTTTACCAGACGACAAAGAAGTTTTGCCATAATTCGA
4[127]6[120]Cus8	TTGTGTCGTGACGAGAAACACCAAATTTCAACTTTAAT
1[128]4[128]Cus1	TGACAACTCGCTGAGGCTTGCATTATACCAAGCGCGATGATAAA
0[143]1[127]Cus1	TCTAAAGTTTTGTCGTCTTTCCAGCCGACAA
21[160]22[144]Cus1	TCAATATCGAACCTCAAATATCAATTCCGAAA
19[160]20[144]Cus1	GCAATTCACATATTCCTGATTATCAAAGTGTA
17[160]18[144]Cus1	AGAAAACAAAGAAGATGATGAAACAGGCTGCG
15[160]16[144]Cus1	ATCGCAAGTATGTAAATGCTGATGATAGGAAC
13[160]14[144]Cus1	GTAATAAGTTAGGCAGAGGCATTTATGATATT
11[160]12[144]Cus1	CCAATAGCTCATCGTAGGAATCATGGCATCAA
9[160]10[144]Cus1	AGAGAGAAAAAAATGAAAATAGCAAGCAAACT
7[160]8[144]Cus1	TTATTACGAAGAACTGGCATGATTGCGAGAGG
5[160]6[144]Cus1	GCAAGGCCTCACCAGTAGCACCATGGGCTTGA
3[160]4[144]Cus1	TTGACAGGCCACCACCAGAGCCGCGATTTGTA
1[160]2[144]Cus1	TTAGGATTGGCTGAGACTCCTCAATAACCGAT
0[175]0[144]Cus1	TCCACAGACAGCCCTCATAGTTAGCGTAACGA
23[128]23[159]Cus1	AACGTGGCGAGAAAGGAAGGGAAACCAGTAA
22[143]21[159]Cus1	TCGGCAAATCCTGTTTGATGGTGGACCCTCAA
20[143]19[159]Cus1	AAGCCTGGTACGAGCCGGAAGCATAGATGATG
18[143]17[159]Cus1	CAACTGTTGCGCCATTCGCCATTCAAACATCA
16[143]15[159]Cus1	GCCATCAAGCTCATTTTTTAACCACAAATCCA
14[143]13[159]Cus1	CAACCGTTTCAAATCACCATCAATTCGAGCCA
12[143]11[159]Cus1	TTCTACTACGCGAGCTGAAAAGGTTACCGCGC
10[143]9[159]Cus1	CCAACAGGAGCGAACCAGACCGGAGCCTTTAC
8[143]7[159]Cus1	CTTTTGCAGATAAAAACCAAAATAAAGACTCC
6[143]5[159]Cus1	GATGGTTTGAACGAGTAGTAAATTTACCATTA
4[143]3[159]Cus1	TCATCGCCAACAAAGTACAACGGACGCCAGCA
2[143]1[159]Cus1	ATATTCGGAACCATCGCCCACGCAGAGAAGGA
23[160]22[176]Cus1	TAAAAGGGACATTCTGGCCAACAAAGCATC

22[175]20[176]Cus1	ACCTTGCTTGGTCAGTTGGCAAAGAGCGGA
20[175]18[176]Cus1	ATTATCATTCAATATAATCCTGACAATTAC
18[175]16[176]Cus1	CTGAGCAAAAATTAATTACATTTTGGGTTA
16[175]14[176]Cus1	TATAACTAACAAAGAACGCGAGAACGCCAA
14[175]12[176]Cus1	CATGTAATAGAATATAAAGTACCAAGCCGT
12[175]10[176]Cus1	TTTTATTTAAGCAAATCAGATATTTTTTGT
10[175]8[176]Cus1	TTAACGTCTAACATAAAAACAGGTAACGGA
8[175]6[176]Cus1	ATACCCAACAGTATGTTAGCAAATTAGAGC
6[175]4[176]Cus1	CAGCAAAAGGAAACGTCACCAATGAGCCGC
4[175]2[176]Cus1	CACCAGAAAGGTTGAGGCAGGTCATGAAAG
2[175]0[176]Cus1	TATTAAGAAGCGGGGTTTTGCTCGTAGCAT
21[184]23[191]Cus1	TCAACAGTTGAAAGGAGCAAATGAAAAATCTAGAGATAGA
18[191]20[184]Cus8	ATTCATTTTTGTTTGGATTATACTAAGAAACCACCAGAAG
15[192]18[192]Cus1	TCAAATATAACCTCCGGCTTAGGTAACAATTTCATTTGAAGGCGAATT
13[192]15[191]Cus1	GTAAAGTAATCGCCATATTTAACAAAACTTTT
11[192]13[191]Cus1	TATCCGGTCTCATCGAGAACAAGCGACAAAAG
9[192]11[191]Cus1	TTAGACGGCCAAATAAGAAACGATAGAAGGCT
7[184]9[191]Cus1	CGTAGAAAATACATACCGAGGAAACGCAATAAGAAGCGCA
4[191]6[184]Cus8	CACCCTCAGAAACCATCGATAGCATTGAGCCATTTGGGAA
1[192]4[192]Cus1	GCGGATAACCTATTATTCTGAAACAGACGATTGGCCTTGAAGAGCCAC
0[207]1[191]Cus4	TCACCAGTACAAACTACAACGCCTAGTACCAG
23[192]22[208]Cus1	ACCCTTCTGACCTGAAAGCGTAAGACGCTGAG
22[207]20[208]Cus1	AGCCAGCAATTGAGGAAGGTTATCATCATTTT
20[207]18[208]Cus1	GCGGAACATCTGAATAATGGAAGGTACAAAAT
18[207]16[208]Cus1	CGCGCAGATTACCTTTTTTAATGGGAGAGACT
16[207]14[208]Cus1	ACCTTTTTATTTTAGTTAATTTCATAGGGCTT
14[207]12[208]Cus4	AATTGAGAATTCTGTCCAGACGACTAAACCAA
12[207]10[208]Cus4	GTACCGCAATTCTAAGAACGCGAGTATTATTT
10[207]8[208]Cus1	ATCCCAATGAGAATTAACTGAACAGTTACCAG
8[207]6[208]Cus1	AAGGAAACATAAAGGTGGCAACATTATCACCG
6[207]4[208]Cus1	TCACCGACGCACCGTAATCAGTAGCAGAACCG
4[207]2[208]Cus1	CCACCCTCTATTCACAAACAAATACCTGCCTA
2[207]0[208]Cus1	TTTCGGAAGTGCCGTCGAGAGGGTGAGTTTCG

21[224]23[223]Cus1	CTTTAGGGCCTGCAACAGTGCCAATACGTG
19[224]21[223]Cus1	CTACCATAGTTTGAGTAACATTTAAAATAT
17[224]19[223]Cus1	CATAAATCTTTGAATACCAAGTGTTAGAAC
15[224]17[223]Cus1	CCTAAATCAAAATCATAGGTCTAAACAGTA
13[224]15[223]Cus1	ACAACATGCCAACGCTCAACAGTCTTCTGA
11[224]13[223]Cus4	GCGAACCTCCAAGAACGGGTATGACAATAA
9[224]11[223]Cus1	AAAGTCACAAAATAAACAGCCAGCGTTTTA
7[224]9[223]Cus1	AACGCAAAGATAGCCGAACAAACCCTGAAC
5[224]7[223]Cus1	TCAAGTTTCATTAAAGGTGAATATAAAAGA
3[224]5[223]Cus1	TTAAAGCCAGAGCCGCCACCCTCGACAGAA
1[224]3[223]Cus1	GTATAGCAAACAGTTAATGCCCAATCCTCA
0[239]1[223]Cus4	AGGAACCCATGTACCGTAACACTTGATATAA
23[224]22[240]Cus1	GCACAGACAATATTTTTGAATGGGGTCAGTA
22[239]20[240]Cus1	TTAACACCAGCACTAACAACTAATCGTTATTA
20[239]18[240]Cus1	ATTTTAAAATCAAAATTATTTGCACGGATTCG
18[239]16[240]Cus1	CCTGATTGCAATATATGTGAGTGATCAATAGT
16[239]14[240]Cus1	GAATTTATTTAATGGTTTGAAATATTCTTACC
14[239]12[240]Cus1	AGTATAAAGTTCAGCTAATGCAGATGTCTTTC
12[239]10[240]Cus1	CTTATCATTCCCGACTTGCGGGAGCCTAATTT
10[239]8[240]Cus1	GCCAGTTAGAGGGTAATTGAGCGCTTTAAGAA
8[239]6[240]Cus1	AAGTAAGCAGACACCACGGAATAATATTGACG
6[239]4[240]Cus1	GAAATTATTGCCTTTAGCGTCAGACCGGAACC
4[239]2[240]Cus1	GCCTCCCTCAGAATGGAAAGCGCAGTAACAGT
2[239]0[240]Cus1	GCCCGTATCCGGAATAGGTGTATCAGCCCAAT
21[248]23[255]Cus1	AGATTAGAGCCGTCAAAAAACAGAGGTGAGGCCTATTAGT
18[255]20[248]Cus8	AACAATAACGTAAAACAGAAATAAAAATCCTTTGCCCGAA
15[256]18[256]Cus1	GTGATAAAAAGACGCTGAGAAGAGATAACCTTGCTTCTGTTCGGGAGA
13[256]15[255]Cus1	GTTTATCAATATGCGTTATACAAACCGACCGT
11[256]13[255]Cus1	GCCTTAAACCAATCAATAATCGGCACGCGCCT
9[256]11[255]Cus1	GAGAGATAGAGCGTCTTTCCAGAGGTTTTGAA
7[248]9[255]Cus1	GTTTATTTTGTCACAATCTTACCGAAGCCCTTTAATATCA
4[255]6[248]Cus8	AGCCACCACTGTAGCGCGTTTTCAAGGGAGGGAAGGTAAA
1[256]4[256]Cus1	CAGGAGGTGGGGTCAGTGCCTTGAGTCTCTGAATTTACCGGGAACCAG

0[271]1[255]Cus4	CCACCCTCATTTTCAGGGATAGCAACCGTACT
23[256]22[272]Cus4	CTTTAATGCGCGAACTGATAGCCCCACCAG
22[271]20[272]Cus1	CAGAAGATTAGATAATACATTTGTCGACAA
20[271]18[272]Cus4	CTCGTATTAGAAATTGCGTAGATACAGTAC
18[271]16[272]Cus1	CTTTTACAAAATCGTCGCTATTAGCGATAG
16[271]14[272]Cus4	CTTAGATTTAAGGCGTTAAATAAAGCCTGT
14[271]12[272]Cus1	TTAGTATCACAATAGATAAGTCCACGAGCA
12[271]10[272]Cus4	TGTAGAAATCAAGATTAGTTGCTCTTACCA
10[271]8[272]Cus1	ACGCTAACACCCACAAGAATTGAAAATAGC
8[271]6[272]Cus4	AATAGCTATCAATAGAAAATTCAACATTCA
6[271]4[272]Cus1	ACCGATTGTCGGCATTTTCGGTCATAATCA
4[271]2[272]Cus4	AAATCACCTTCCAGTAAGCGTCAGTAATAA
2[271]0[272]Cus1	GTTTTAACTTAGTACCGCCACCCAGAGCCA

Modifications:

- Cus1: unmodified structure staples
- Cus2: 6-bit barcode staples for crosstalk check
- Cus4: Mirrored “F” staples for crosstalk check
- Cus8: 5’-biotinylated staples for surface attachment

Supplementary Table 2. 52 orthogonal DNA-PAINT imager and corresponding docking sequences.

Name	imager	docking
P1	CTAGATGTAT-dye	TTATACATCTA
P2	TATGTAGATC-dye	TTGATCTACAT
P3	GTAATGAAGA-dye	TTTCTTCATTA
P4	GTAGATTCAT-dye	TTATGAATCTA
P5	CATACATTGA-dye	TTTCAATGTAT
P6	CTTTACCTAA-dye	TTTTAGGTAAA
P7	GTACTCAATT-dye	TTAATTGAGTA
P8	CCATTAACAT-dye	TTATGTTAATG
P9	CATCCTAATT-dye	TTAATTAGGAT
P10	GATCCATTAT-dye	TTATAATGGAT
P11	CACCTTATTA-dye	TTTAATAAGGT
P12	GCTCTAACTA-dye	TTTAGTTAGAG

P13	CCTTCTCTAT-dye	TTATAGAGAAG
P14	GTATCATCAA-dye	TTTTGATGATA
P15	CAACAAACTA-dye	TTTAGTTTGTT
P16	CAATTAAACG-dye	TTCGTTTAATT
P17	CAATTTTAGG-dye	TTCCTAAAATT
P18	CACACTTTAT-dye	TTATAAAGTGT
P19	CAGATCATAT-dye	TTATATGATCT
P20	CAGCTTAATA-dye	TTTATTAAGCT
P21	CATTCTATGT-dye	TTACATAGAAT
P22	CATTTCACAT-dye	TTATGTGAAAT
P23	CCAAAGTATT-dye	TTAATACTTTG
P24	CCATGATTAT-dye	TTATAATCATG
P25	CCTGTTTTAA-dye	TTTTAAAACAG
P26	CGAACTTTTT-dye	TTAAAAAGTTC
P27	CGAGTTATAT-dye	TTATATAACTC
P28	CGGTATAATT-dye	TTAATTATACC
P29	CGTCAATATA-dye	TTTATATTGAC
P30	CTATGCTTTA-dye	TTTAAAGCATA
P31	CTGTAAATTC-dye	TTGAATTTACA
P32	CTGTTGAAAA-dye	TTTTTTCAACA
P33	CTTAGTTGAT-dye	TTATCAACTAA
P34	CTTATAGTTC-dye	TTGAACTATAA
P35	CTTCTGTTAT-dye	TTATAACAGAA
P36	CTTTGAGATT-dye	TTAATCTCAAA
P37	GACACTAAAT-dye	TTATTTAGTGT
P38	GAGAACATAA-dye	TTTTATGTTCT
P39	GATAAGATAG-dye	TTCTATCTTAT
P40	GATACACATA-dye	TTTATGTGTAT
P41	GATTTATCCA-dye	TTTGGATAAAT
P42	GCAAGATTAA-dye	TTTTAATCTTG
P43	GCATTCAAAA-dye	TTTTTTGAATG
P44	GCTTTTCTTT-dye	TTAAAGAAAAG
P45	GGTTTTTATG-dye	TTCATAAAAAC

P46	GTATATCACA-dye	TTTGTGATATA
P47	GTATGACTTT-dye	TTAAAGTCATA
P48	GTCGATTTTT-dye	TTAAAAATCGA
P49	GTGTACTATT-dye	TTAATAGTACA
P50	GTTAAGGAAA-dye	TTTTTCCTTAA
P51	GTTTACGATT-dye	TTAATCGTAAA
P52	GTTTCGTATA-dye	TTTATACGAAA

Supplementary Table 3. Primary antibodies used in indirect immunostaining multiplexing

Target	Antibody commercial source	Species
Tubulin (alpha)	Thermo-Scientific (MA1-80017)	Rat
Nuclear Pore Complex	abcam (ab24609)	Mouse
Mitochondria (Tom20)	Santa Cruz (sc-11415)	Rabbit
EGFR	ImClone Systems (Cetuximab)	Human
Paxillin	R&D systems (AF4259)	Sheep
Vimentin	abcam (ab24525)	Chicken
Pan Cytokeratin	Acris Antibodies (BP5069)	Guinea pig

Supplementary Table 4. Secondary antibodies used in indirect immunostaining multiplexing

Target	Host	Specification and commercial source
Rat	Donkey	Donkey Anti-Rat IgG (H+L)<br>(min X Bov, Ck, Gt, GP, SyHms, Hrs, Hu, Ms, Rb, Shp Sr Prot)<br>Jackson ImmunoResearch Laboratories, INC. (712-005-153)
Mouse	Donkey	Donkey Anti-Mouse IgG (H+L)<br>(min X Bov, Ck, Gt, GP, Sy Hms, Hrs, Hu, Rb, Rat, Shp SrProt)<br>Jackson ImmunoResearch Laboratories, INC. (715-005-151)
Rabbit	Donkey	Donkey Anti-Rabbit IgG (H+L)<br>(min X Bov, Ck, Gt, GP, Sy Hms, Hrs, Hu, Ms, Rat, Shp Sr Prot)<br>Jackson ImmunoResearch Laboratories, INC. (711-005-152)
Human	Donkey	Donkey Anti-Human IgG (H+L)<br>(min X Bov, Ck, Gt, GP, Sy Hms, Hrs, Ms, Rb, Rat, Shp Sr Prot)<br>Jackson ImmunoResearch Laboratories, INC. (709-005-149)
Sheep	Donkey	Donkey Anti-Sheep IgG (H+L)<br>(min X Ck, GP, Sy Hms, Hrs, Hu, Ms, Rb, Rat Sr Prot)<br>Jackson ImmunoResearch Laboratories, INC. (713-005-147)
Chicken	Donkey	Donkey Anti-Chicken IgY (IgG) (H+L)<br>(min X Bov, Gt, GP, Sy Hms, Hrs, Hu, Ms, Rb, Rat, Shp Sr Prot)<br>Jackson ImmunoResearch Laboratories, INC. (703-005-155)
Guinea pig	Donkey	Donkey Anti-Guinea Pig IgG (H+L)

		(min X Bov, Ck, Gt, Sy Hms, Hrs, Hu, Ms, Rb, Rat, Shp Sr Prot) Jackson ImmunoResearch Laboratories, INC. (706-005-148)

Supplementary Table 5. DNA-barcoded labeling agents used in indirect immunostaining multiplexing

Target	Labeling protocol	Docking Strand	Imager strand
Actin	Phalloidin	Phalloidin-P1	P1*-Atto655 dye
Alpha Tubulin	Primary and secondary antibody	Donkey-anti-rat-P2	P2*-Atto655 dye
NPC	Primary and secondary antibody	Donkey-anti-mouse-P3	P3*-Atto655 dye
Tom20	Primary and secondary antibody	Donkey-anti-rabbit-P4	P4*-Atto655 dye
EGFR	Primary and secondary antibody	Donkey-anti-human-P5	P5*-Atto655 dye
Paxillin	Primary and secondary antibody	Donkey-anti-sheep-P6	P6*-Atto655 dye
Vimentin	Primary and secondary antibody	Donkey-anti-chicken-P7	P7*-Atto655 dye
Cytokeratin	Primary and secondary antibody	Donkey-anti-guinea pig-P9	P9*-Atto655 dye

Supplementary Table 6. Primary antibodies used in direct immunostaining multiplexing

Target	Antibody commercial source	Species
Paxillin	R&D systems (AF4259)	Sheep
Ki-67	Biolegend (350502)	Mouse
Acetylated Tubulin	Sigma-Aldrich (T7451)	Mouse
Mitochondria (Tom20)	Santa Cruz (sc-11415)	Rabbit
Nuclear Pore Complex (NUP-98)	abcam (ab50610)	Rat
Lamin (Lamin B1)	abcam (ab16048)	Rabbit
Clathrin (Clathrin heavy chain)	Thermo Scientific (MA1-065)	Mouse
Golgi (Golgin-97)	Thermo Scientific (A-21270)	Mouse

Supplementary Table 7. DNA-barcoded labeling agents used in direct immunostaining multiplexing

Target	Labeling protocol	Docking Strand	Imager strand
Actin	Phalloidin	Phalloidin-P1	P1*-Atto655 dye
Paxillin	Primary antibody	Paxillin antibody-P38	P38*-Atto655 dye
Ki-67	Primary antibody	Ki-67 antibody-P10	P10*-Atto655 dye
Acetylated tubulin	Primary antibody	Acetylated tubulin antibody-P29	P29*-Atto655 dye
Tom-20	Primary antibody	Tom-20 antibody-P8	P8*-Atto655 dye
Nup98	Primary antibody	NUP-98 antibody-P9	P9*-Atto655 dye

Lamin B1	Primary antibody	Lamin B1 antibody-P39	P39*-Atto655 dye
Clathrin heavy chain	Primary antibody	Clathrin heavy chain-P13	P13*-Atto655 dye
Golgin-97	Primary antibody	Golgin-97 antibody-P40	P40*-Atto655 dye

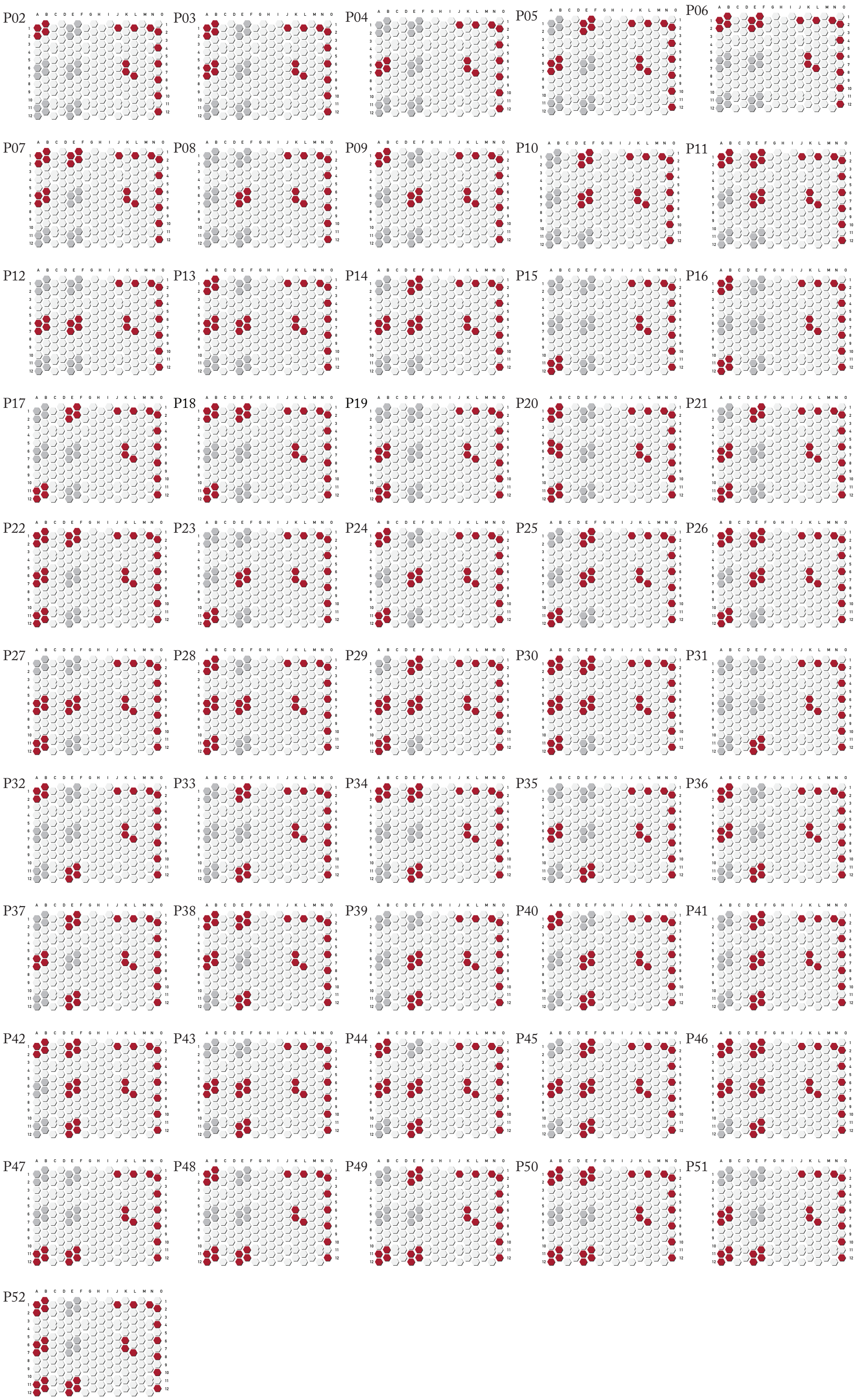

Supplementary Figure 1 | 51 barcoded DNA origami structures. Each hexagon represents a position for a staple strand extension. Each origami contains a unique 6-bit barcode addressable with imager sequence P1 (left side), and single-stranded extensions that will act as docking sites for the imager to be tested (P2 – P52). Together, these extensions form a mirrored “F” shape (right side).

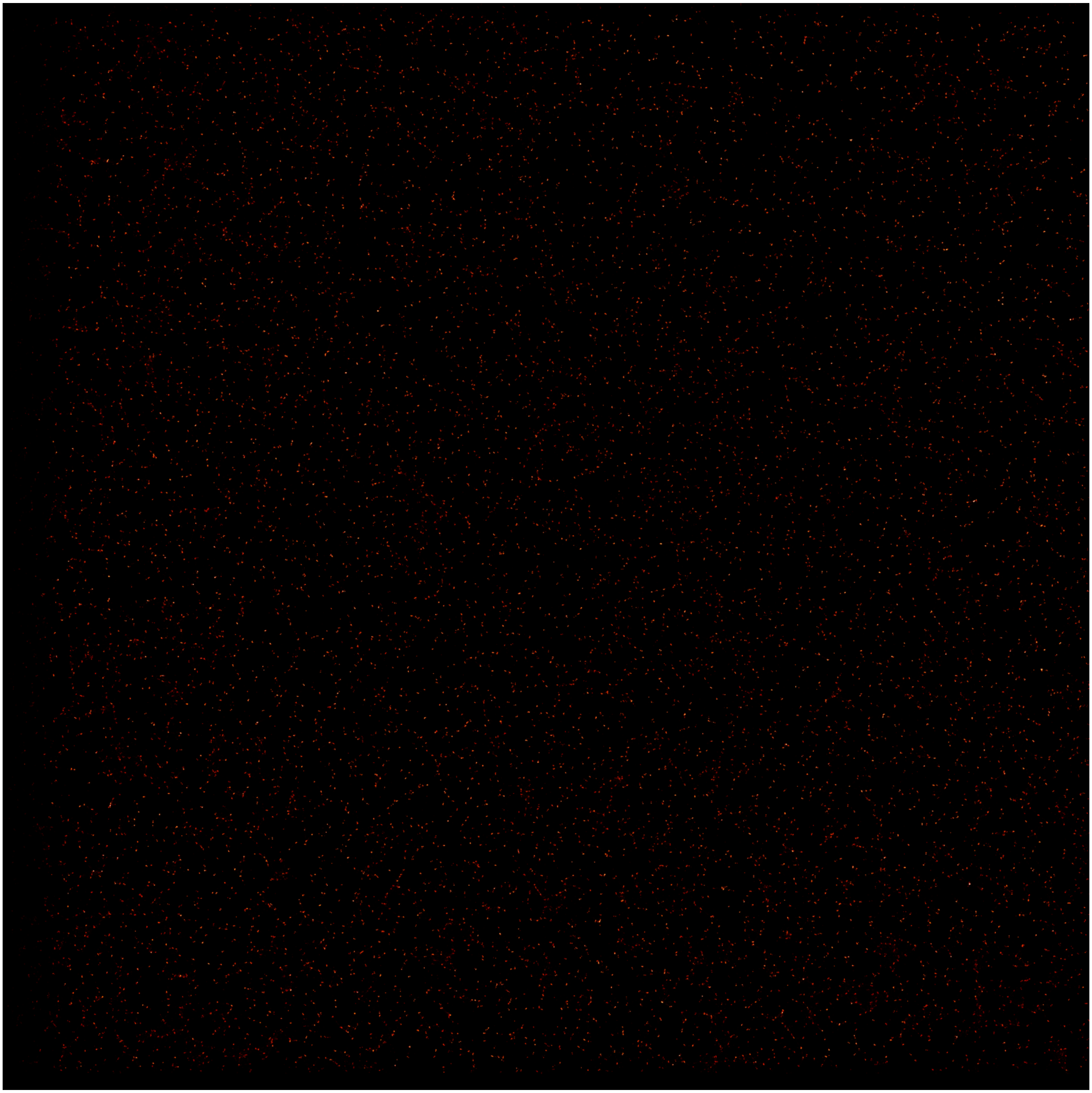

Supplementary Figure 2. Overview image of crosstalk experiment for imager sequence P2. Image size 40.96 µm.

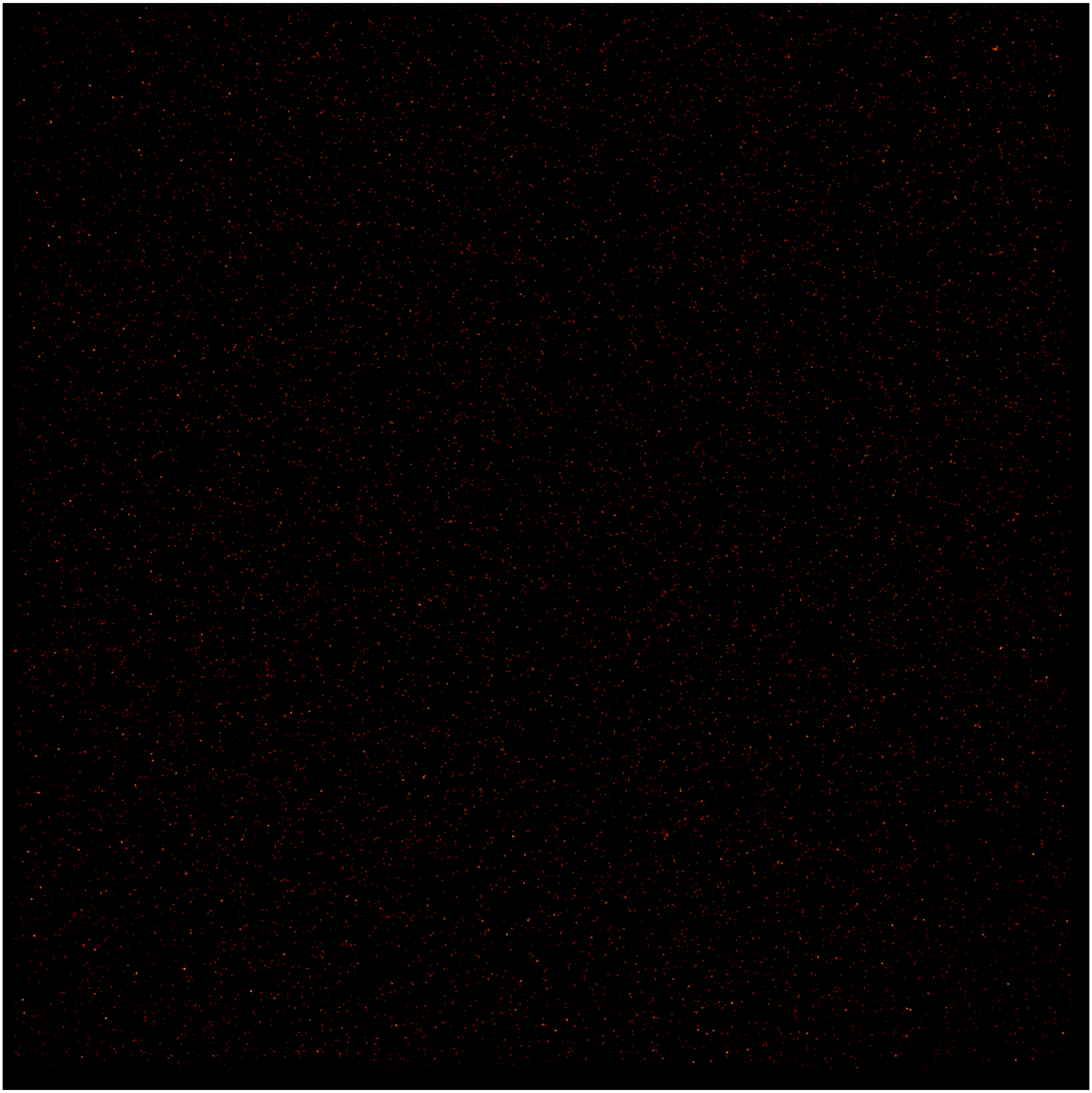

Supplementary Figure 3. Overview image of crosstalk experiment for imager sequence P3. Image size 40.96 µm.

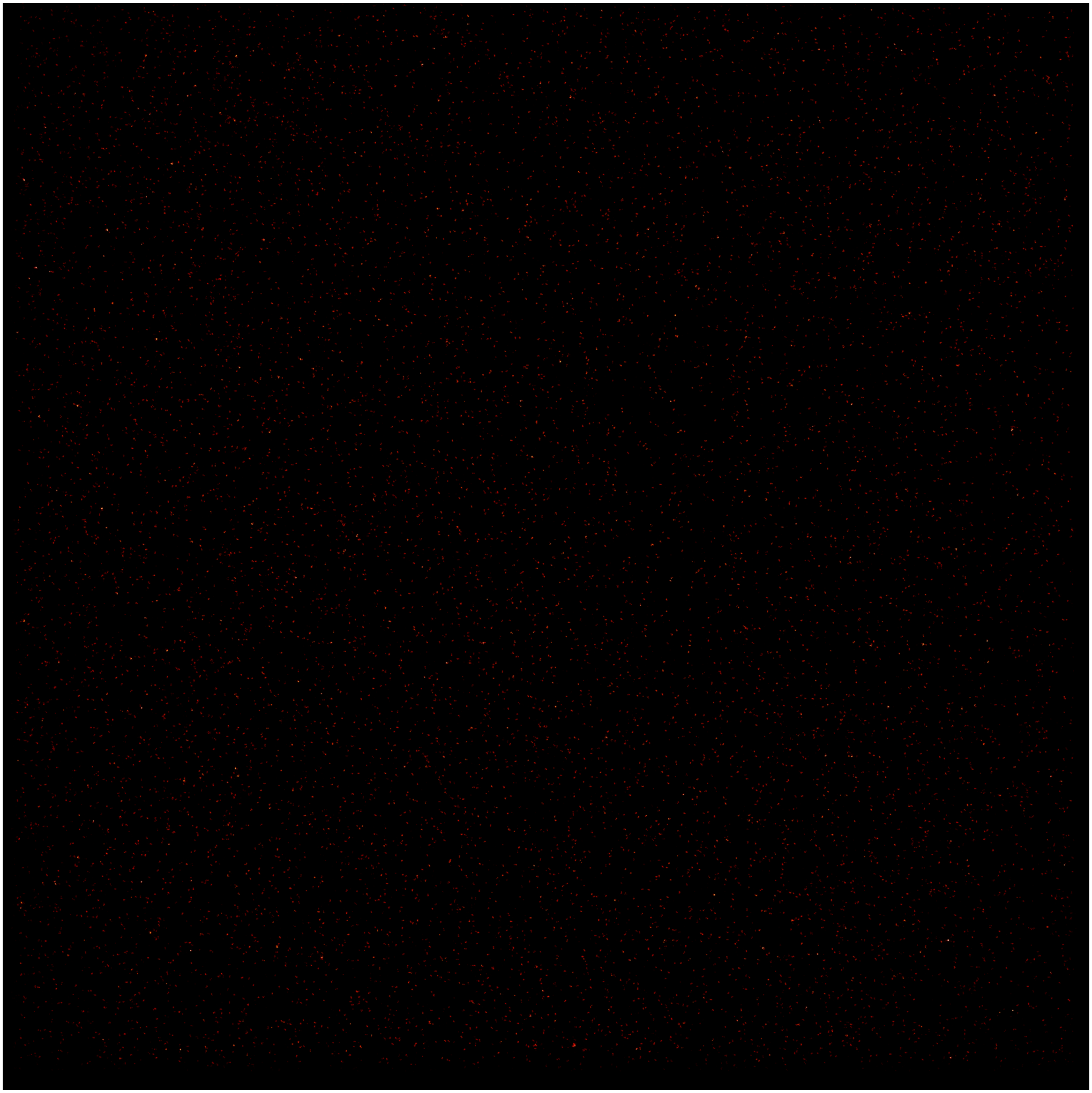

Supplementary Figure 4. Overview image of crosstalk experiment for imager sequence P4. Image size 40.96 µm.

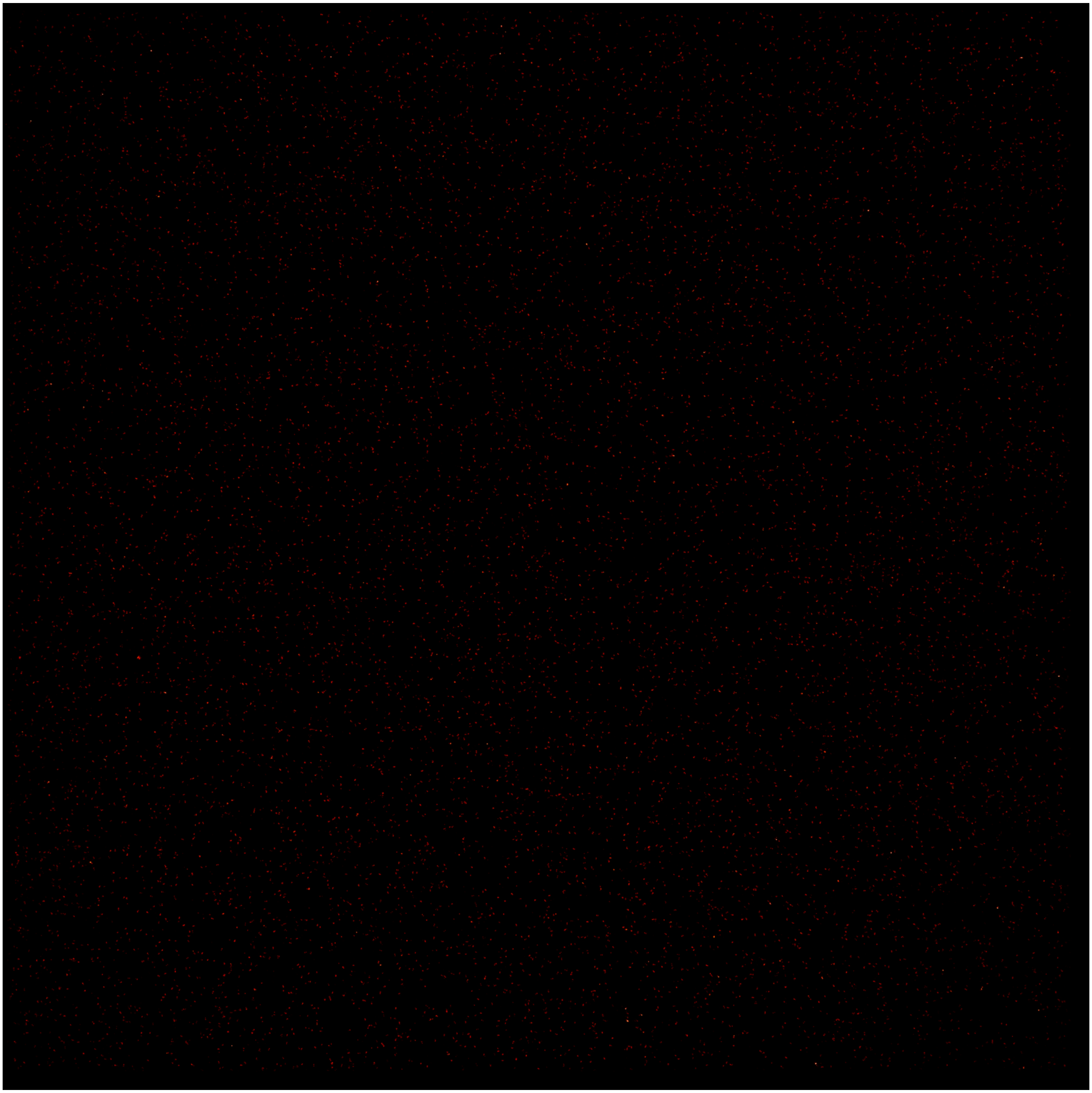

Supplementary Figure 5. Overview image of crosstalk experiment for imager sequence P5. Image size 40.96 µm.

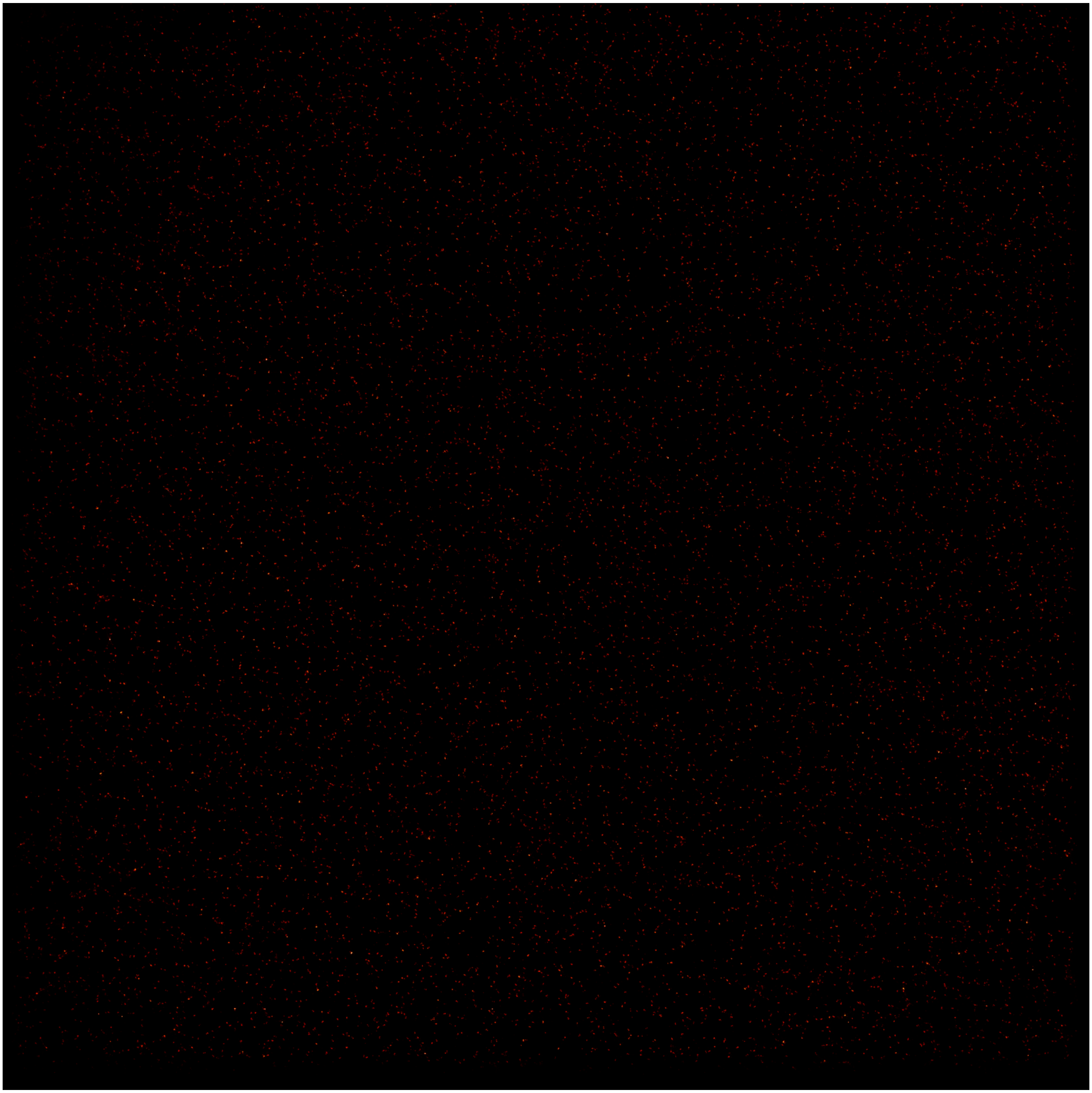

Supplementary Figure 6. Overview image of crosstalk experiment for imager sequence P6. Image size 40.96 μm.

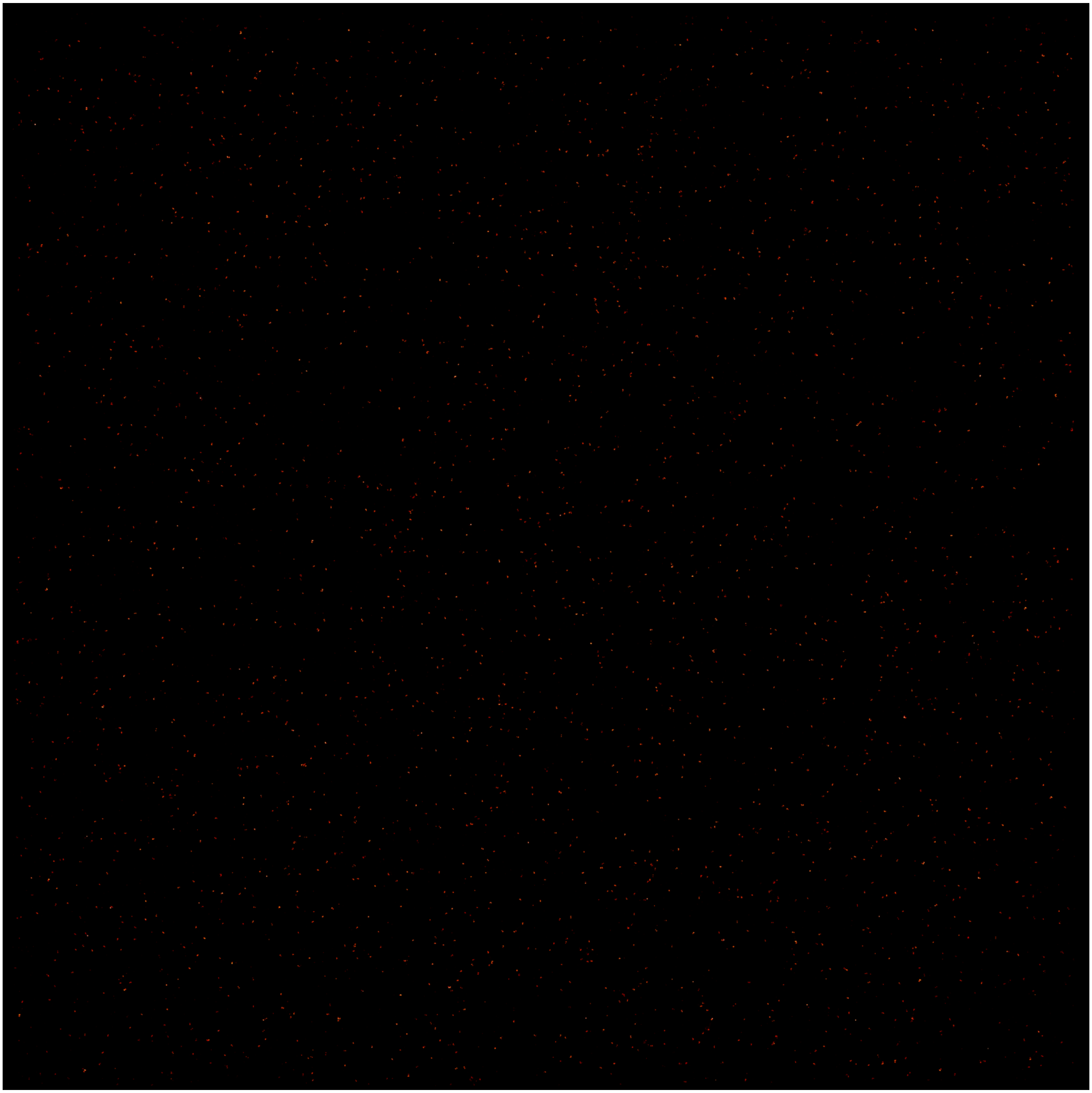

Supplementary Figure 7. Overview image of crosstalk experiment for imager sequence P7. Image size 40.96 µm.

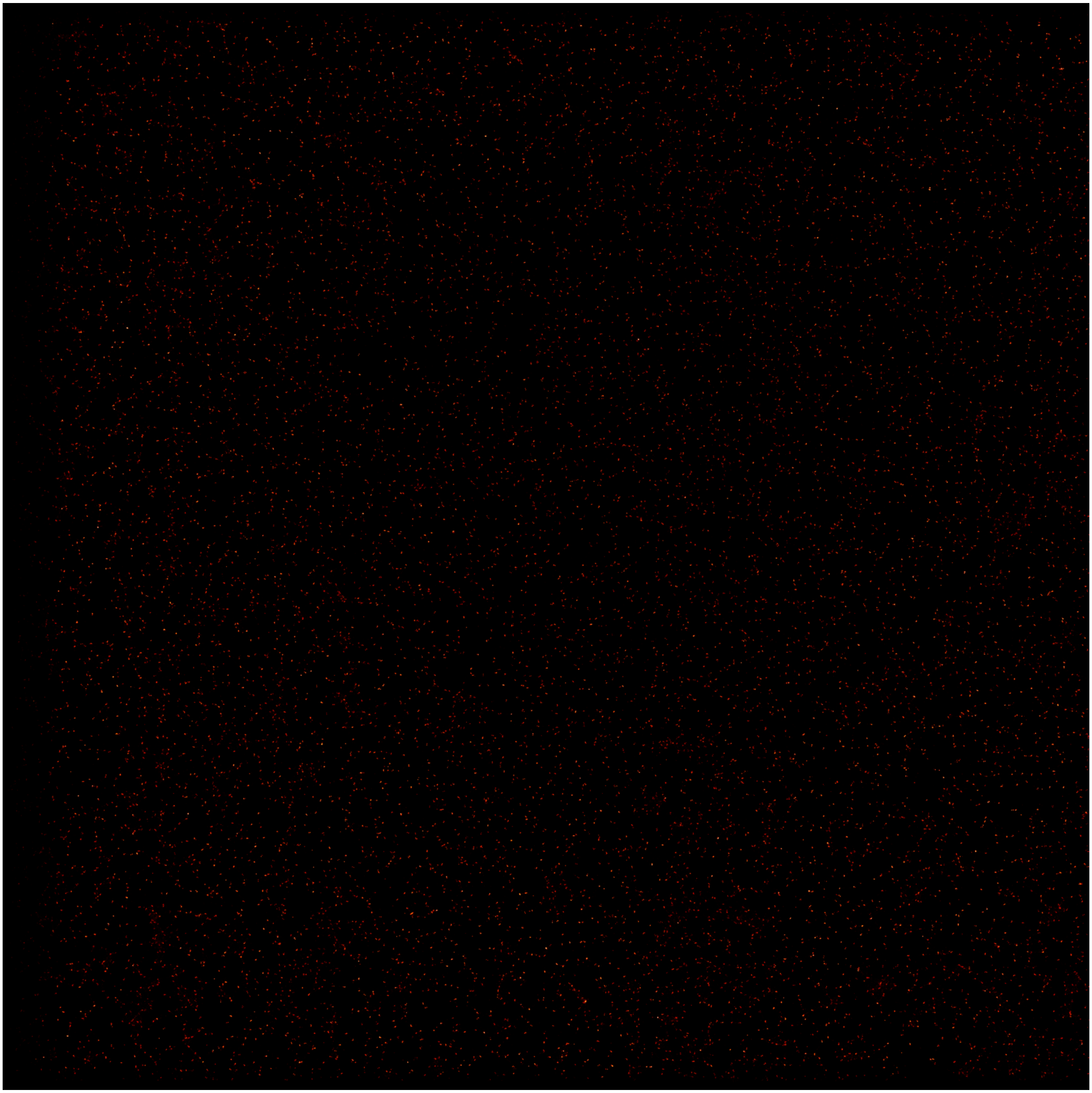

Supplementary Figure 8. Overview image of crosstalk experiment for imager sequence P8. Image size 40.96 µm.

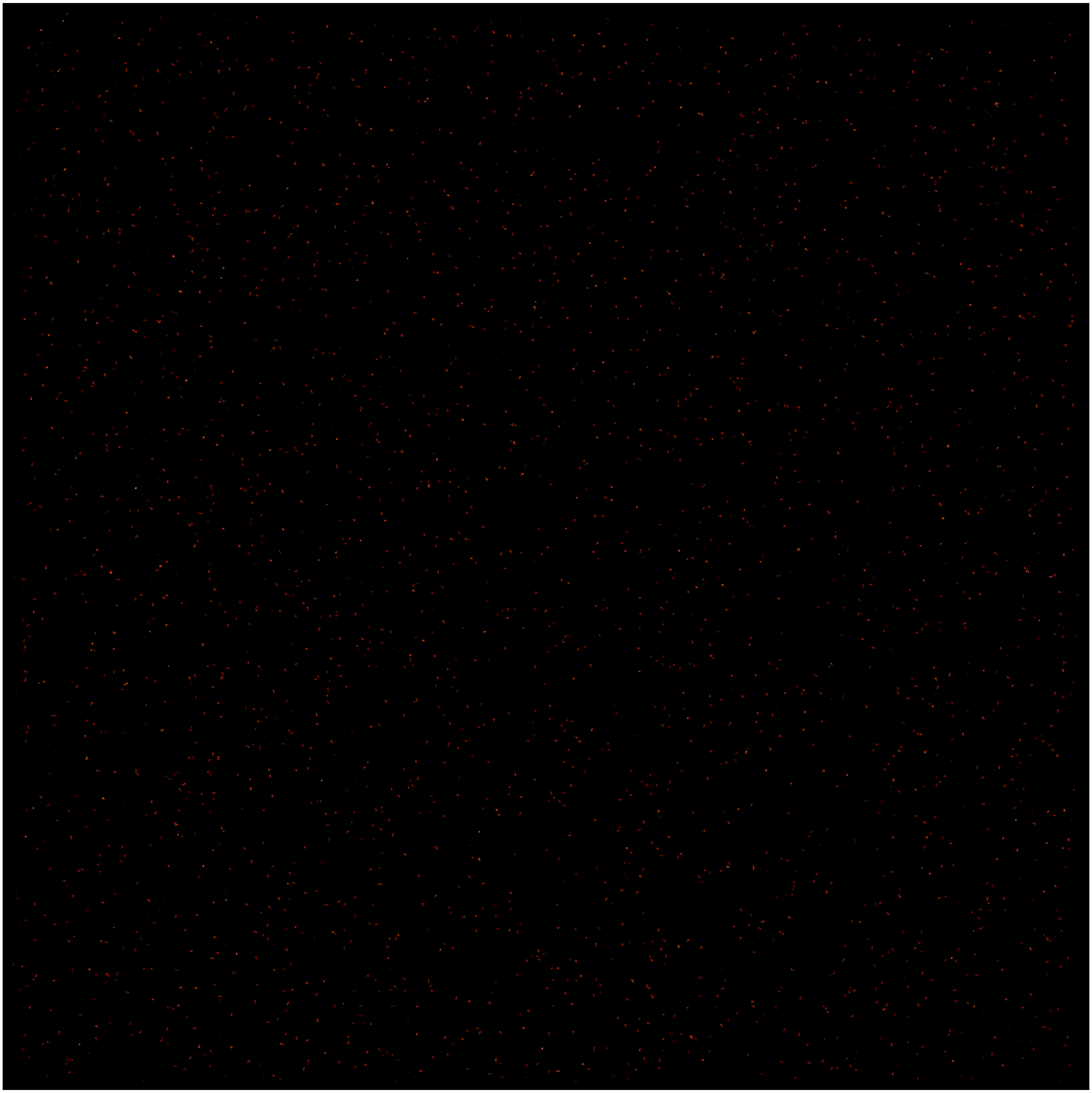

Supplementary Figure 9. Overview image of crosstalk experiment for imager sequence P9. Image size 40.96 µm.

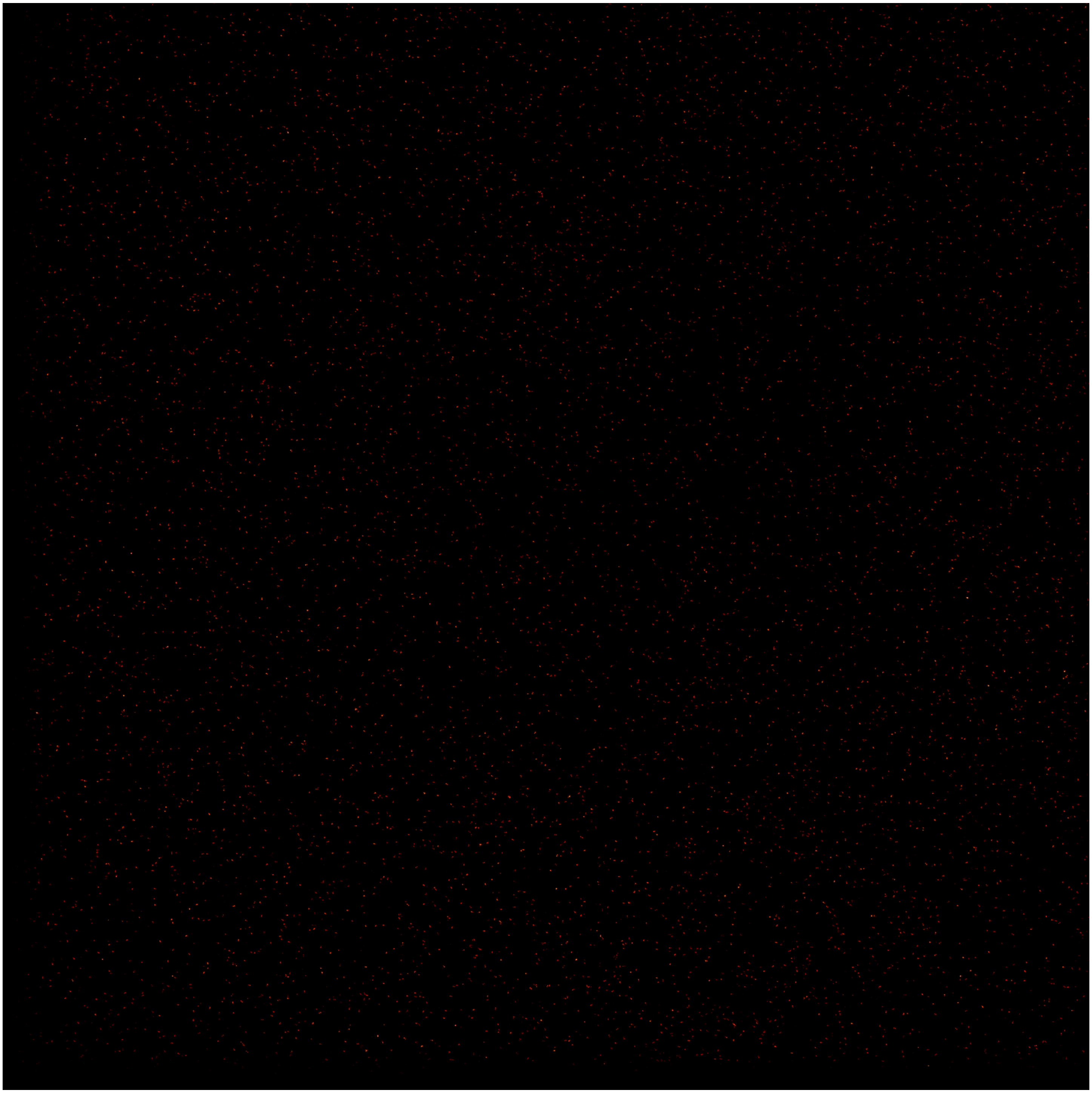

Supplementary Figure 10. Overview image of crosstalk experiment for imager sequence P10. Image size 40.96 µm.

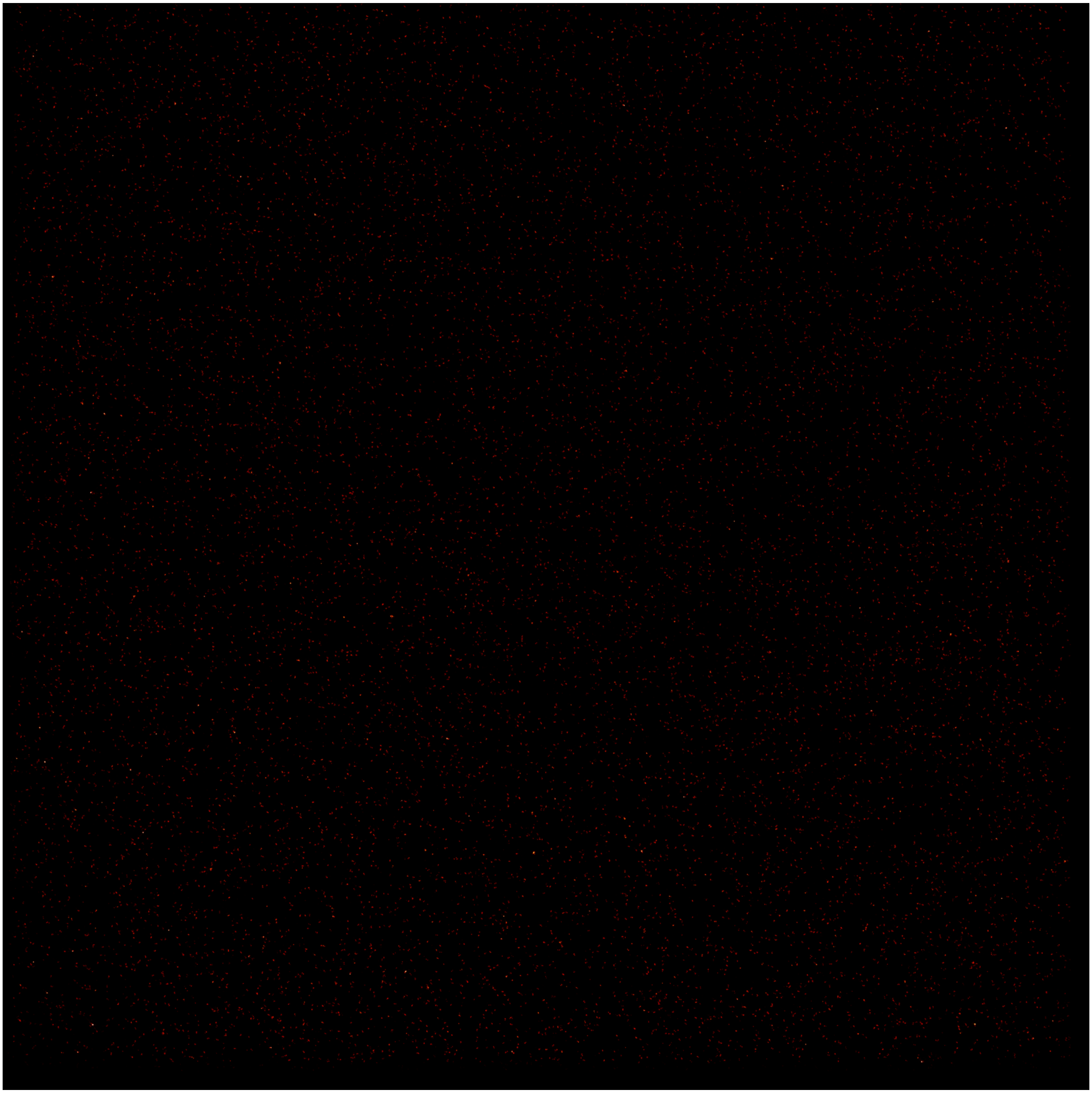

Supplementary Figure 11. Overview image of crosstalk experiment for imager sequence P11. Image size 40.96 µm.

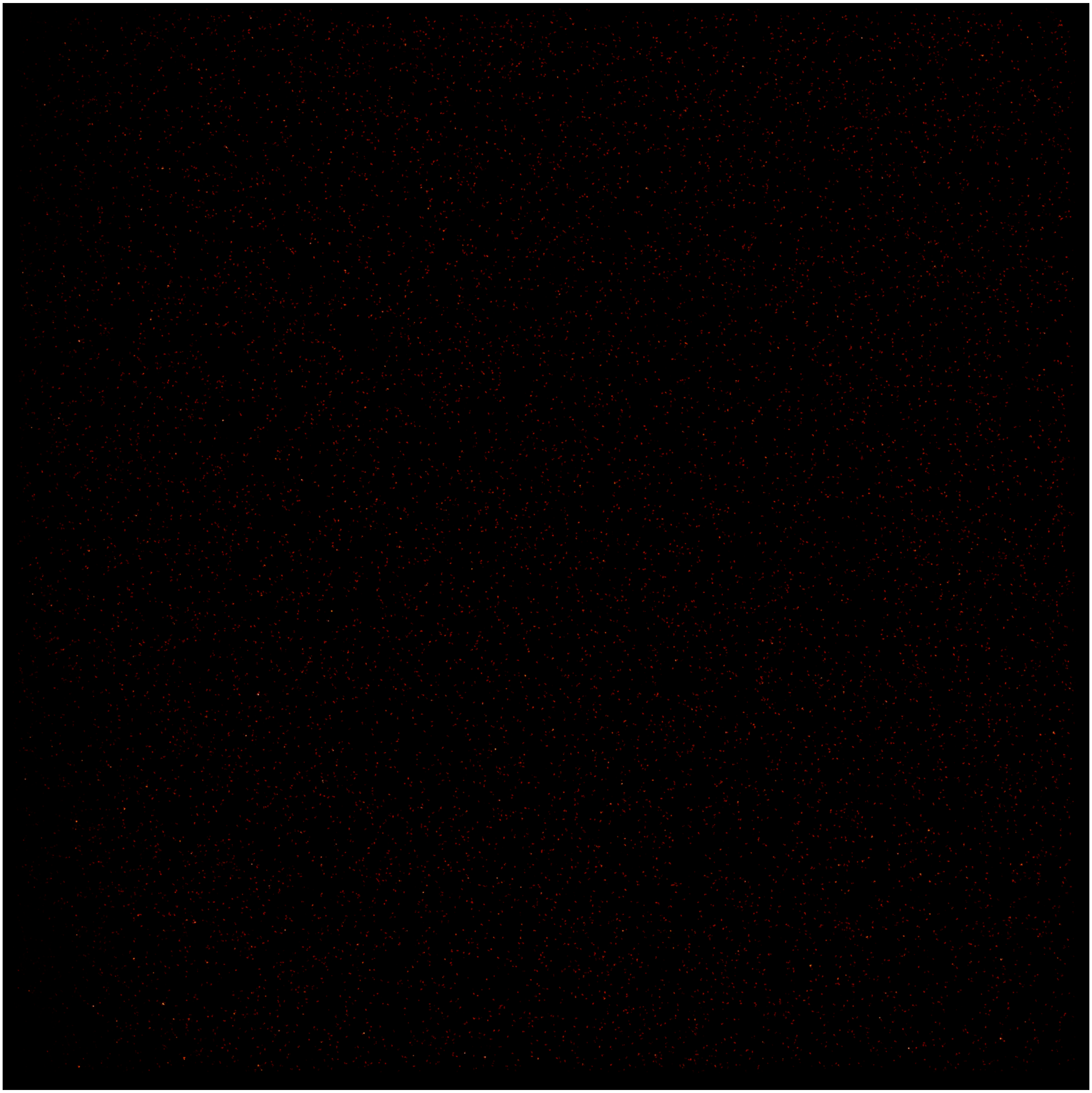

Supplementary Figure 12. Overview image of crosstalk experiment for imager sequence P12. Image size 40.96 μm.

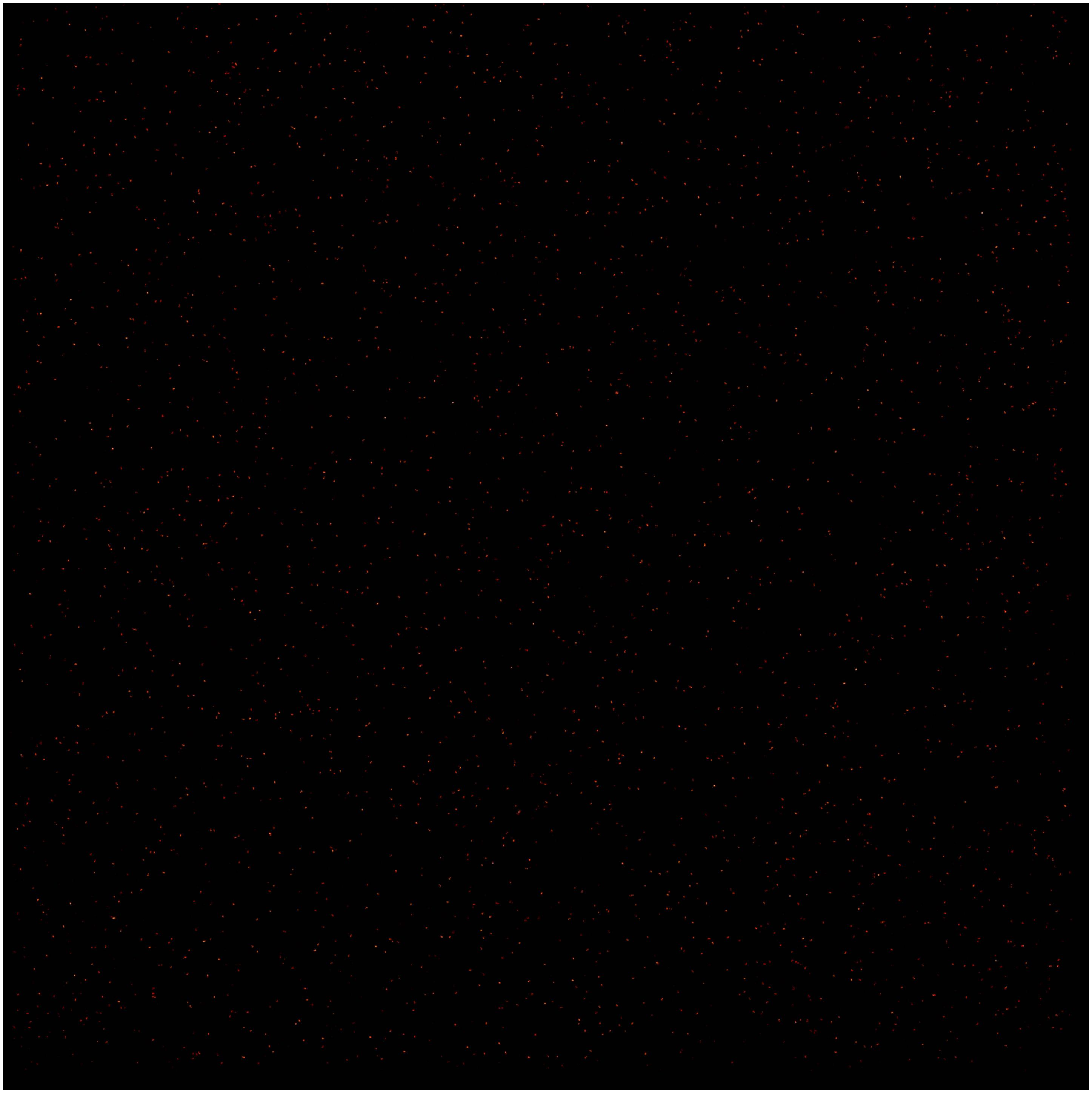

Supplementary Figure 13. Overview image of crosstalk experiment for imager sequence P13. Image size 40.96 µm.

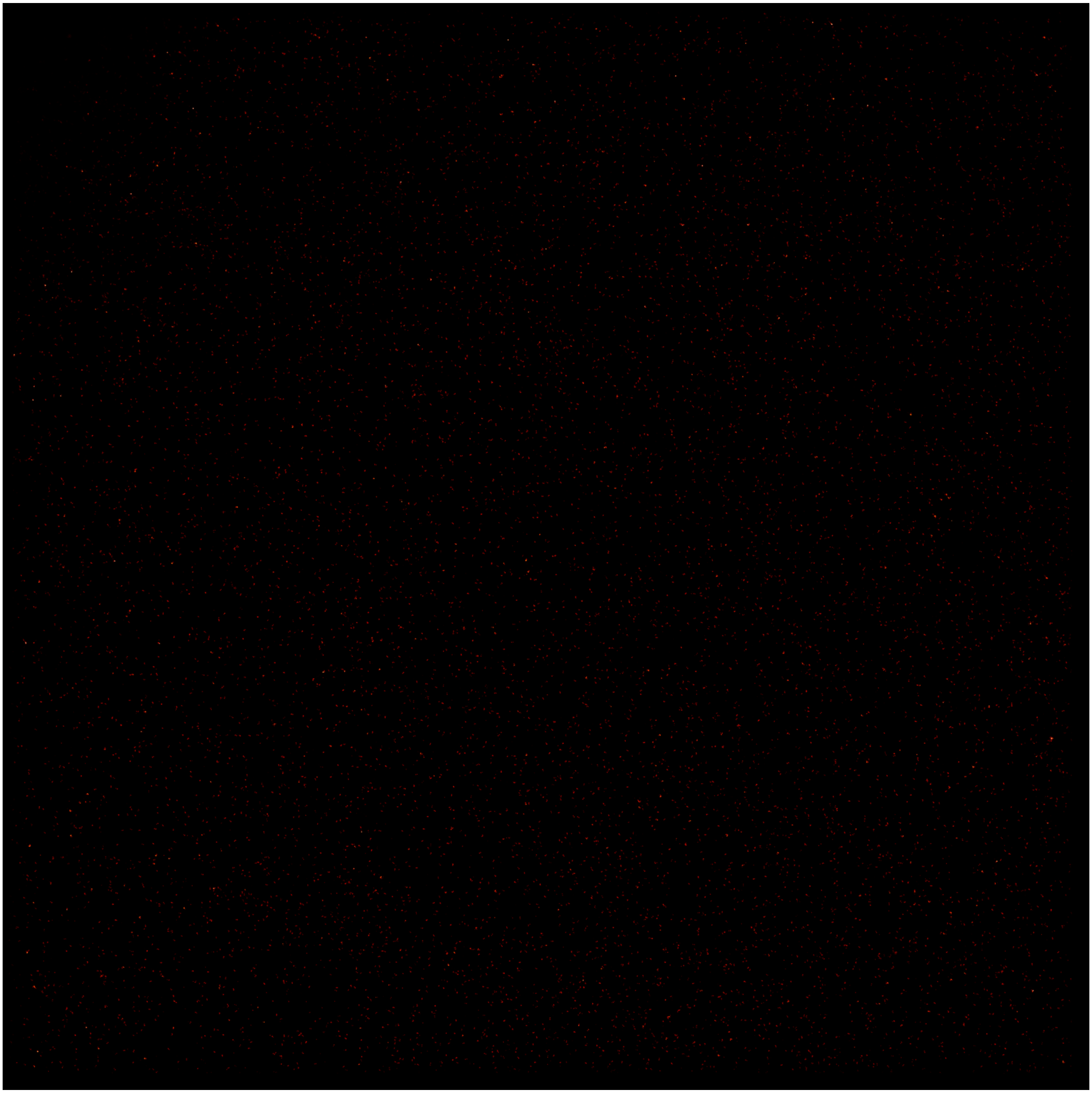

Supplementary Figure 14. Overview image of crosstalk experiment for imager sequence P14. Image size 40.96 µm.

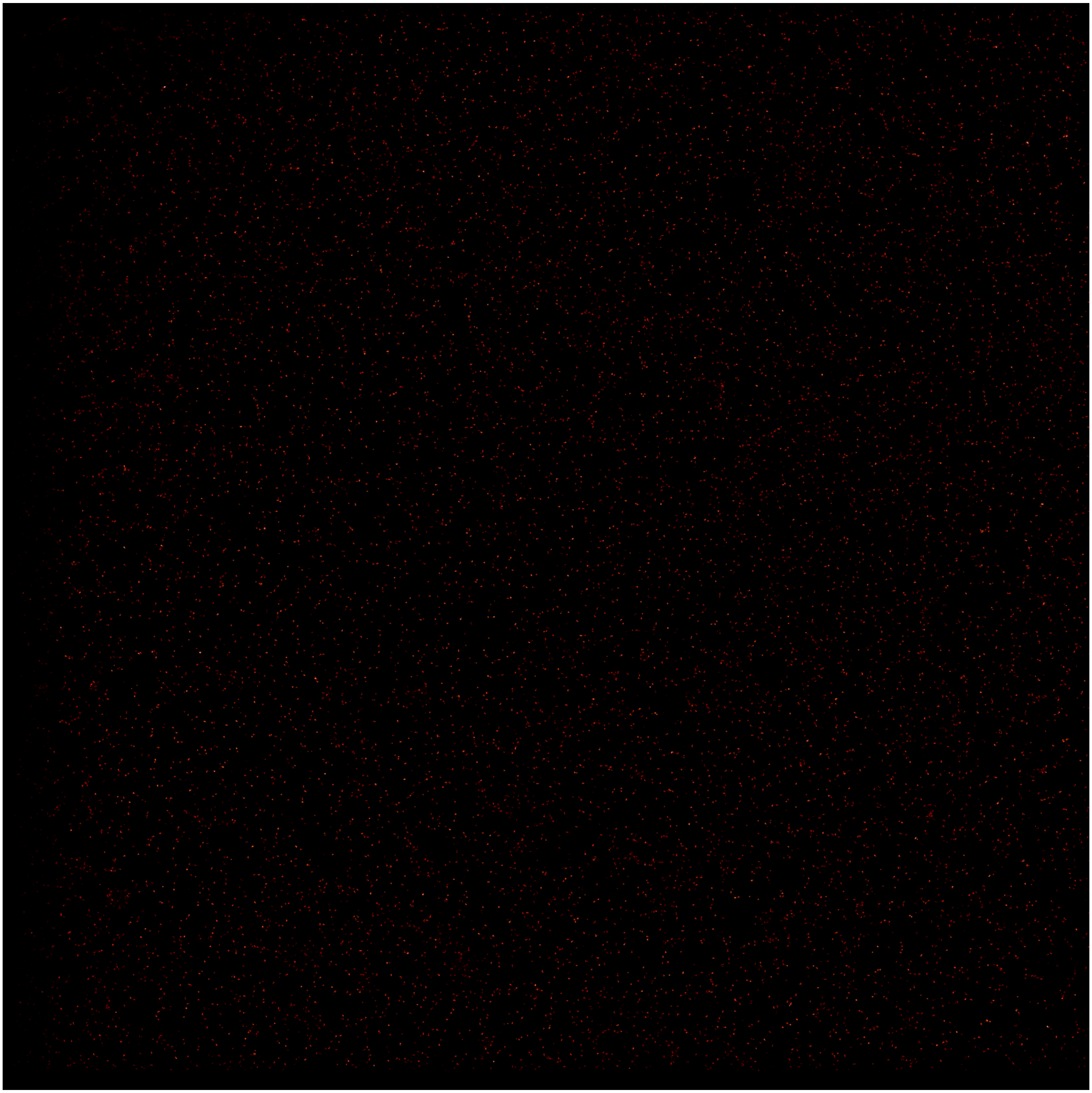

Supplementary Figure 15. Overview image of crosstalk experiment for imager sequence P15. Image size 40.96 μm.

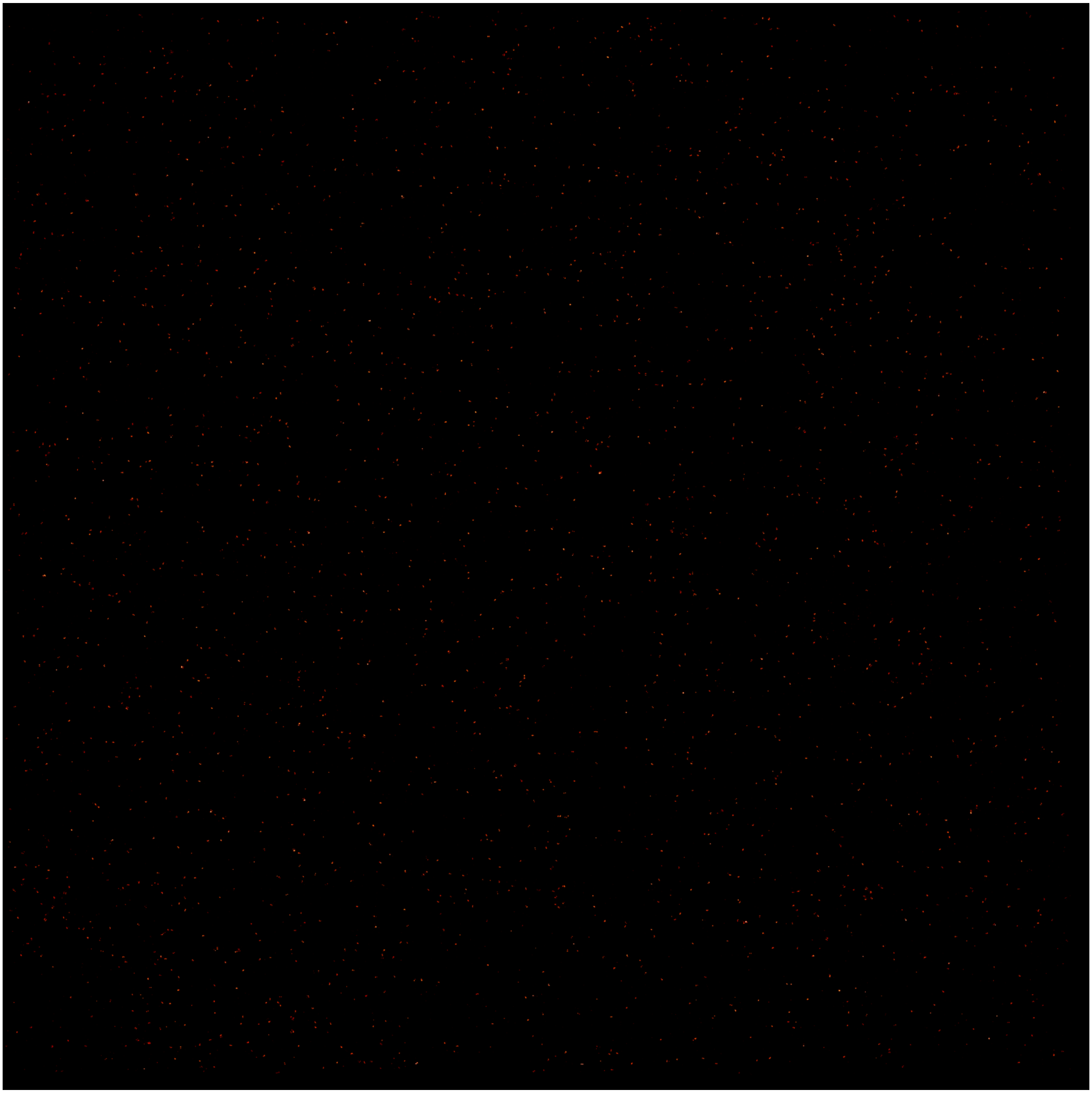

Supplementary Figure 16. Overview image of crosstalk experiment for imager sequence P16. Image size 40.96 µm.

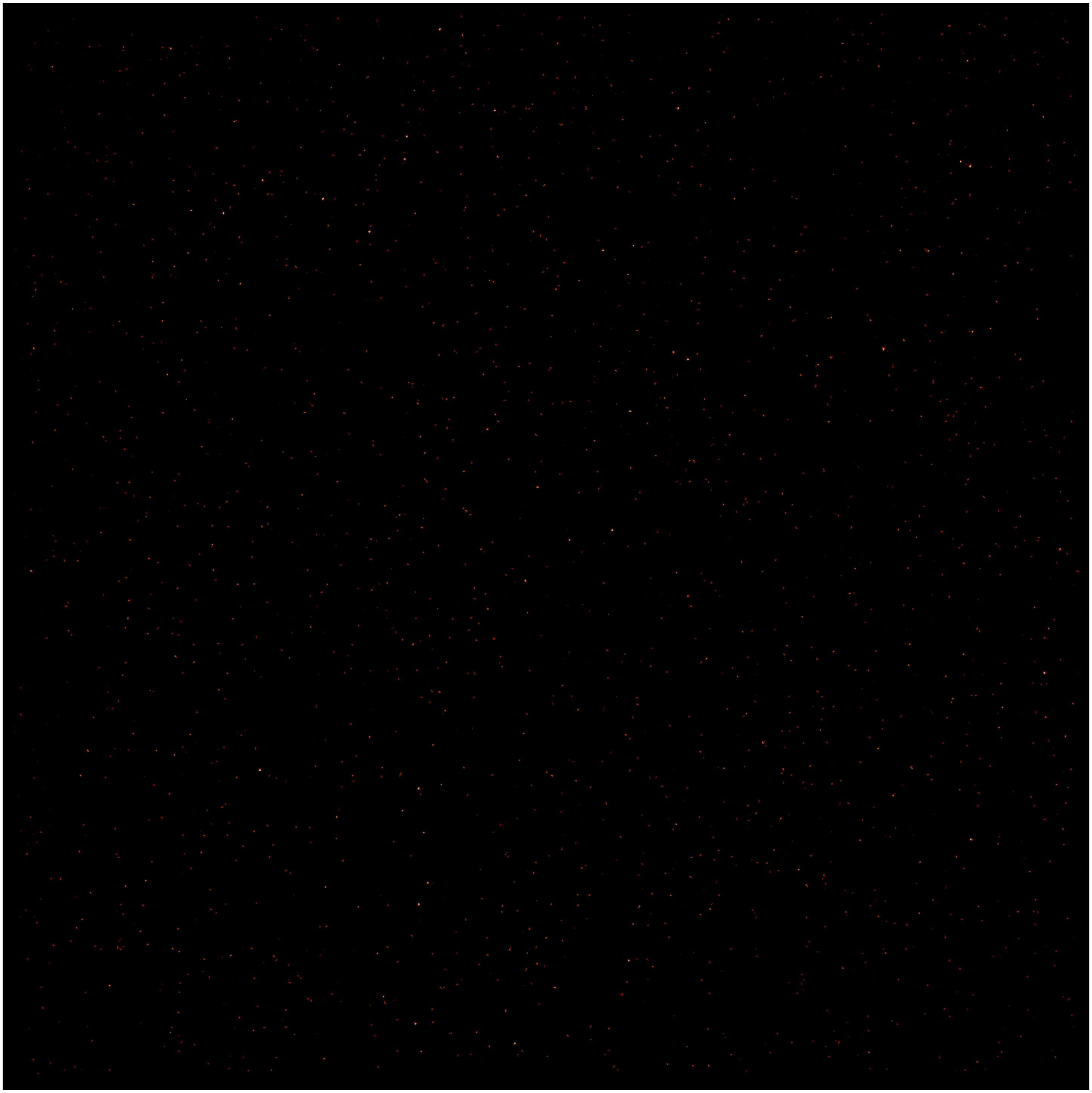

Supplementary Figure 17. Overview image of crosstalk experiment for imager sequence P17. Image size 40.96 µm.

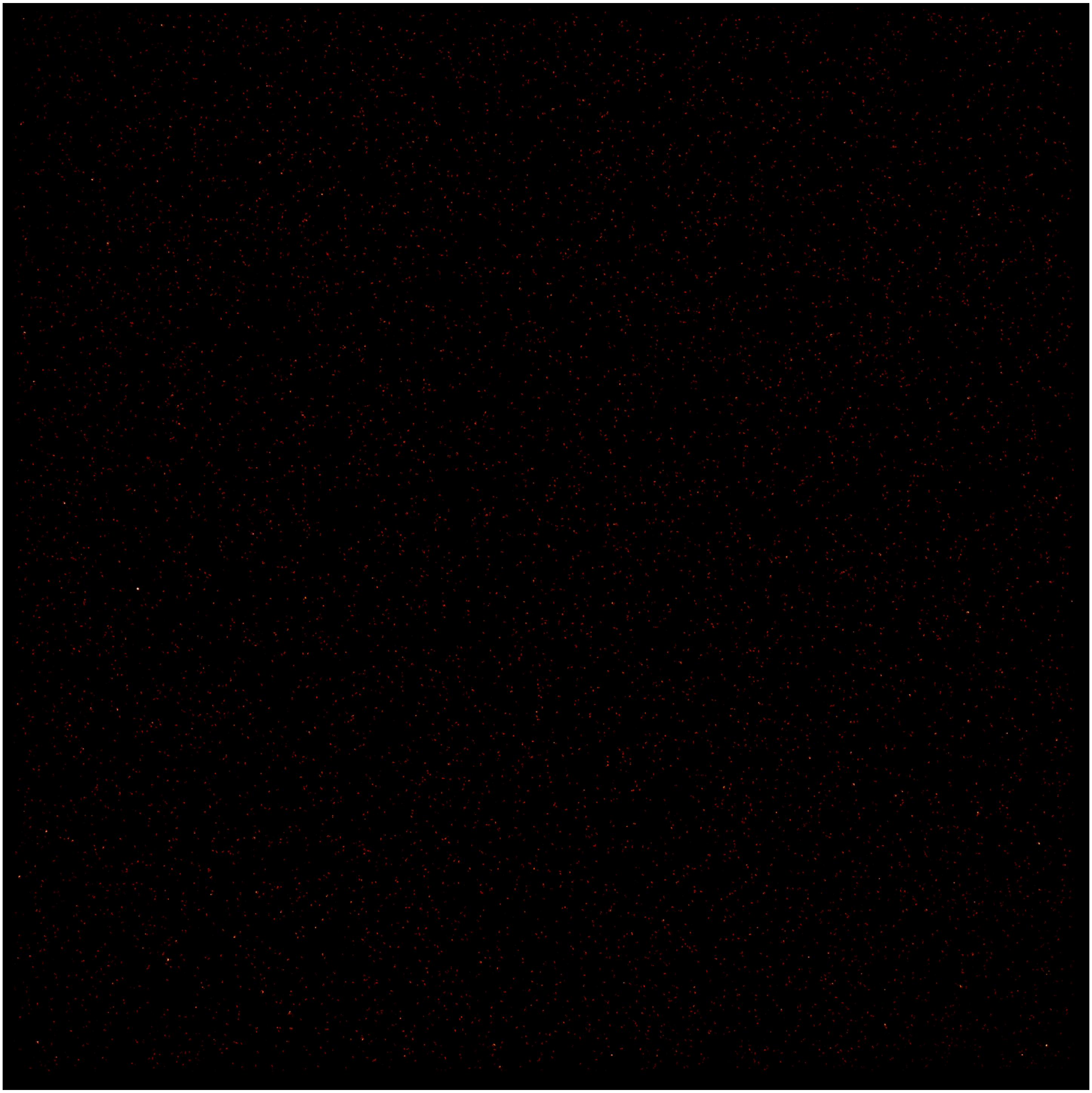

Supplementary Figure 18. Overview image of crosstalk experiment for imager sequence P18. Image size 40.96 µm.

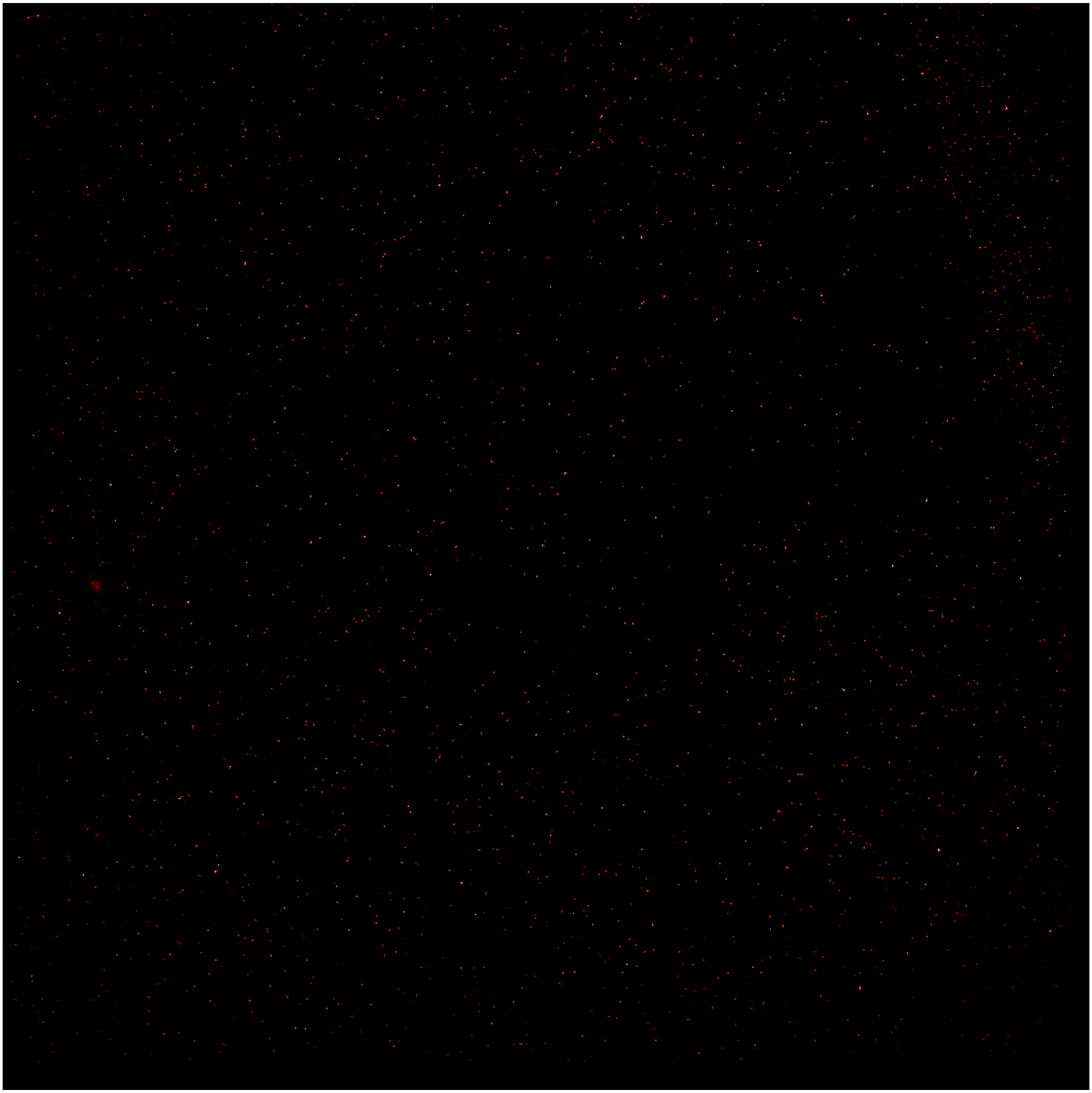

Supplementary Figure 19. Overview image of crosstalk experiment for imager sequence P19. Image size 40.96 µm.

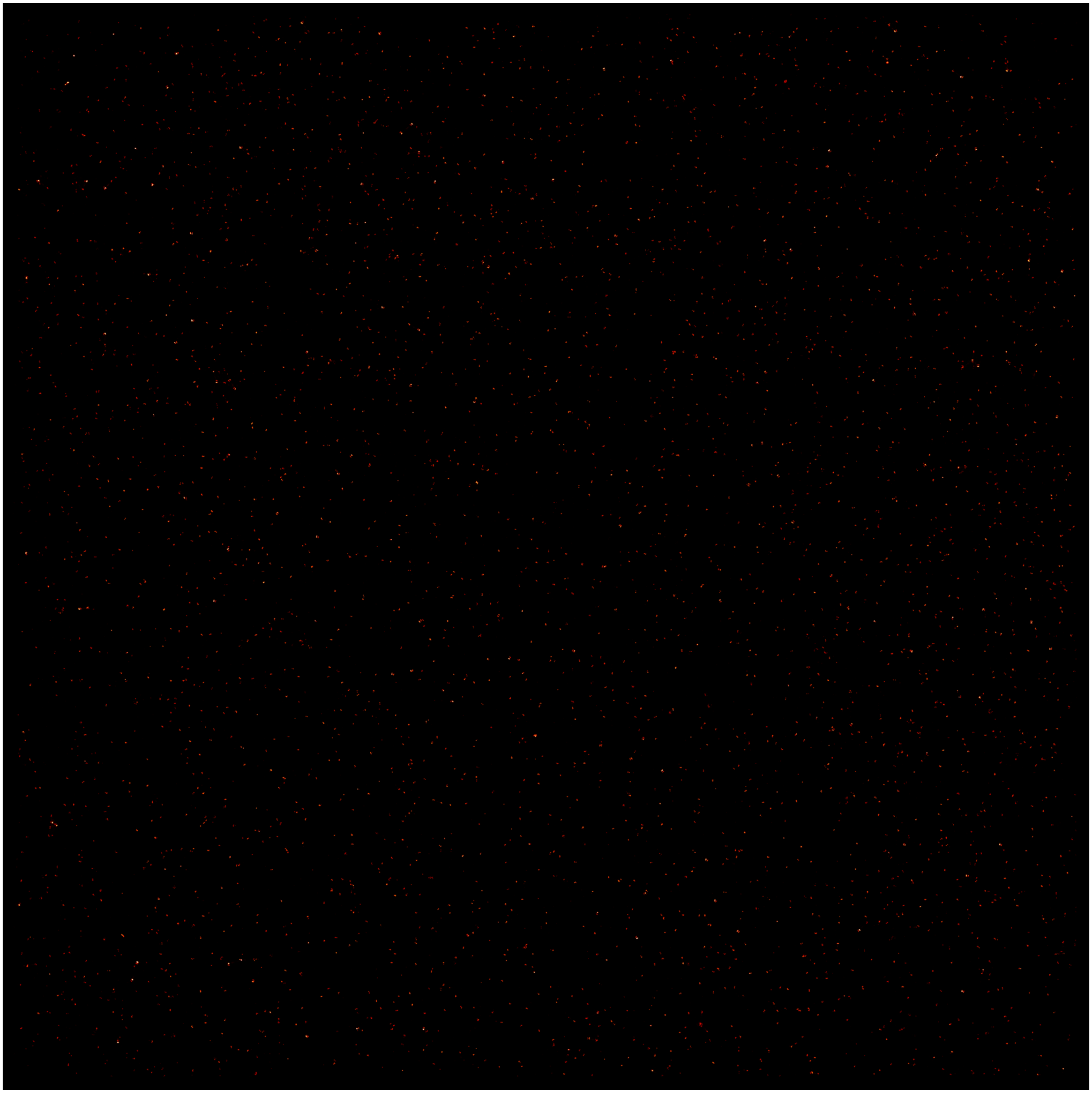

Supplementary Figure 20. Overview image of crosstalk experiment for imager sequence P20. Image size 40.96 µm.

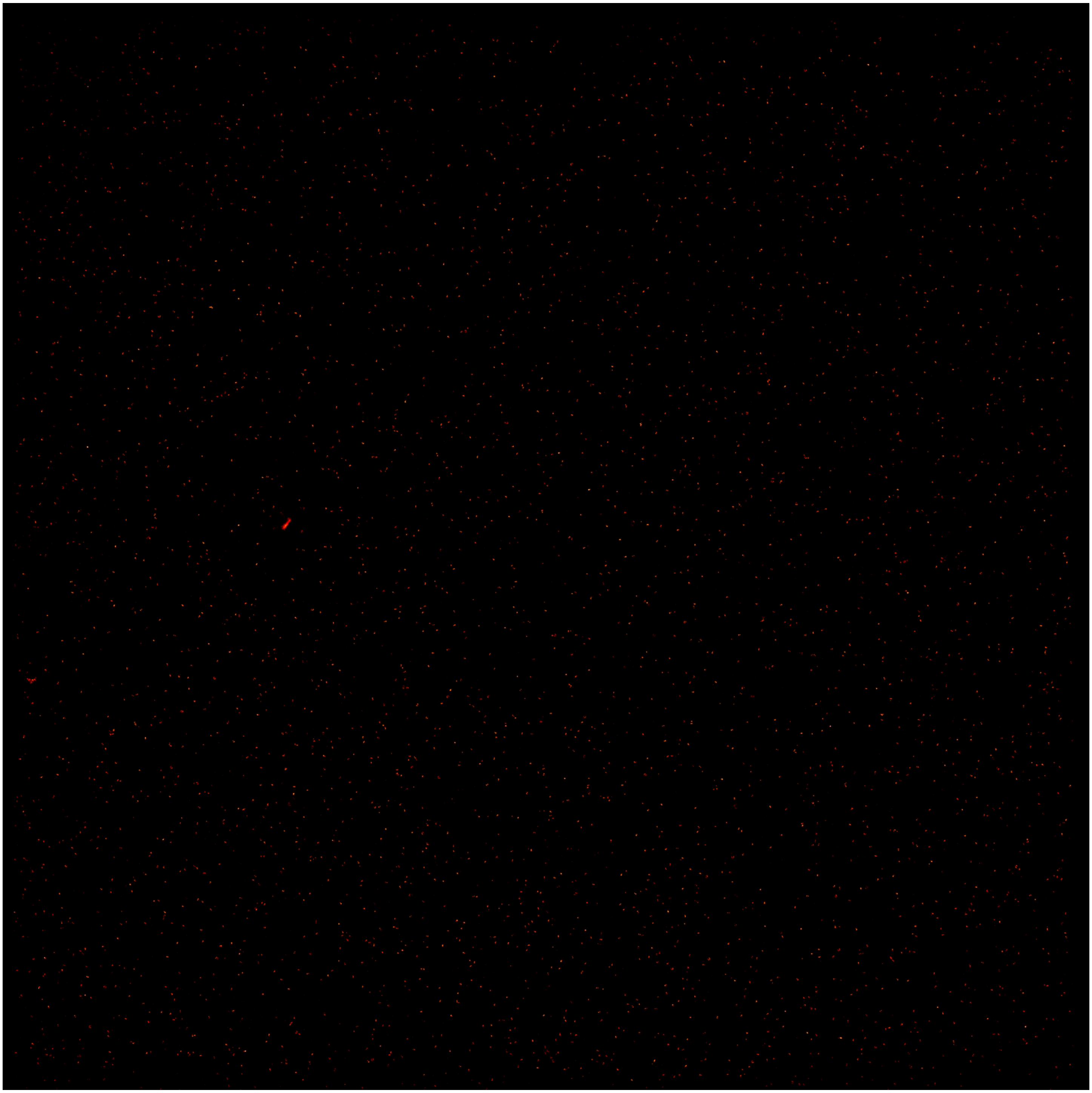

Supplementary Figure 21. Overview image of crosstalk experiment for imager sequence P21. Image size 40.96 μm.

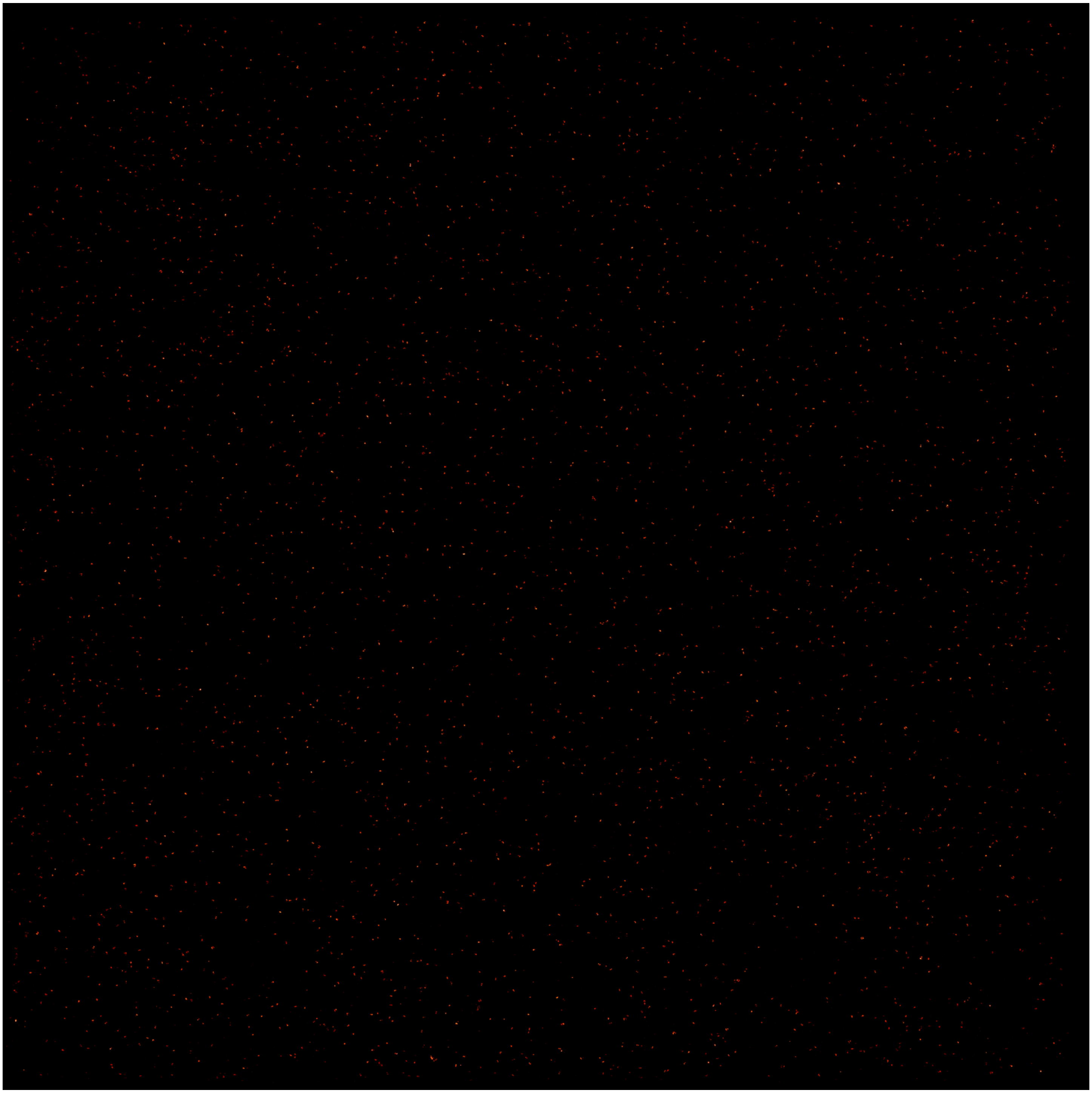

Supplementary Figure 22. Overview image of crosstalk experiment for imager sequence P22. Image size 40.96 µm.

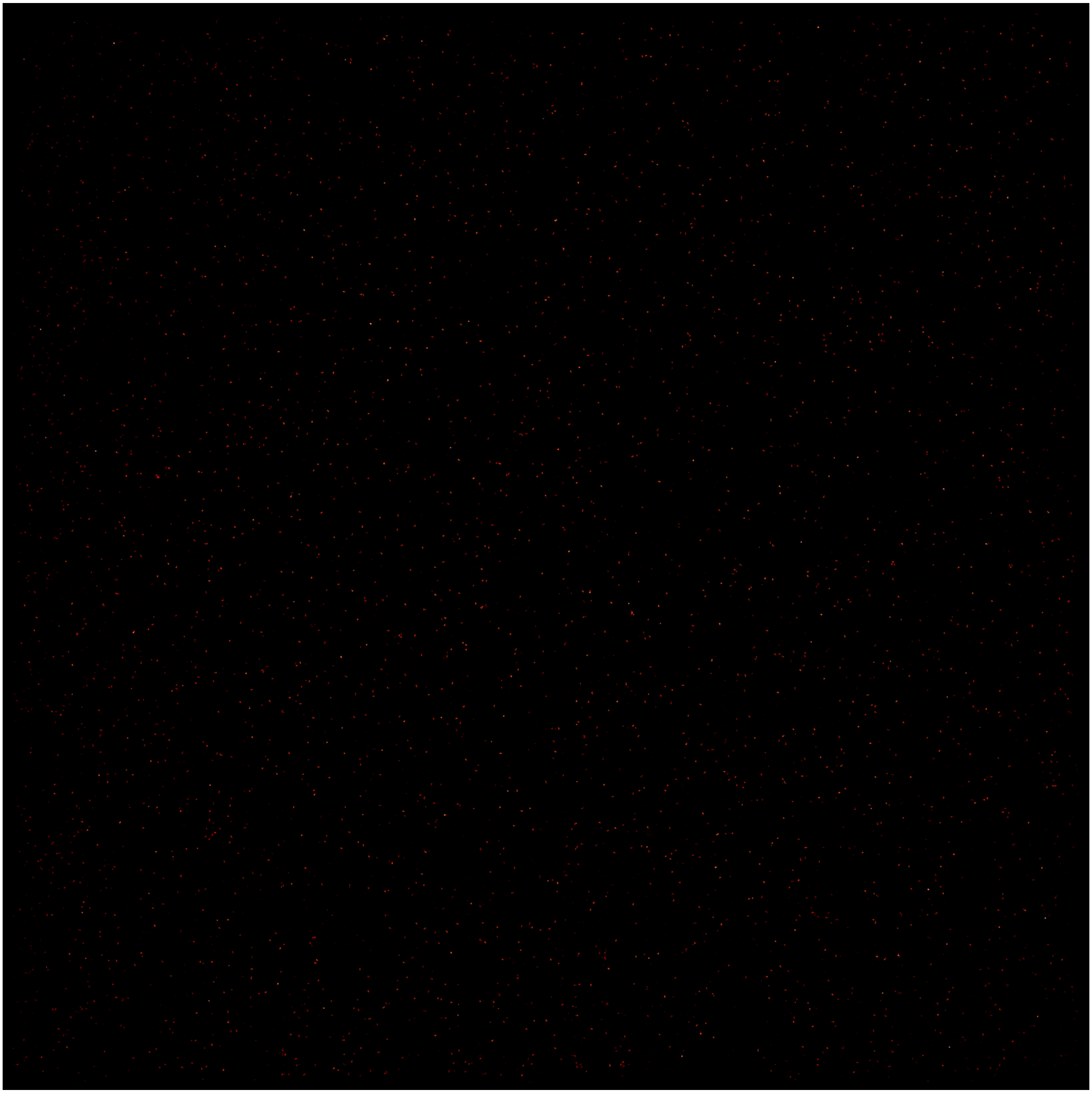

Supplementary Figure 23. Overview image of crosstalk experiment for imager sequence P23. Image size 40.96 µm.

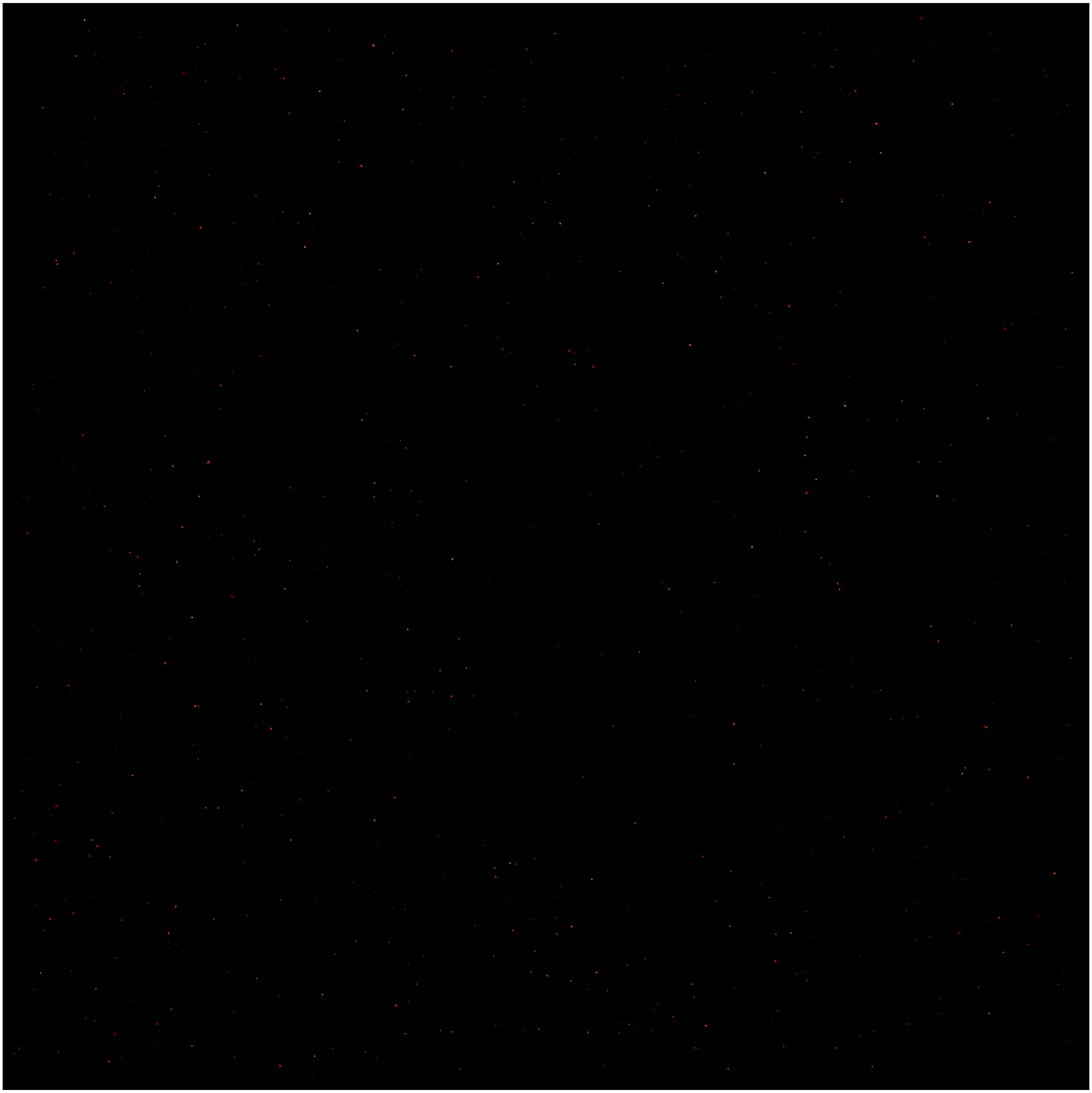

Supplementary Figure 24. Overview image of crosstalk experiment for imager sequence P24. Image size 40.96 µm.

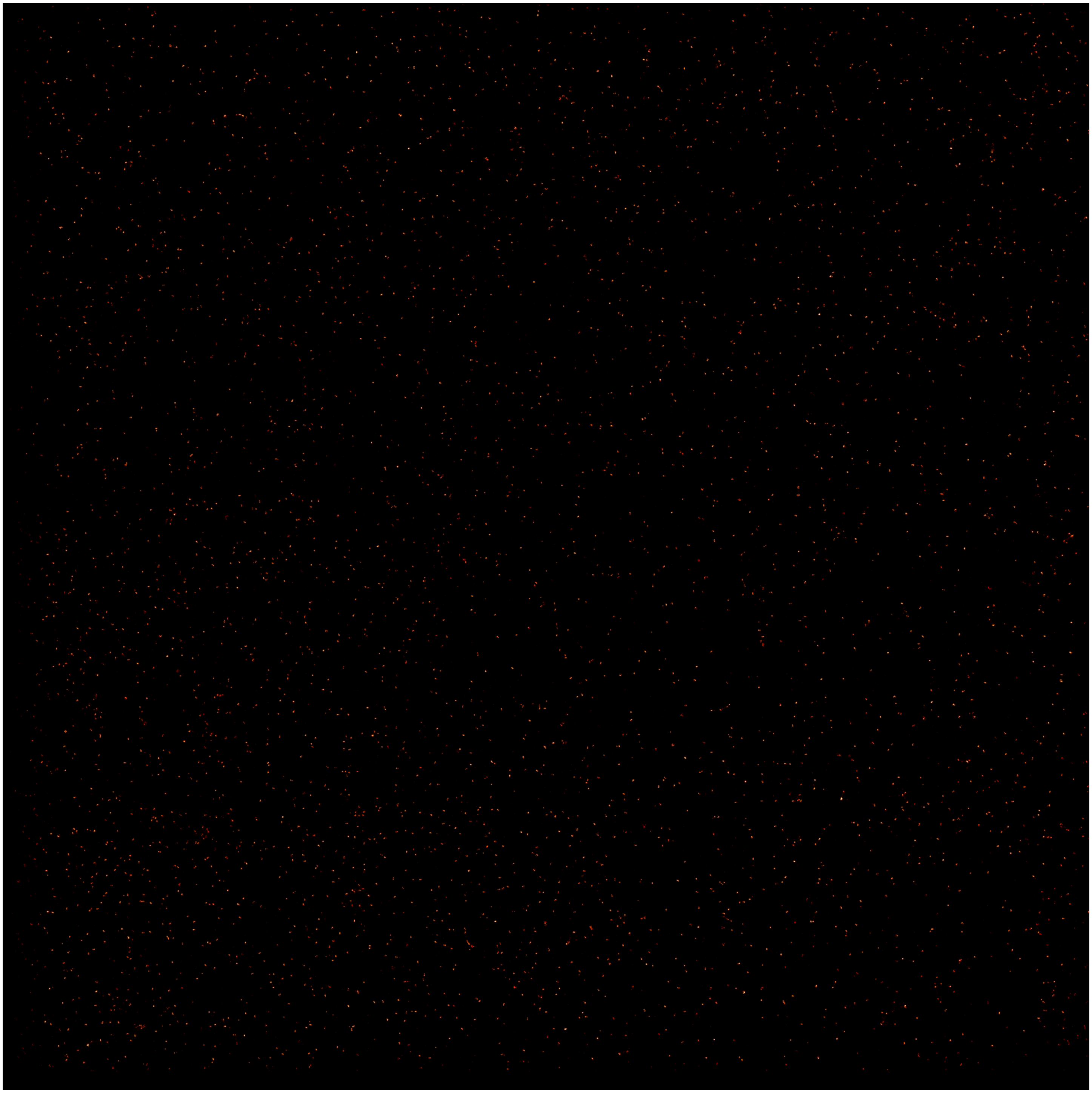

Supplementary Figure 25. Overview image of crosstalk experiment for imager sequence P25. Image size 40.96 µm.

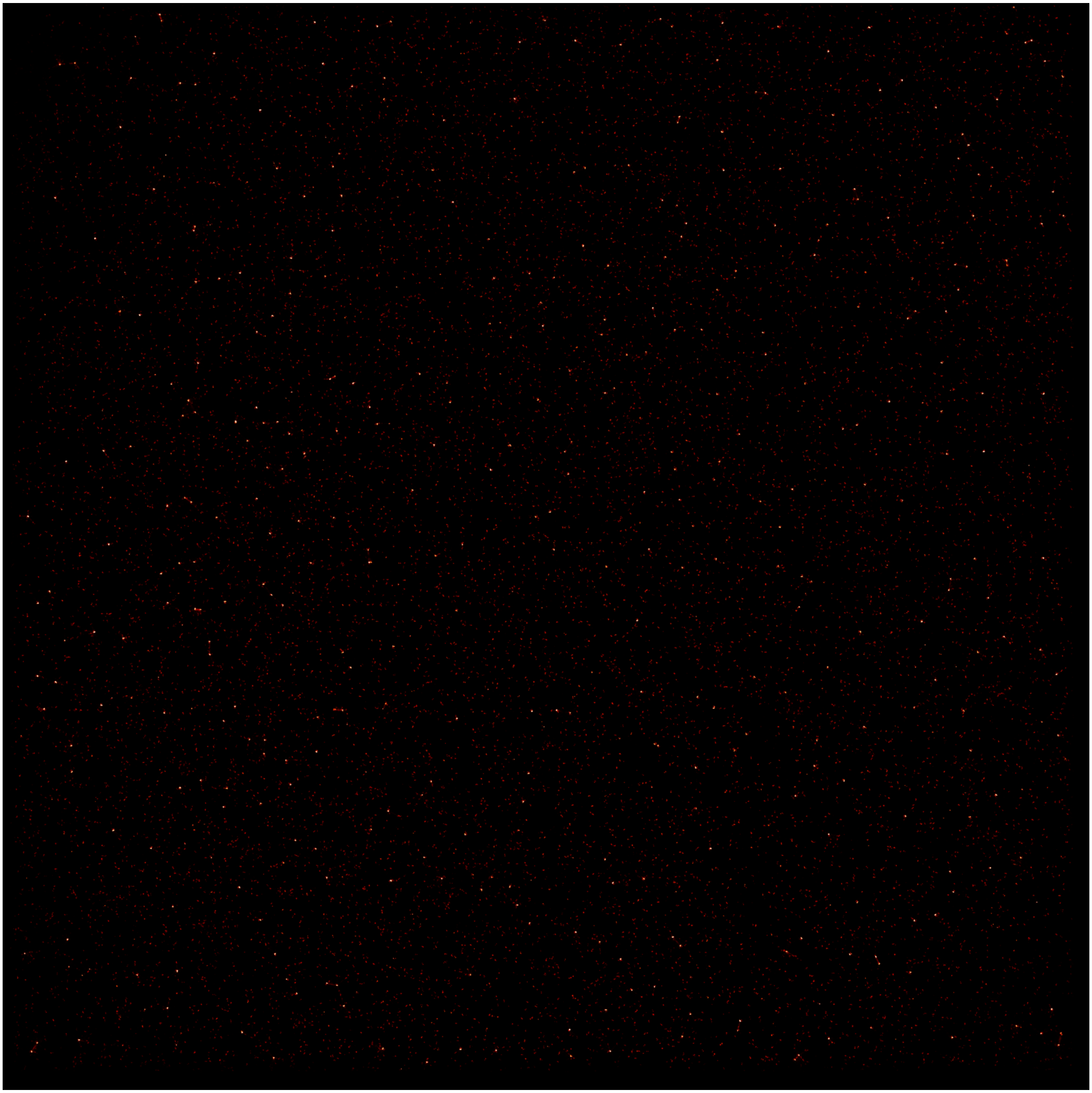

Supplementary Figure 26. Overview image of crosstalk experiment for imager sequence P26. Image size 40.96 µm.

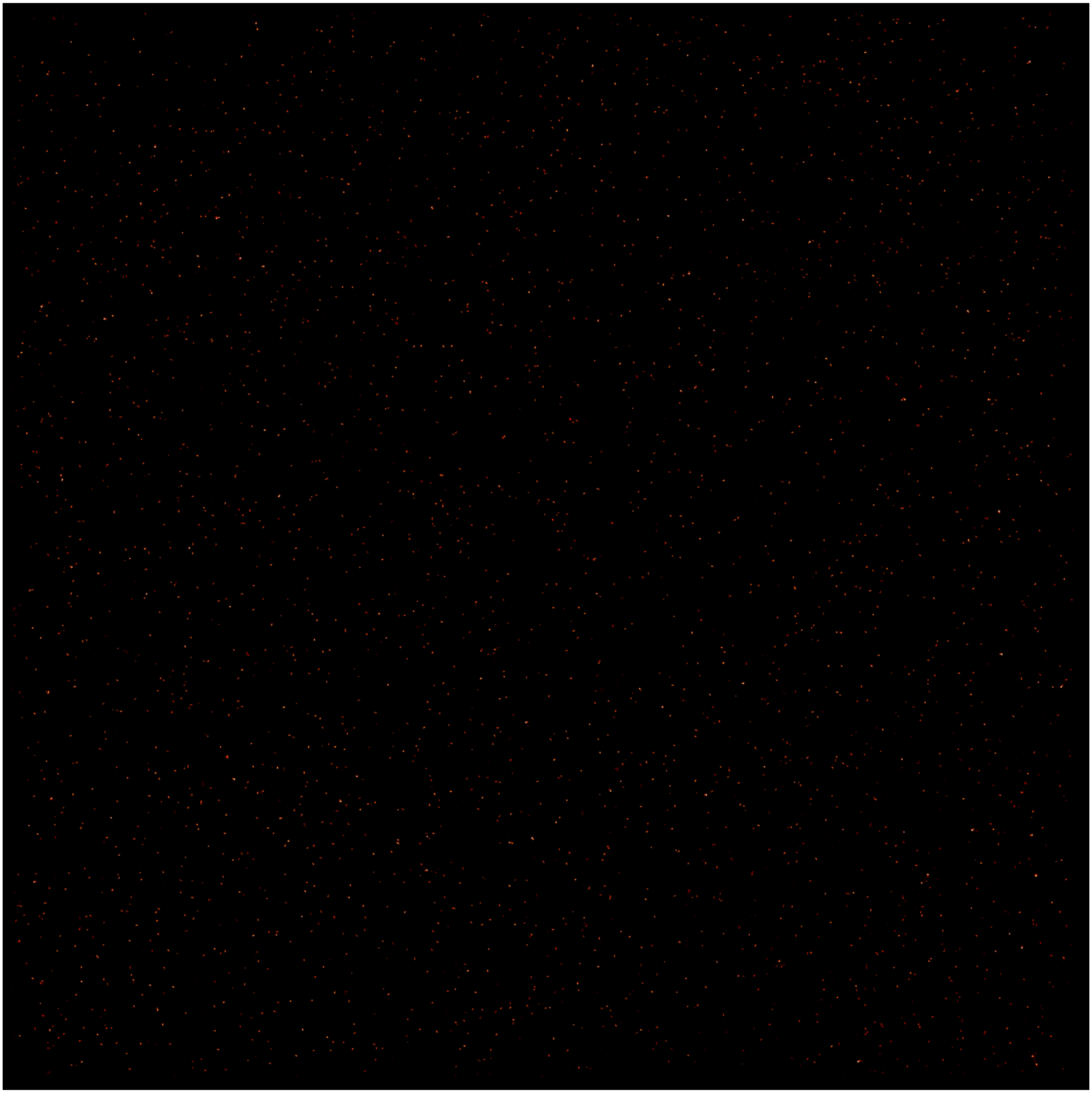

Supplementary Figure 27. Overview image of crosstalk experiment for imager sequence P27. Image size 40.96 µm.

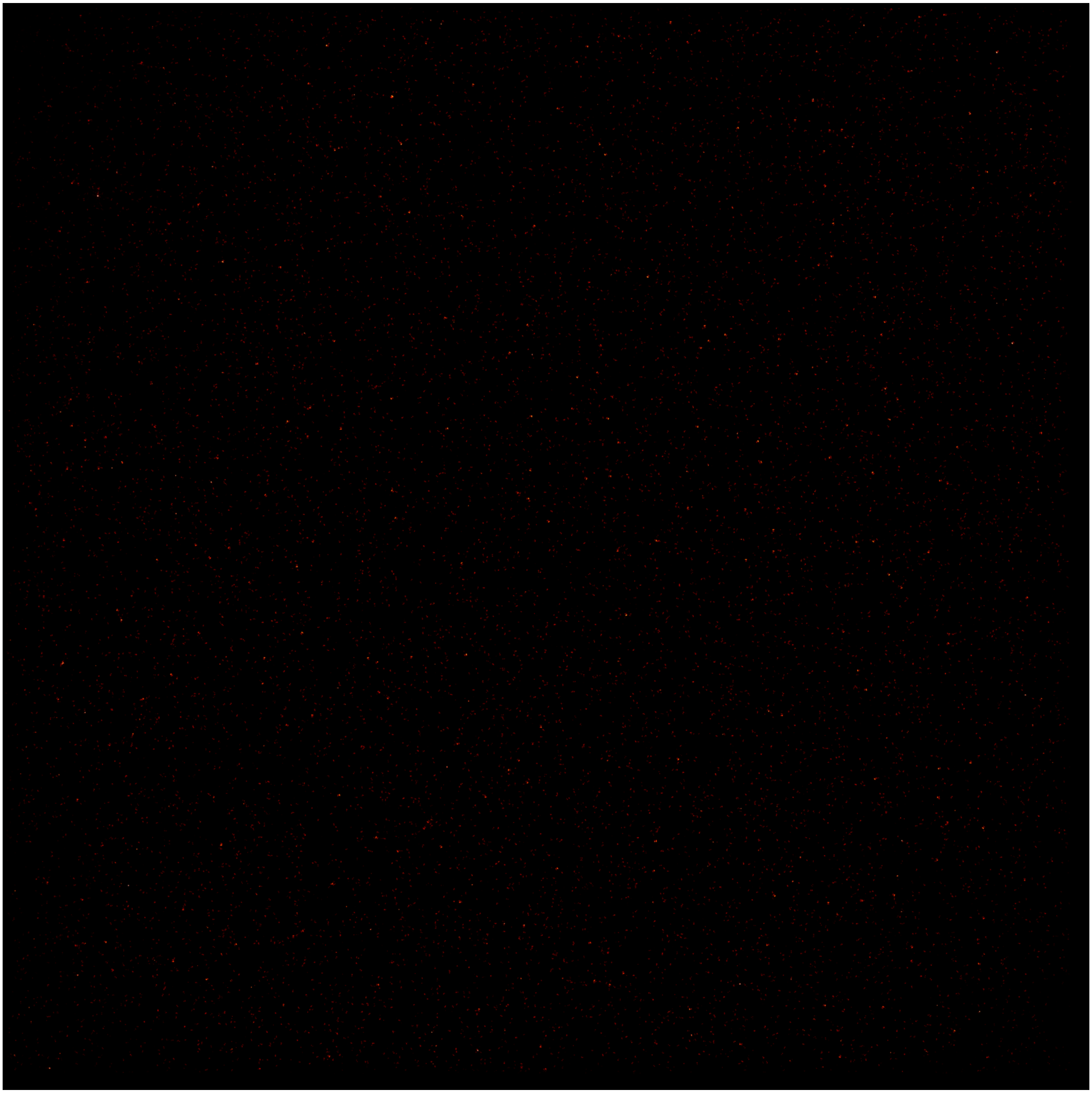

Supplementary Figure 28. Overview image of crosstalk experiment for imager sequence P28. Image size 40.96 µm.

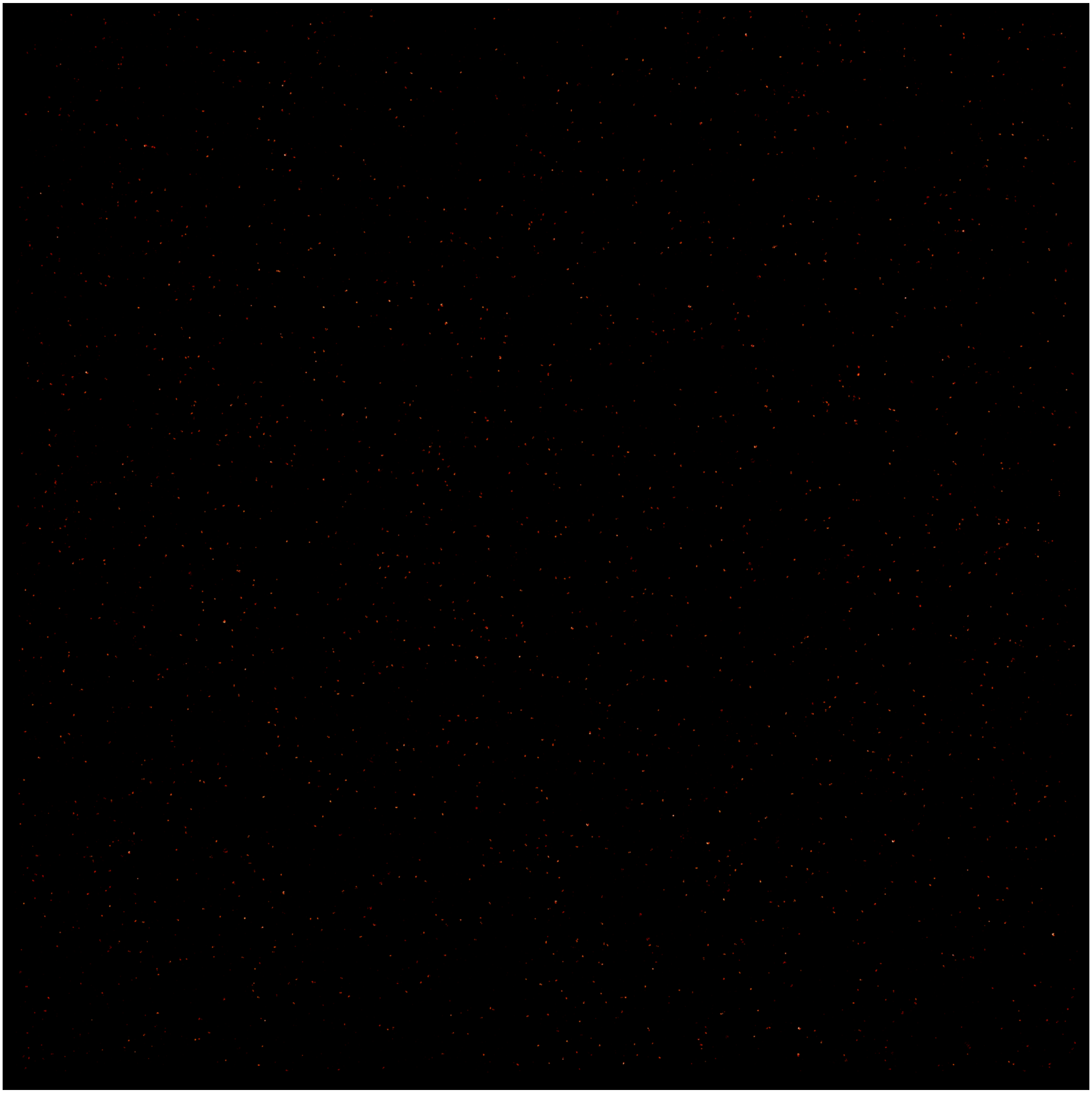

Supplementary Figure 29. Overview image of crosstalk experiment for imager sequence P29. Image size 40.96 µm.

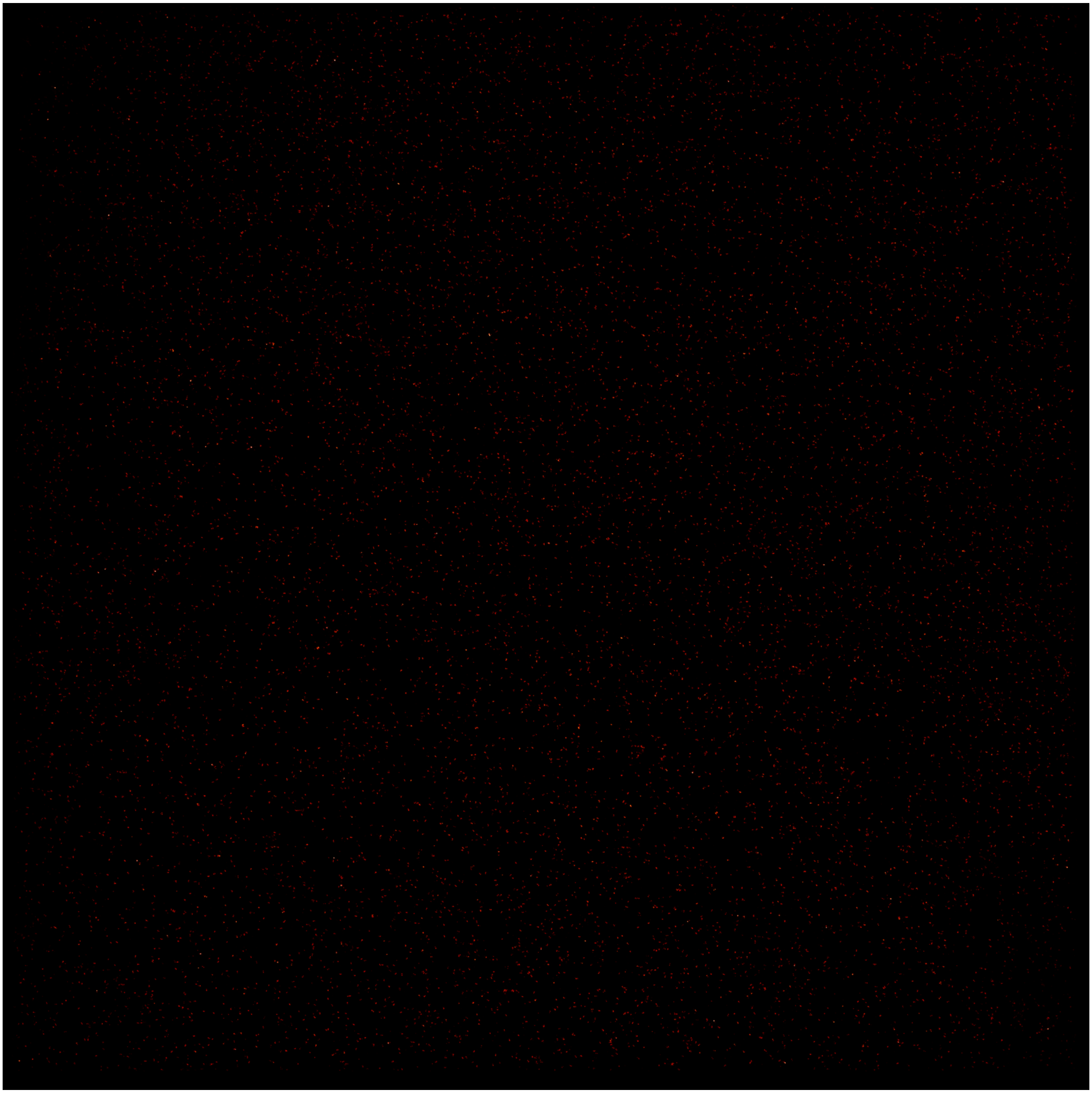

Supplementary Figure 30. Overview image of crosstalk experiment for imager sequence P30. Image size 40.96 µm.

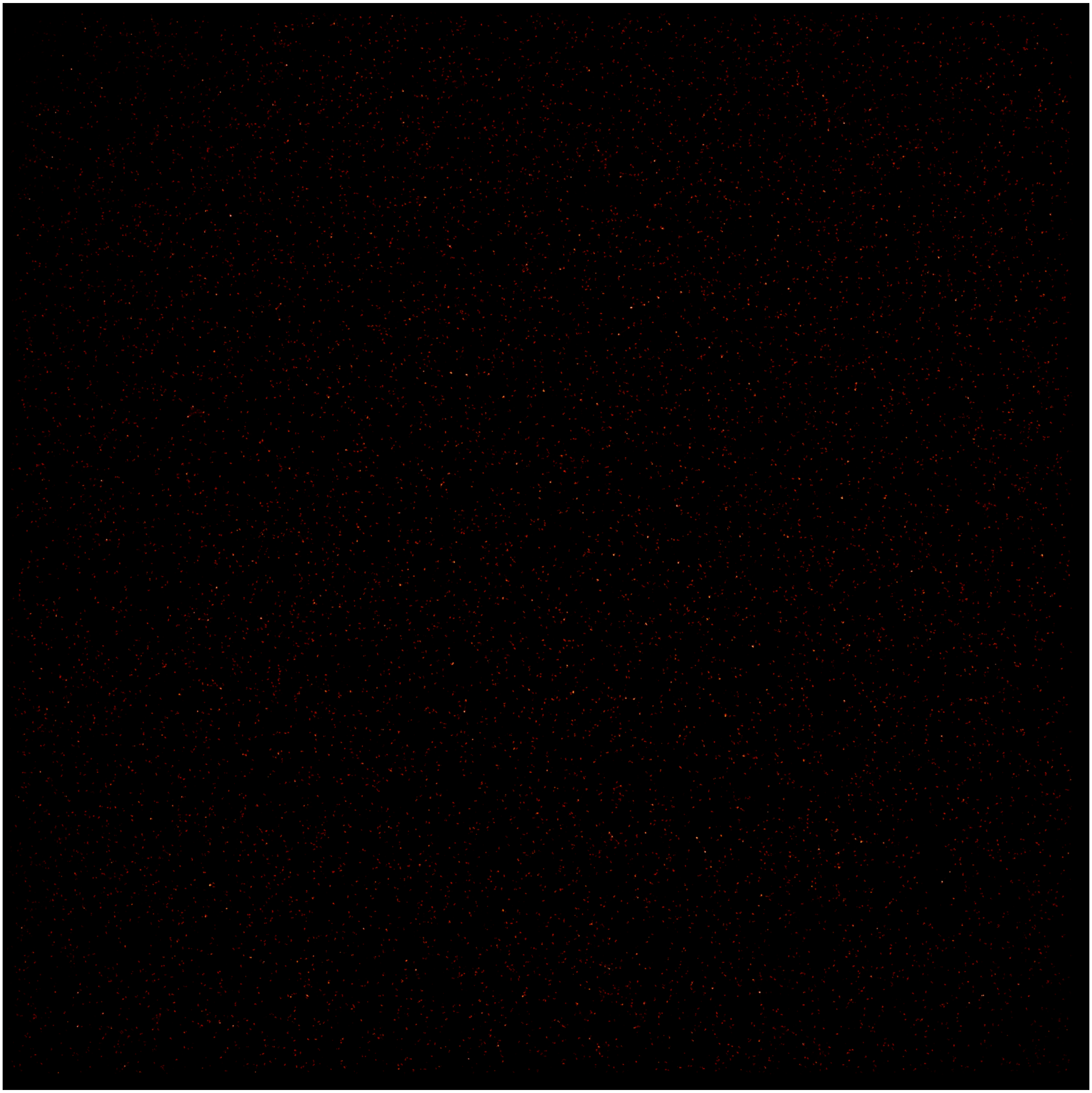

Supplementary Figure 31. Overview image of crosstalk experiment for imager sequence P31. Image size 40.96 µm.

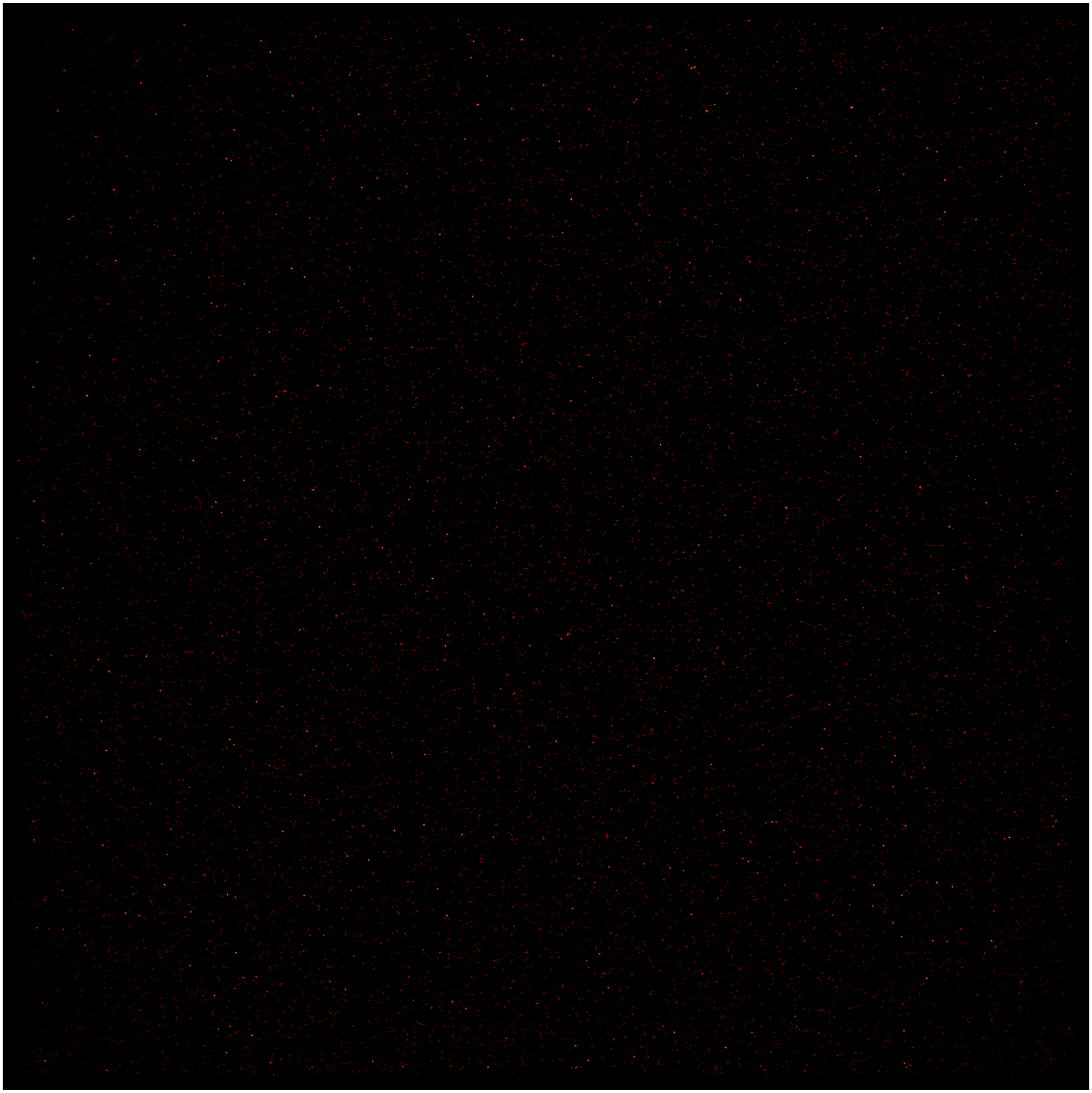

Supplementary Figure 32. Overview image of crosstalk experiment for imager sequence P32. Image size 40.96 µm.

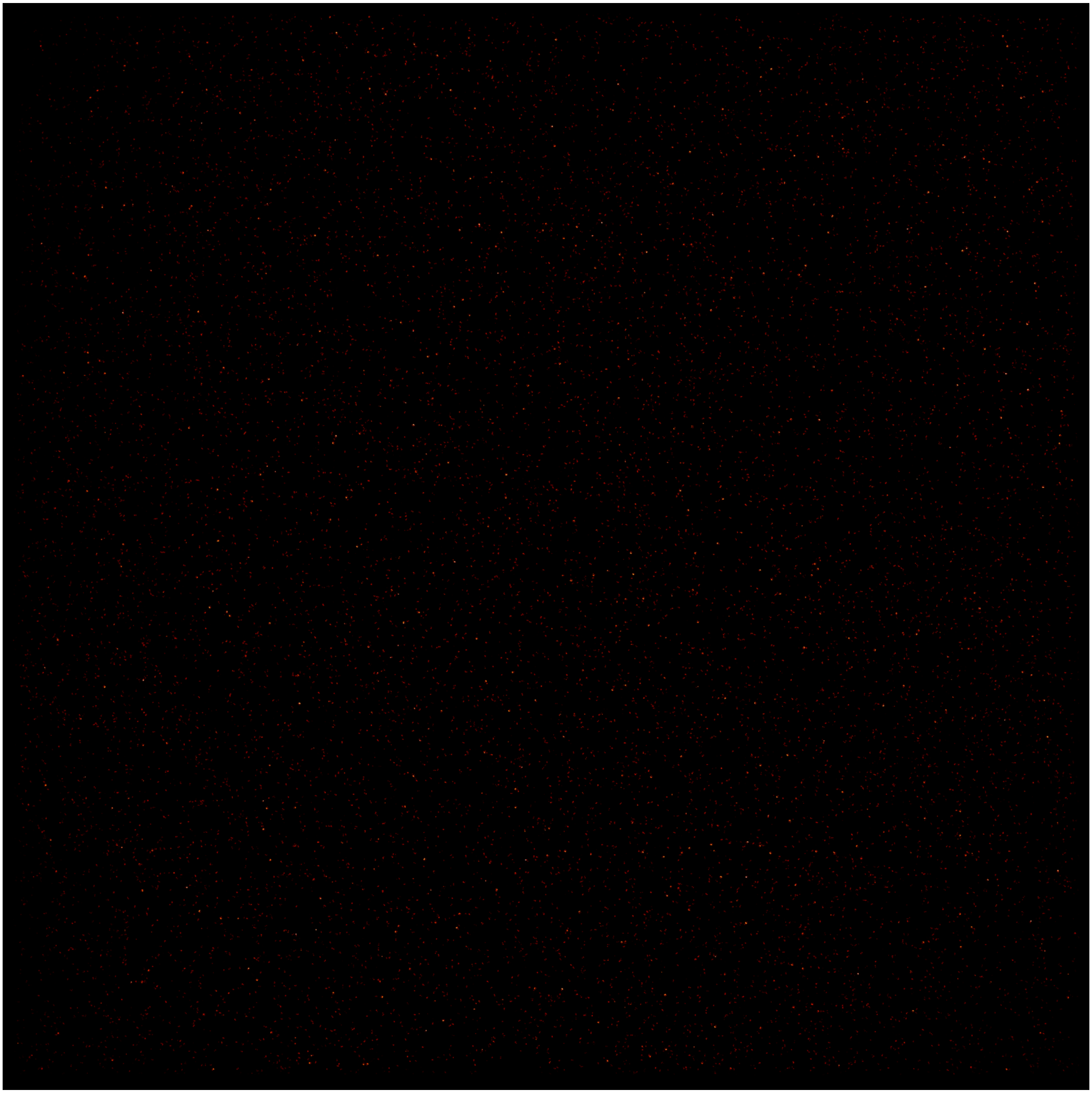

Supplementary Figure 33. Overview image of crosstalk experiment for imager sequence P33. Image size 40.96 µm.

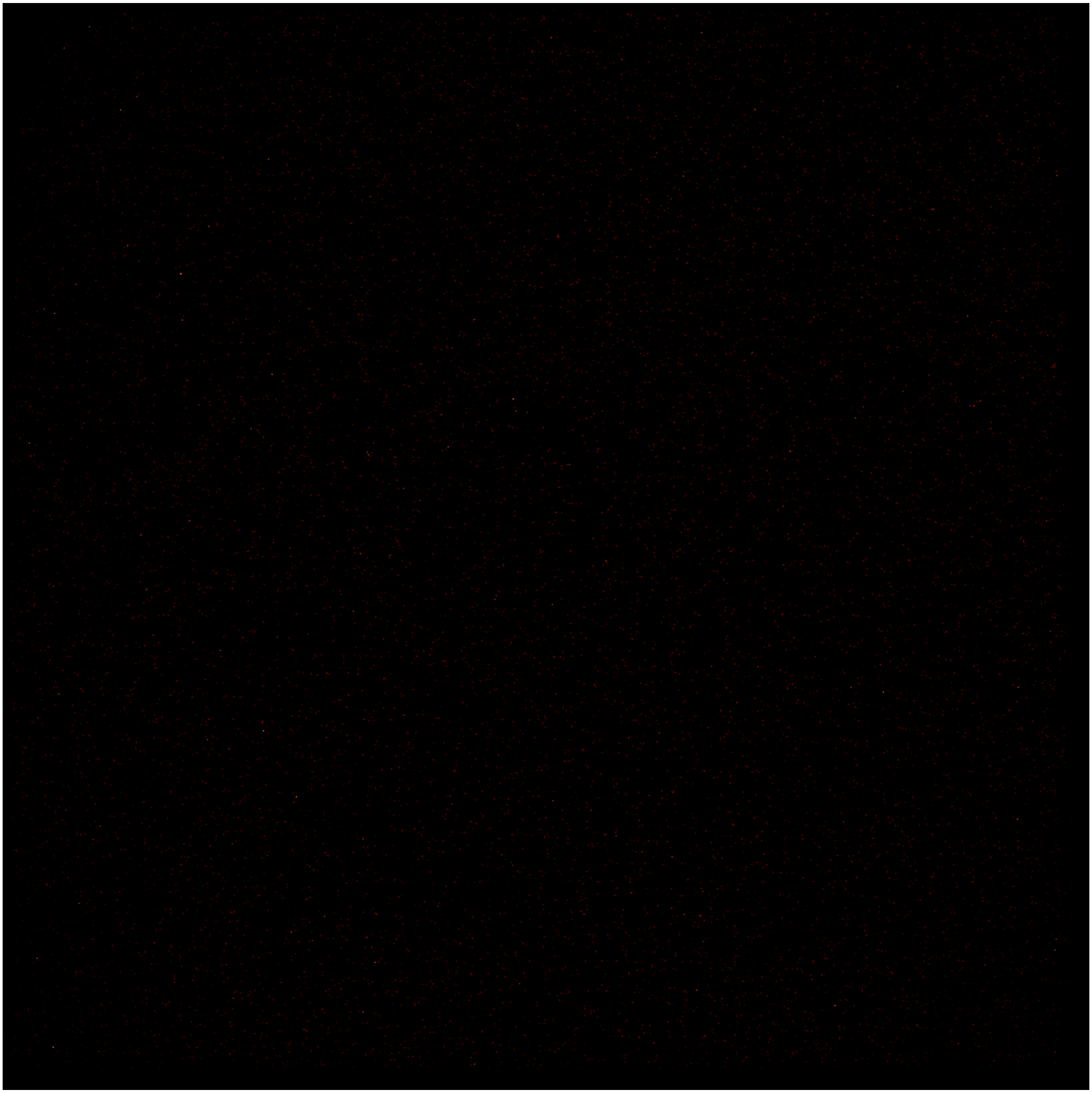

Supplementary Figure 34. Overview image of crosstalk experiment for imager sequence P34. Image size 40.96 µm.

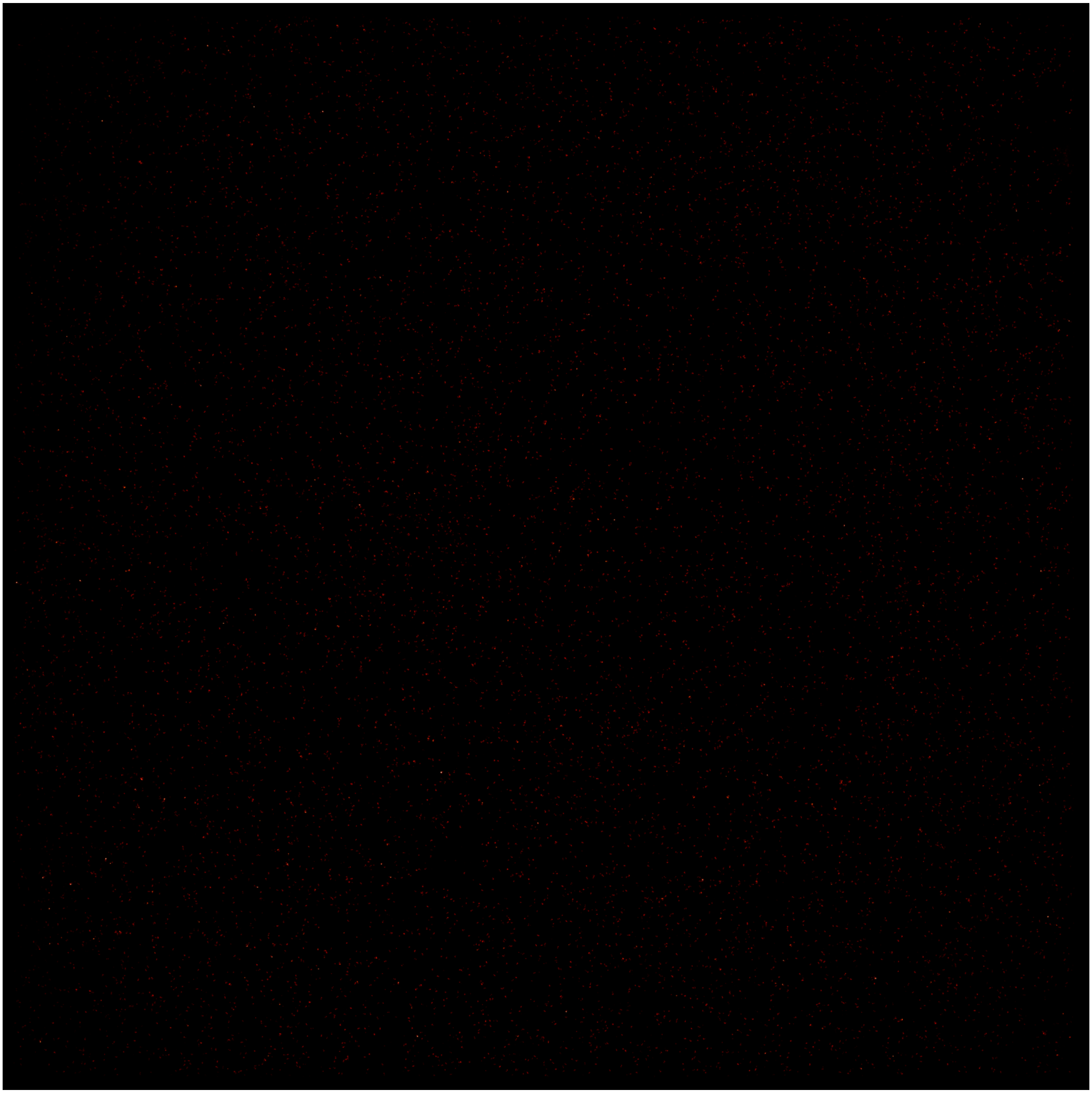

Supplementary Figure 35. Overview image of crosstalk experiment for imager sequence P35. Image size 40.96 µm.

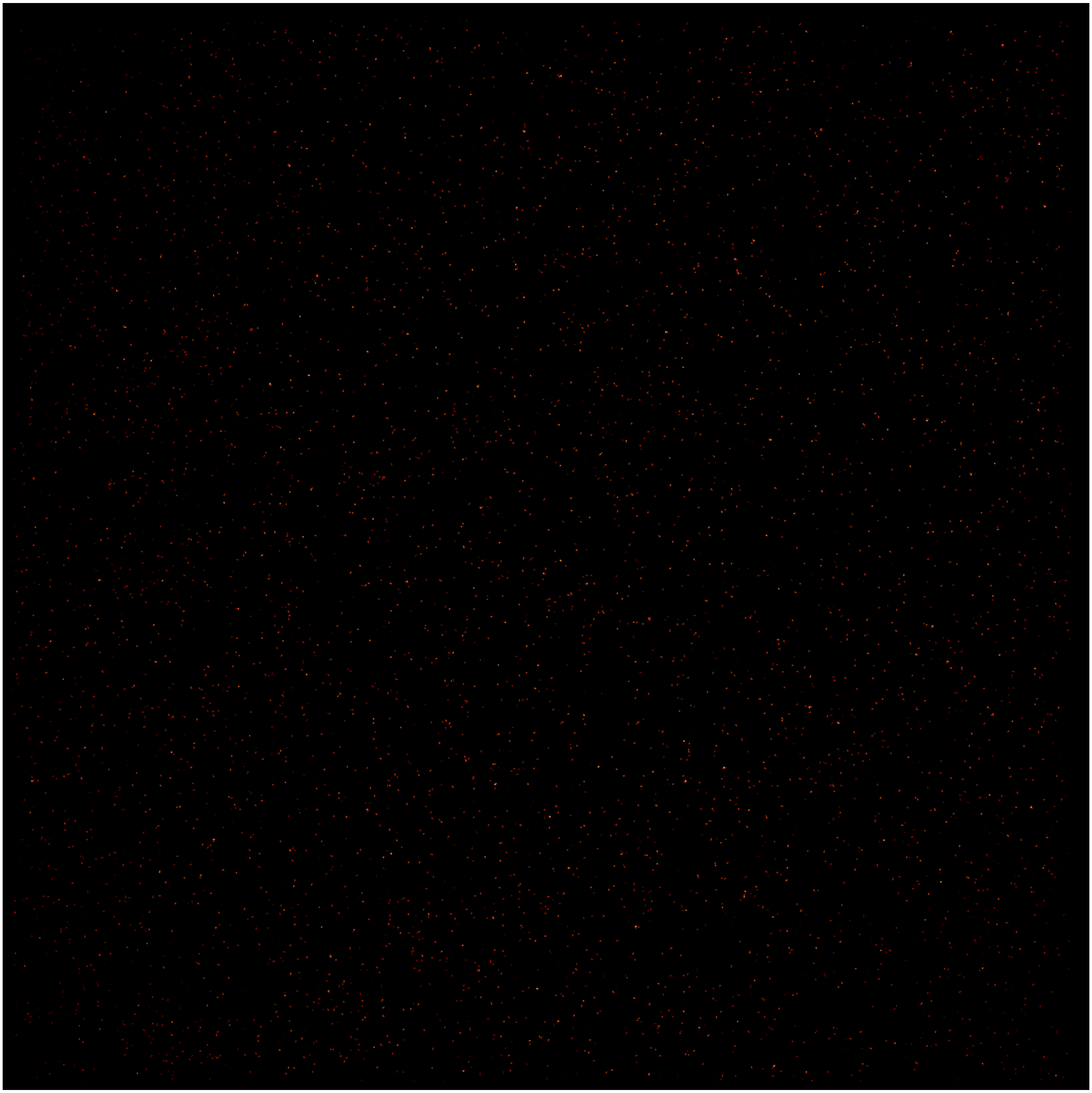

Supplementary Figure 36. Overview image of crosstalk experiment for imager sequence P36. Image size 40.96 μm.

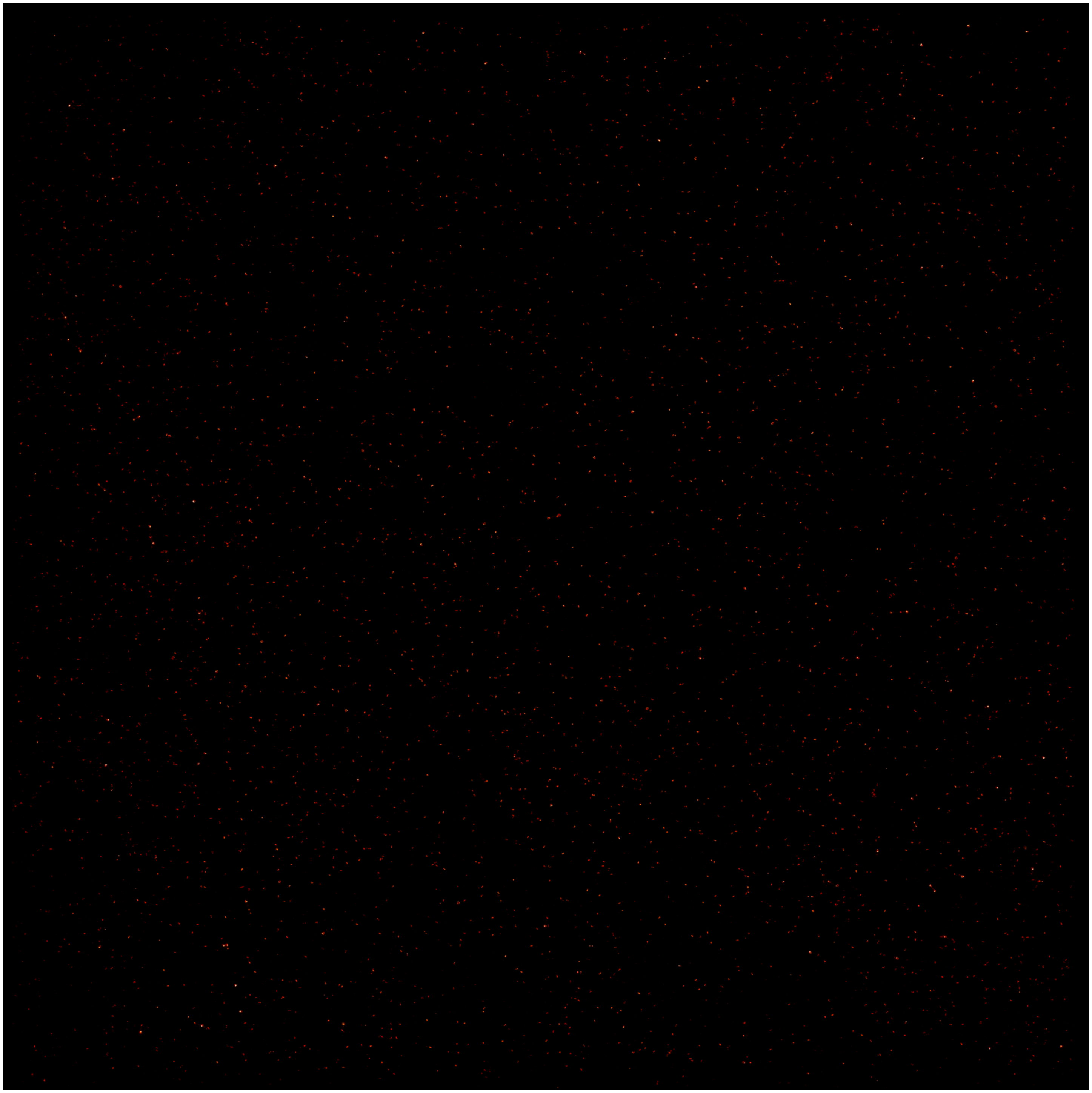

Supplementary Figure 37. Overview image of crosstalk experiment for imager sequence P37. Image size 40.96 µm.

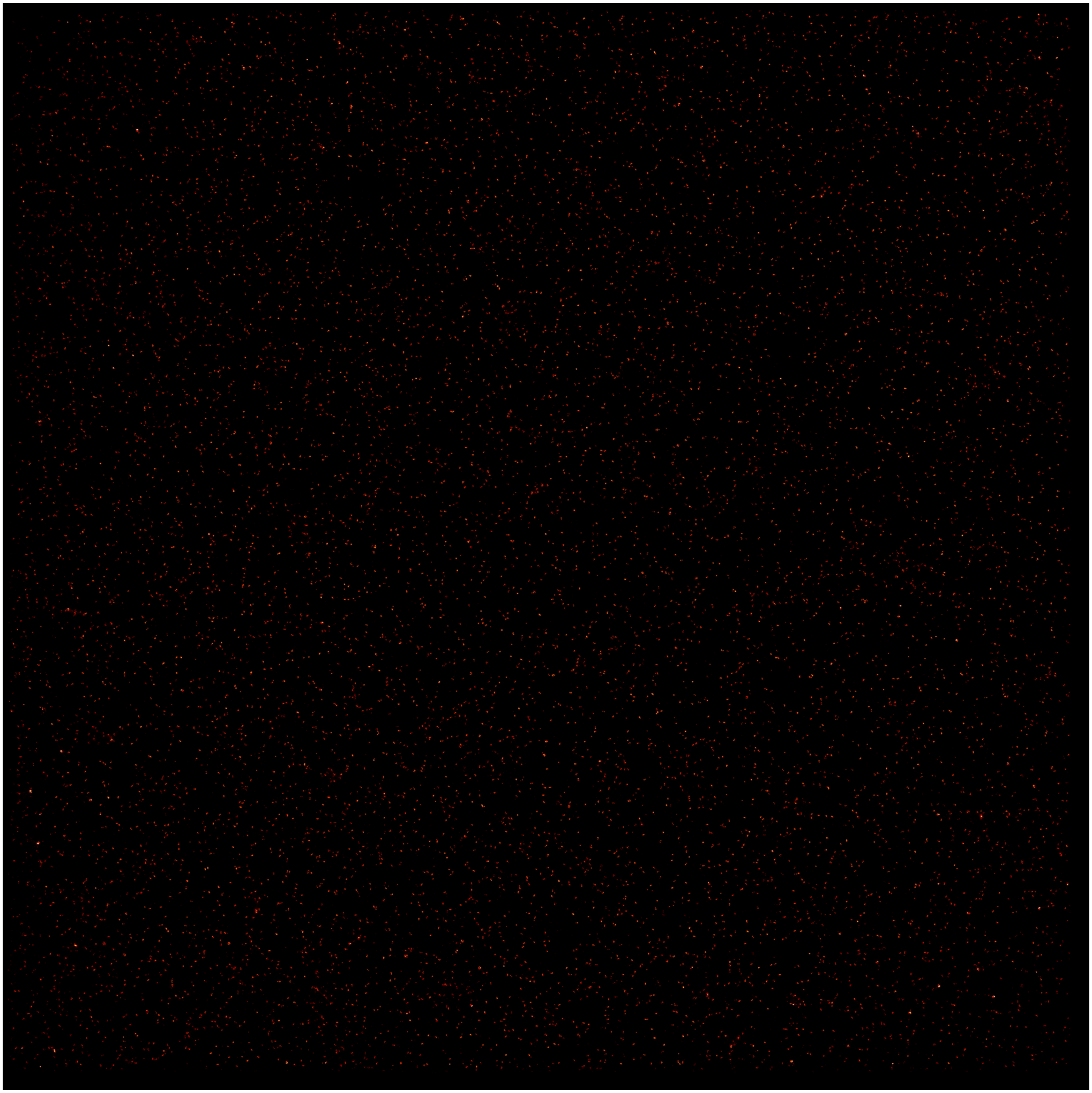

Supplementary Figure 38. Overview image of crosstalk experiment for imager sequence P38. Image size 40.96 μm.

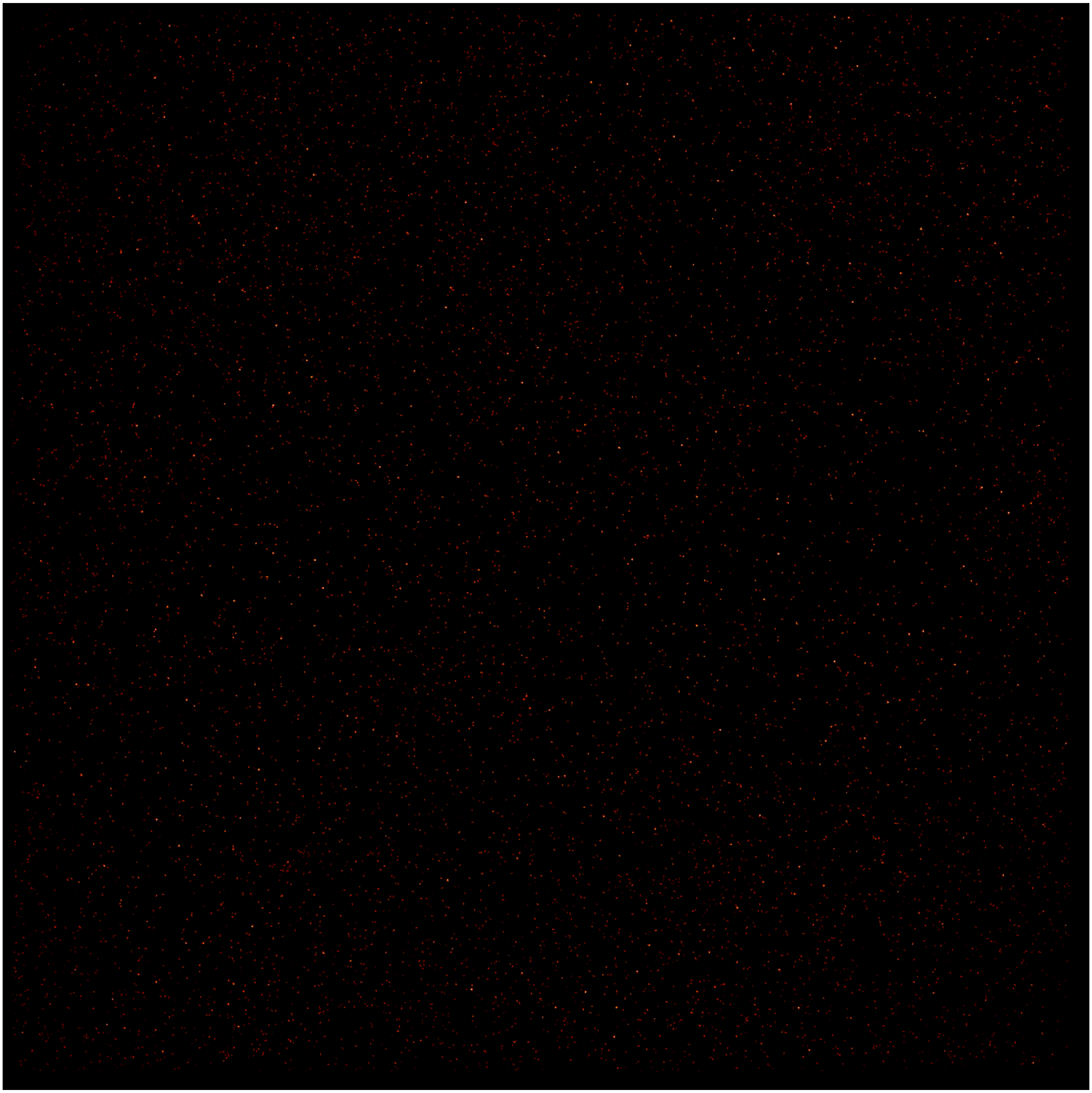

Supplementary Figure 39. Overview image of crosstalk experiment for imager sequence P39. Image size 40.96 µm.

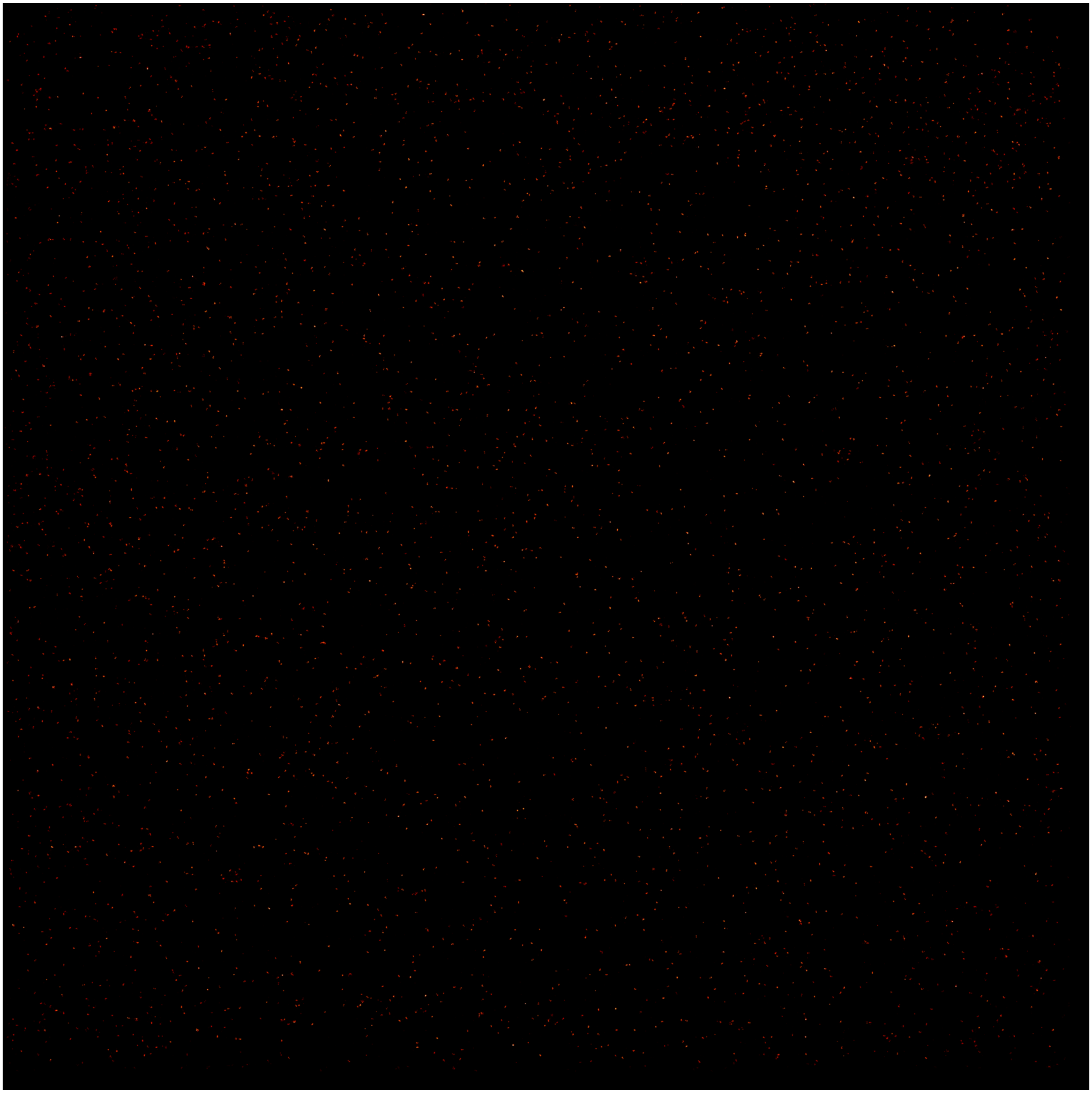

Supplementary Figure 40. Overview image of crosstalk experiment for imager sequence P40. Image size 40.96 µm.

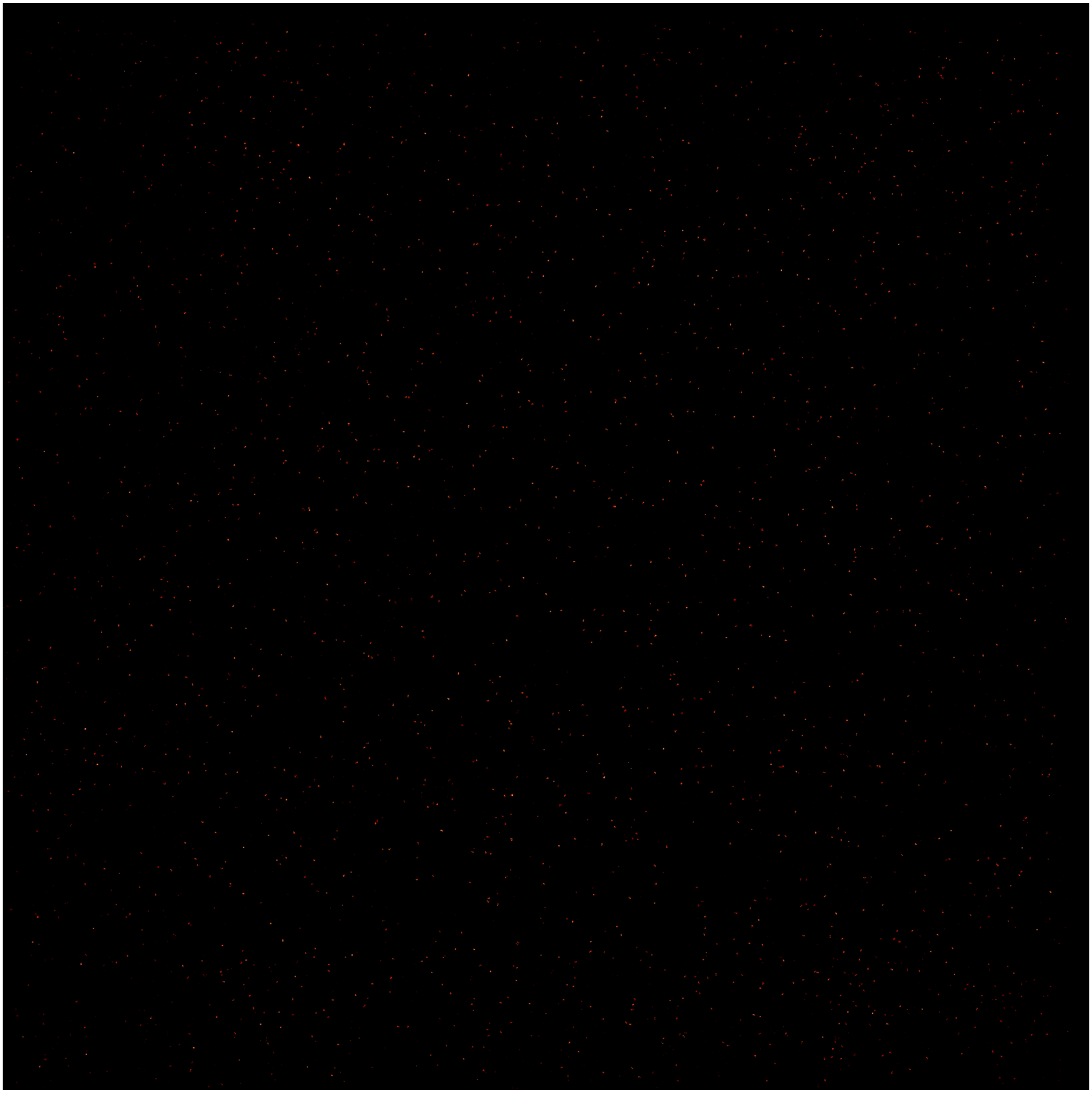

Supplementary Figure 41. Overview image of crosstalk experiment for imager sequence P41. Image size 40.96 µm.

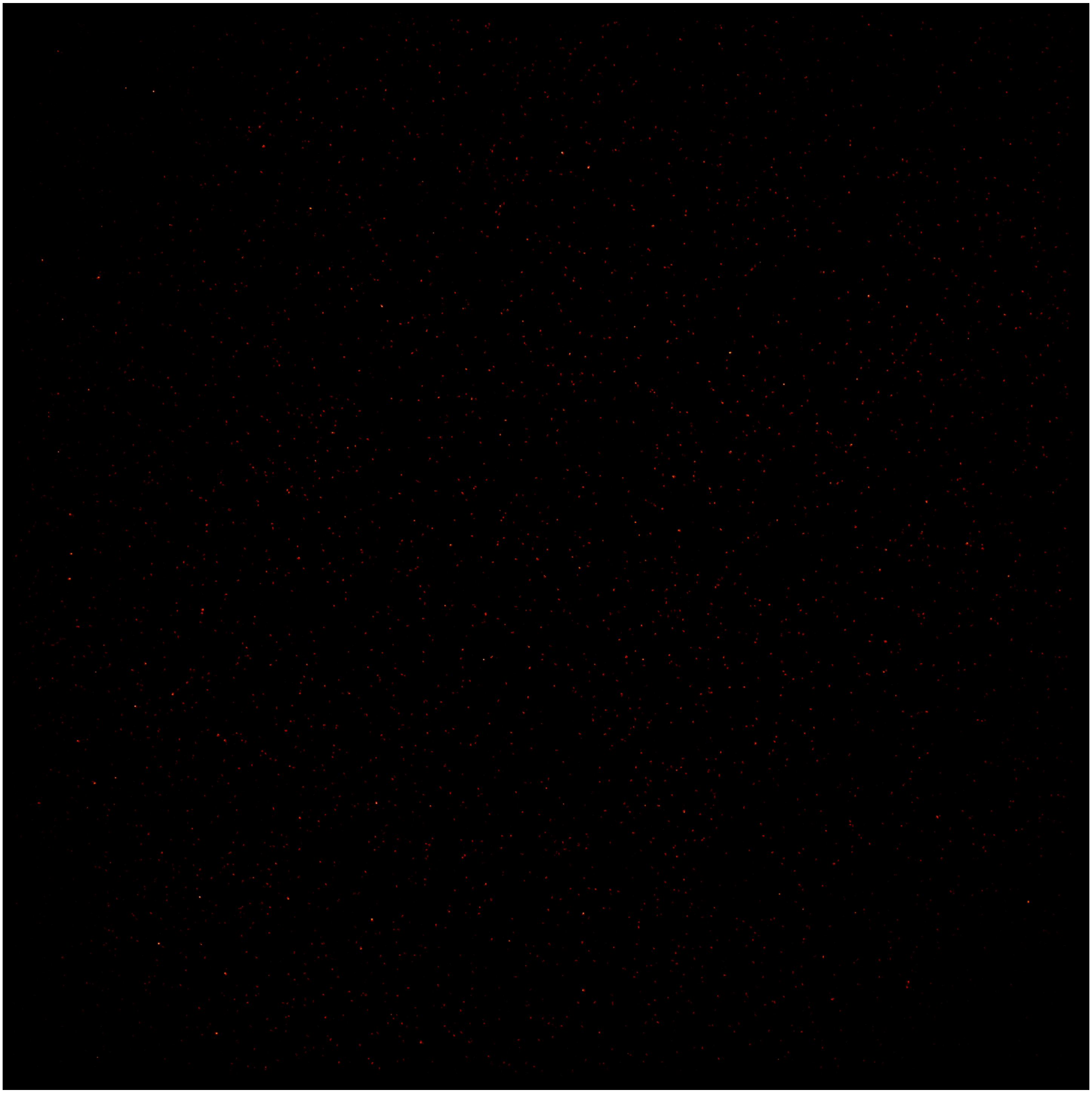

Supplementary Figure 42. Overview image of crosstalk experiment for imager sequence P42. Image size 40.96 µm.

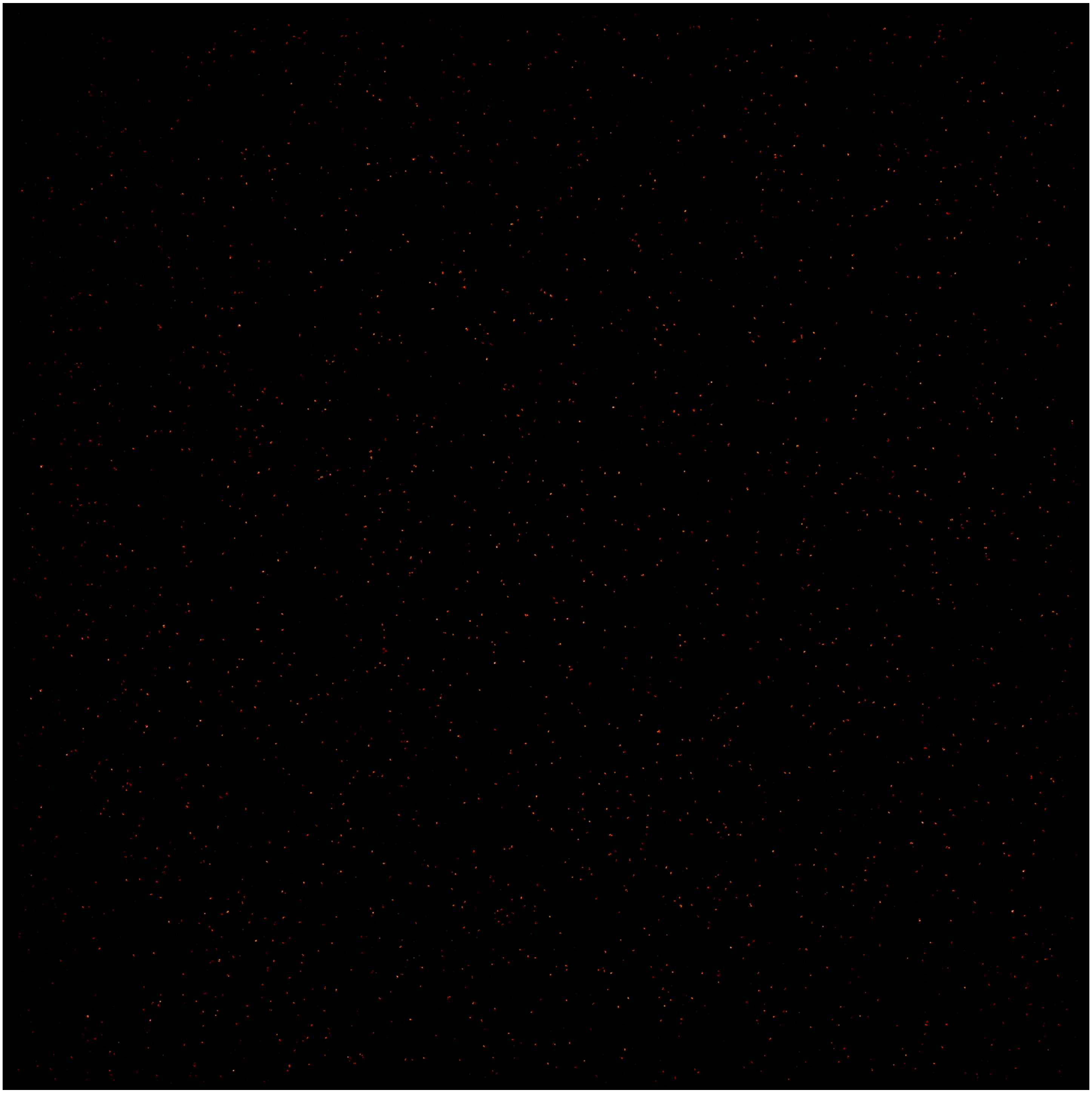

Supplementary Figure 43. Overview image of crosstalk experiment for imager sequence P43. Image size 40.96 µm.

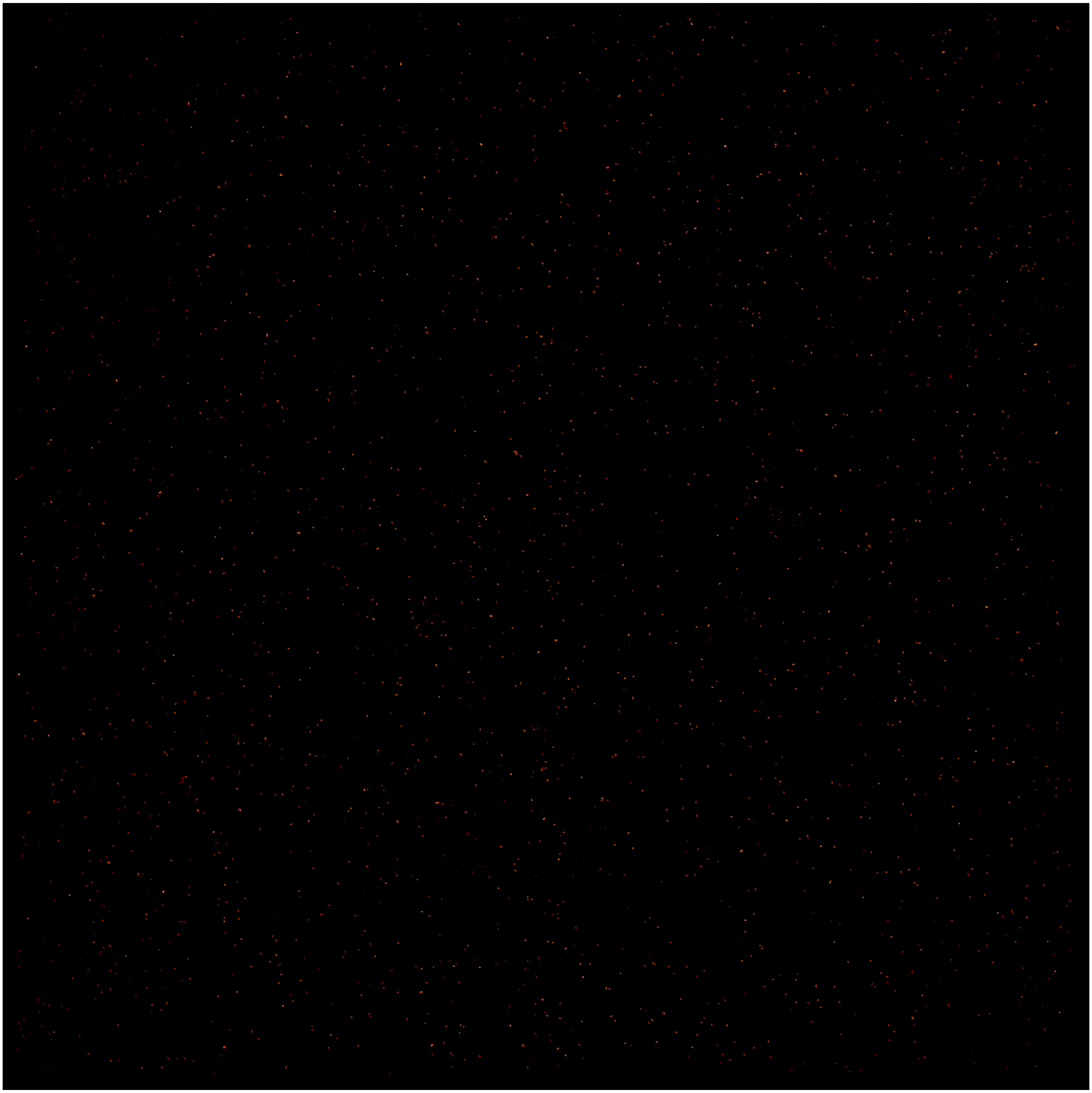

Supplementary Figure 44. Overview image of crosstalk experiment for imager sequence P44. Image size 40.96 µm.

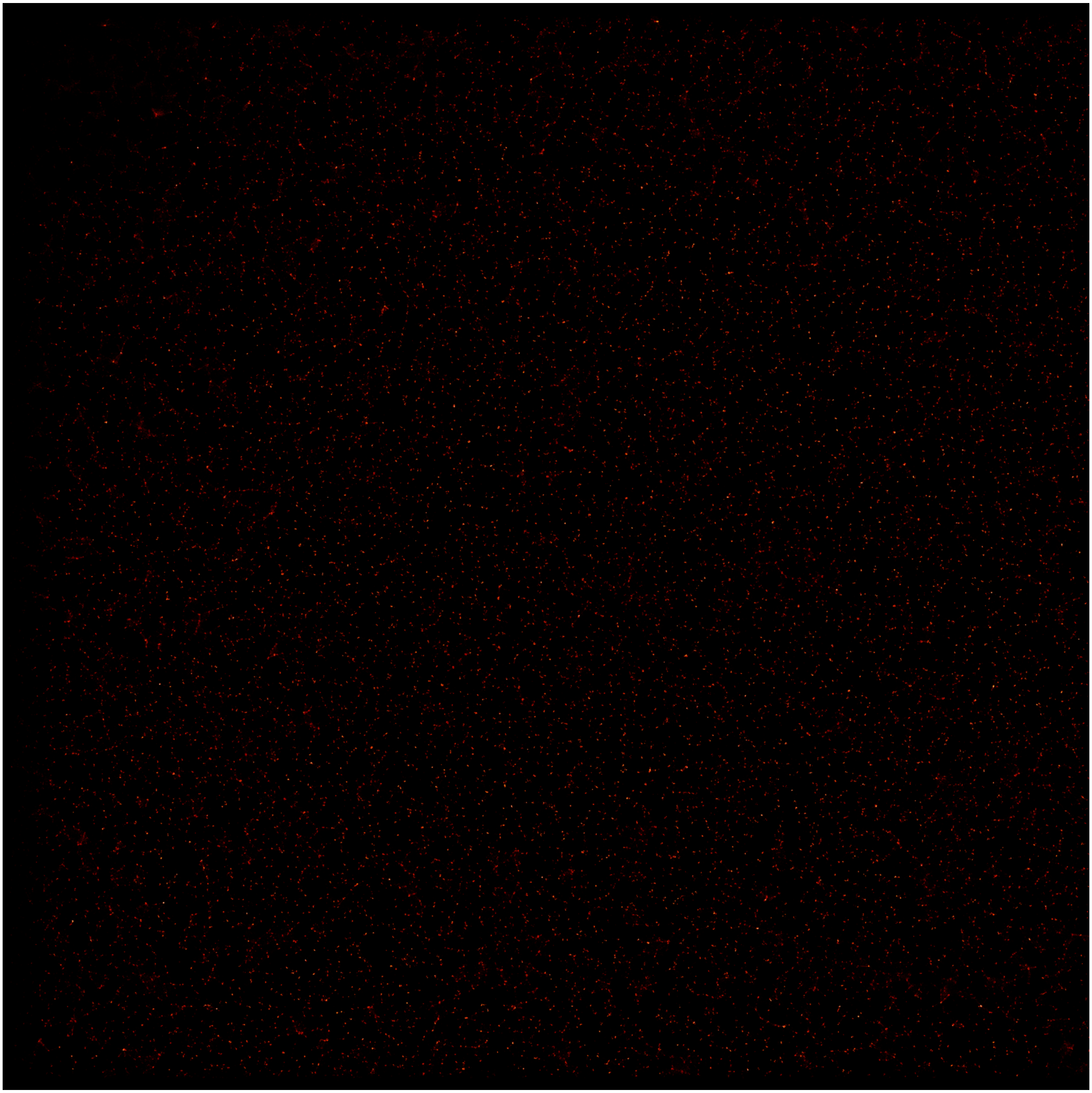

Supplementary Figure 45. Overview image of crosstalk experiment for imager sequence P45. Image size 40.96 µm.

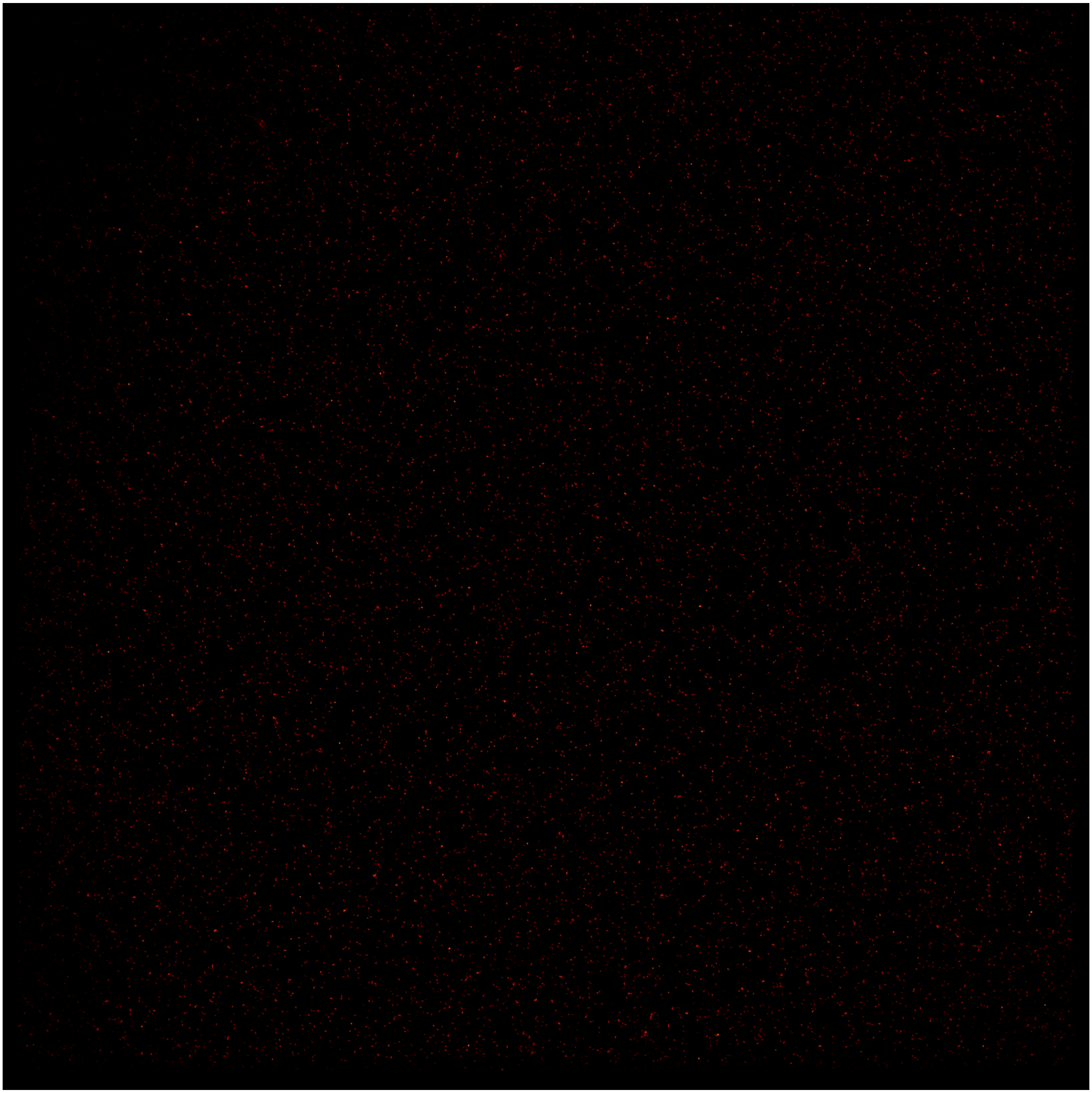

Supplementary Figure 46. Overview image of crosstalk experiment for imager sequence P46. Image size 40.96 µm.

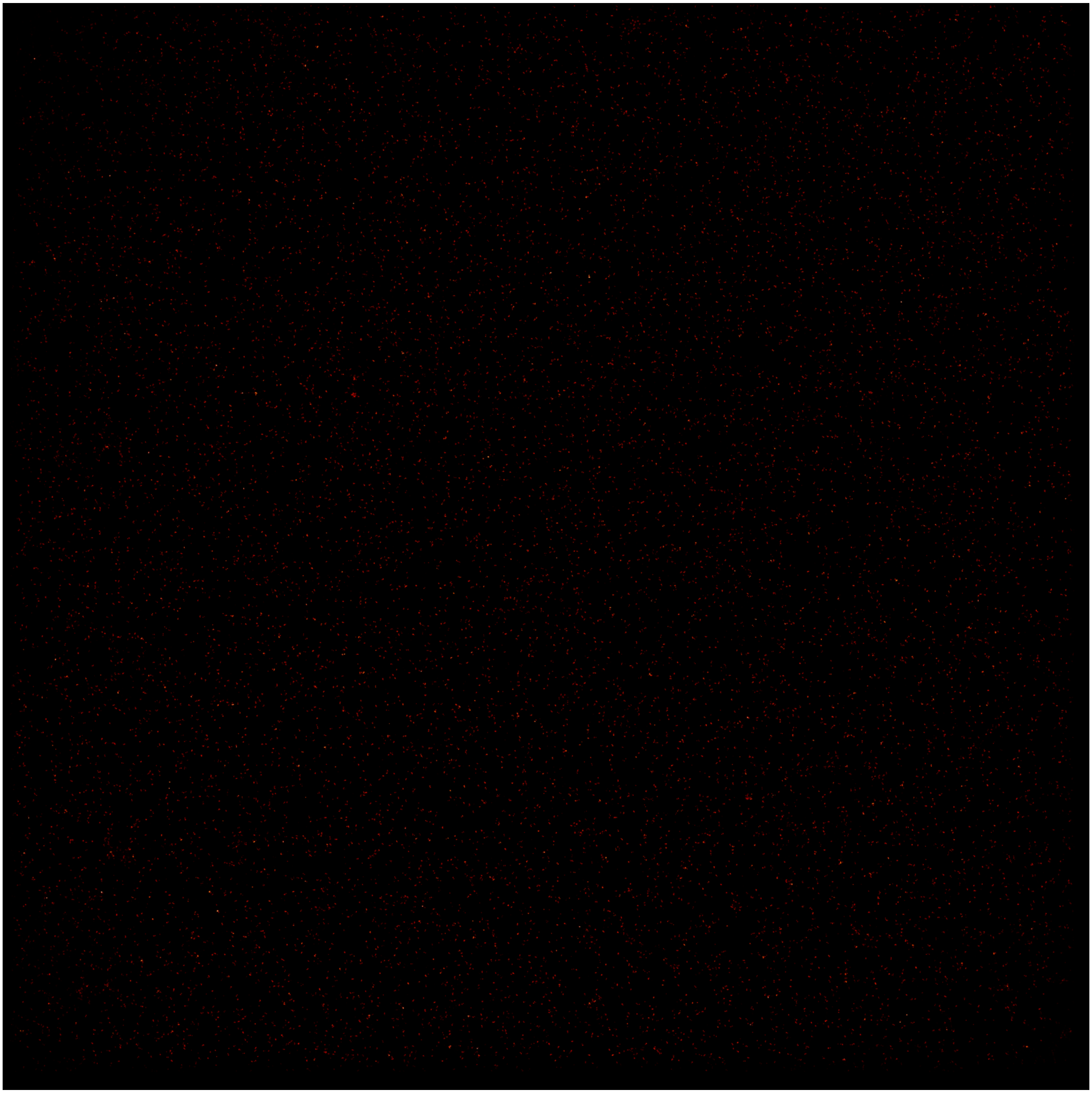

Supplementary Figure 47. Overview image of crosstalk experiment for imager sequence P47. Image size 40.96 µm.

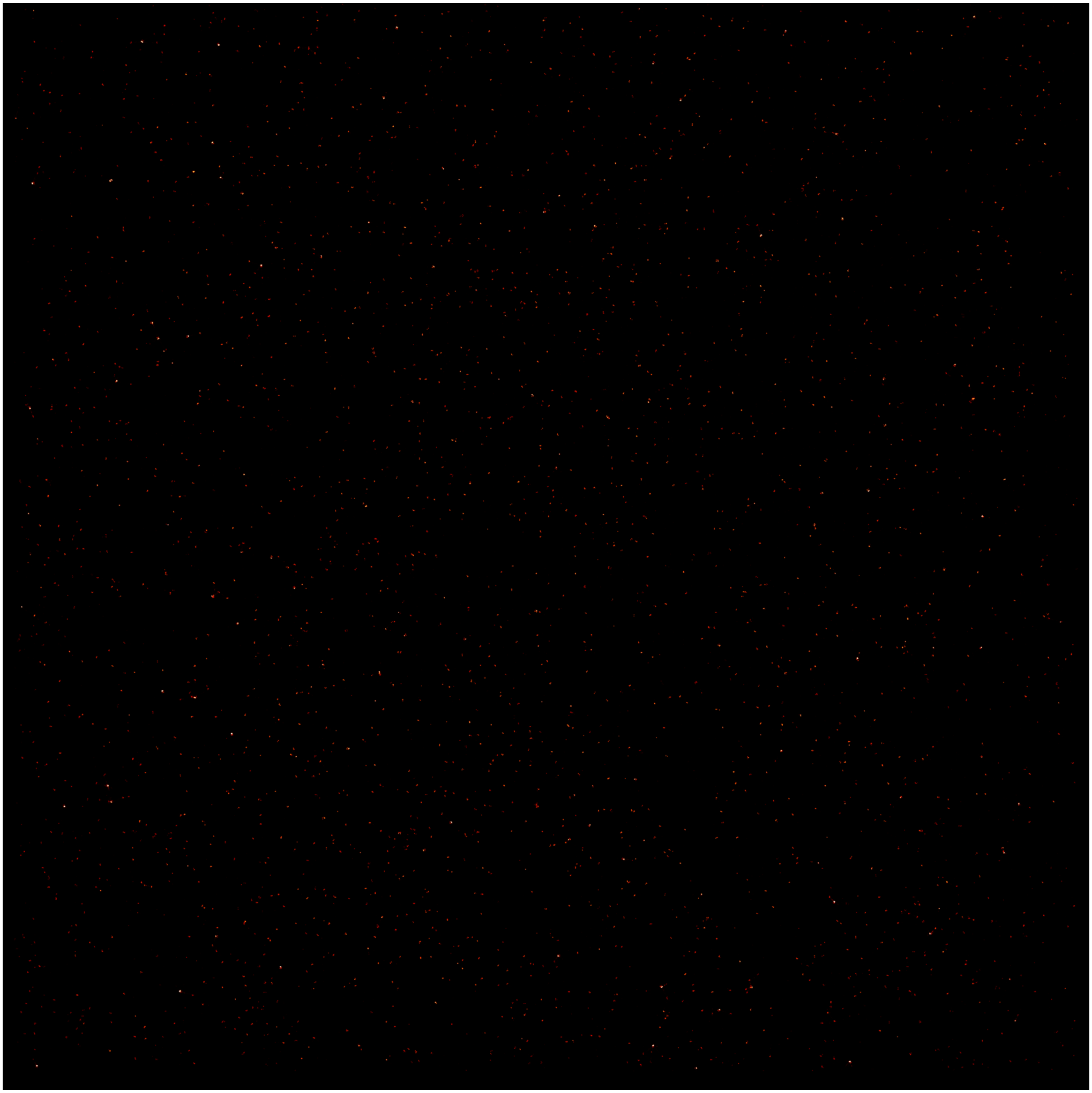

Supplementary Figure 48. Overview image of crosstalk experiment for imager sequence P48. Image size 40.96 µm.

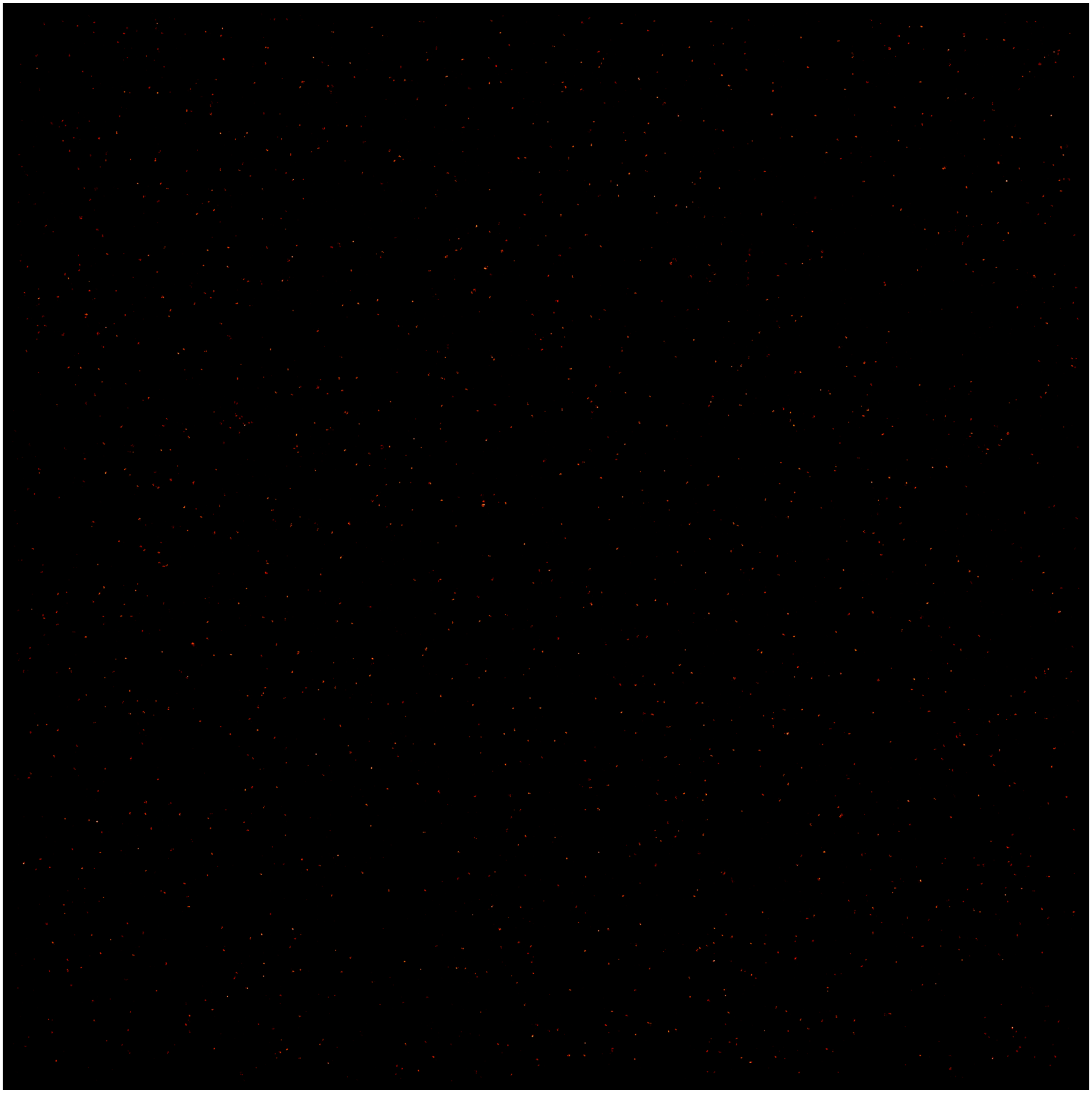

Supplementary Figure 49. Overview image of crosstalk experiment for imager sequence P49. Image size 40.96 µm.

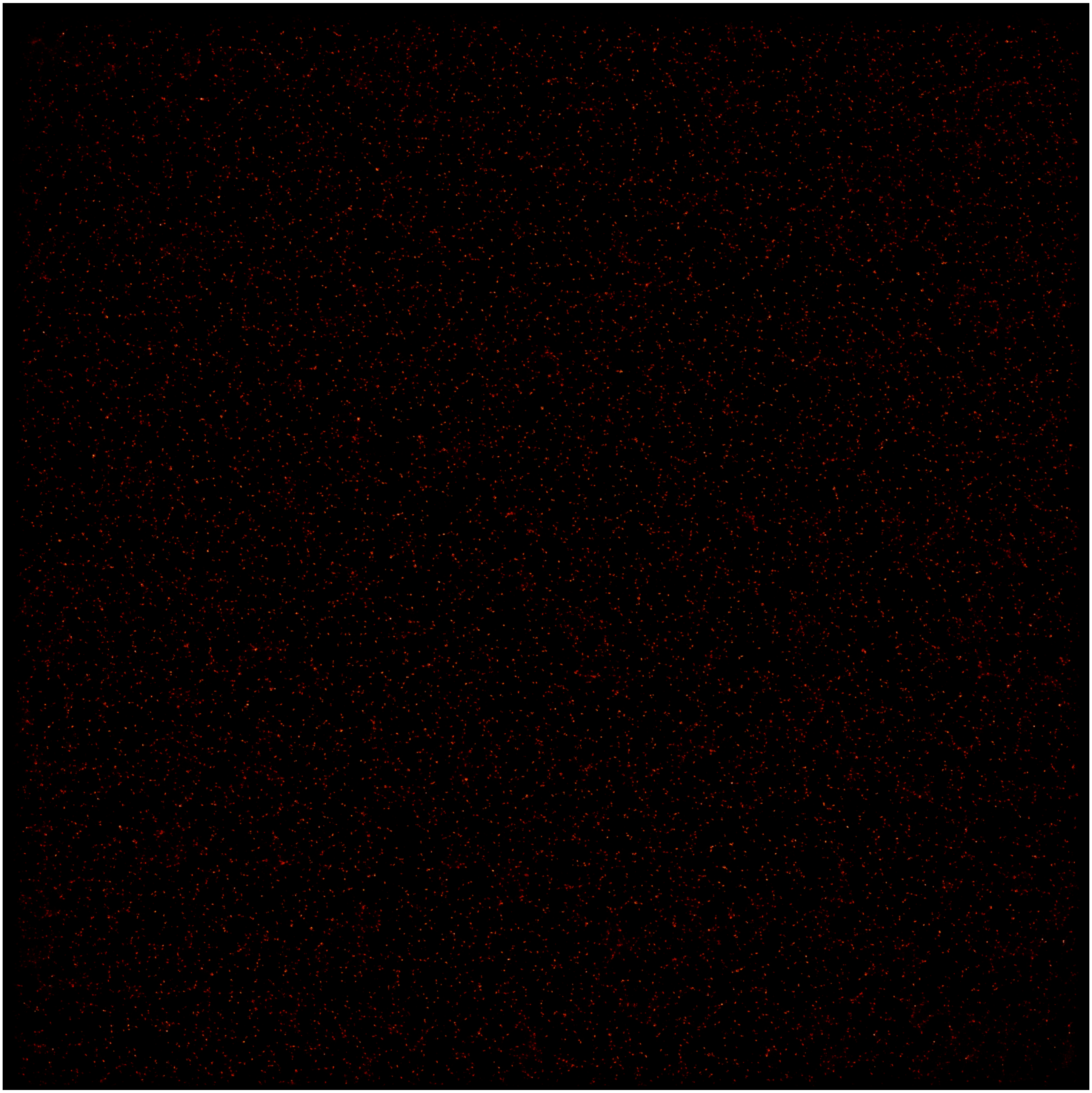

Supplementary Figure 50. Overview image of crosstalk experiment for imager sequence P50. Image size 40.96 µm.

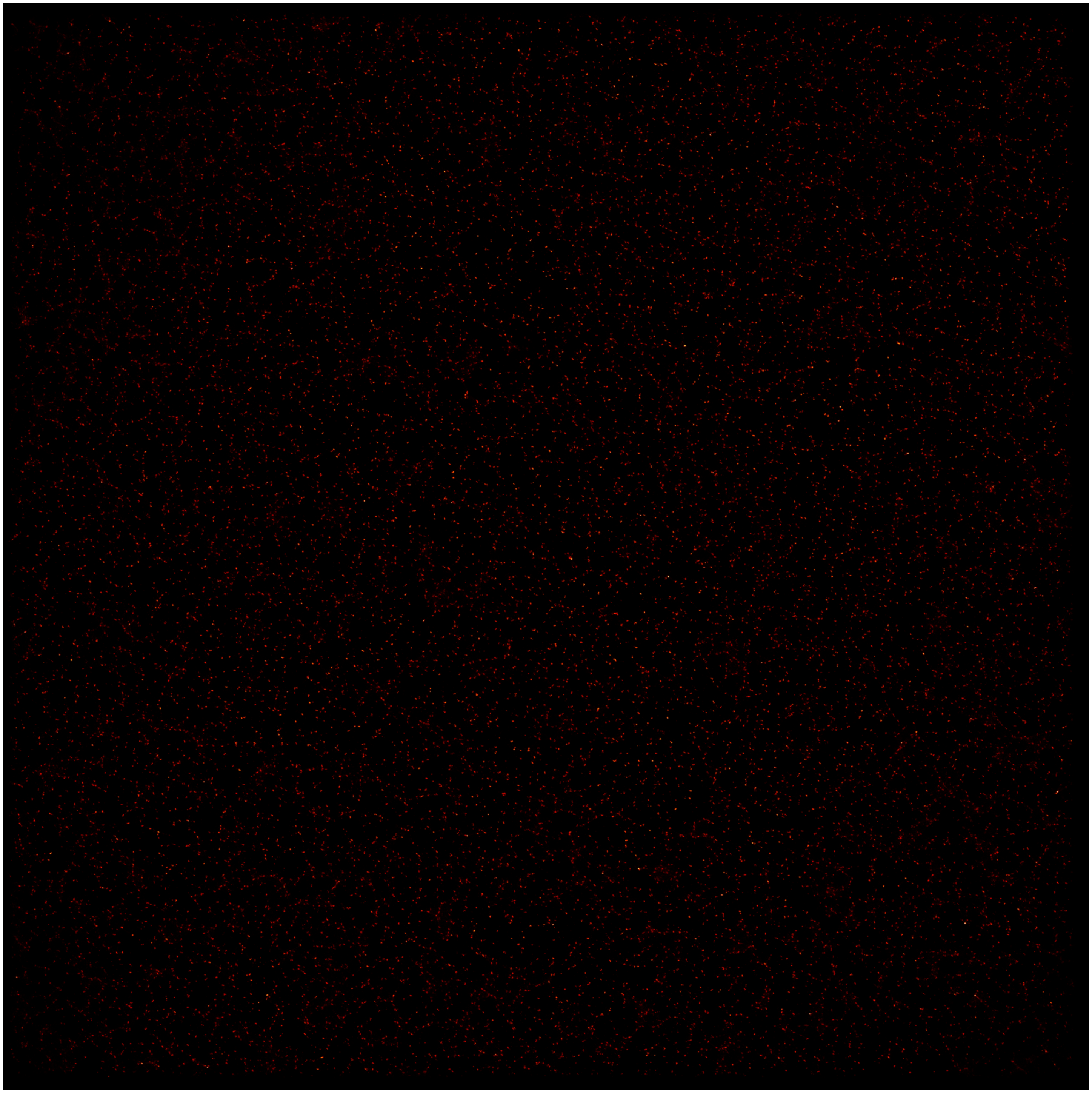

Supplementary Figure 51. Overview image of crosstalk experiment for imager sequence P51. Image size 40.96 μm.

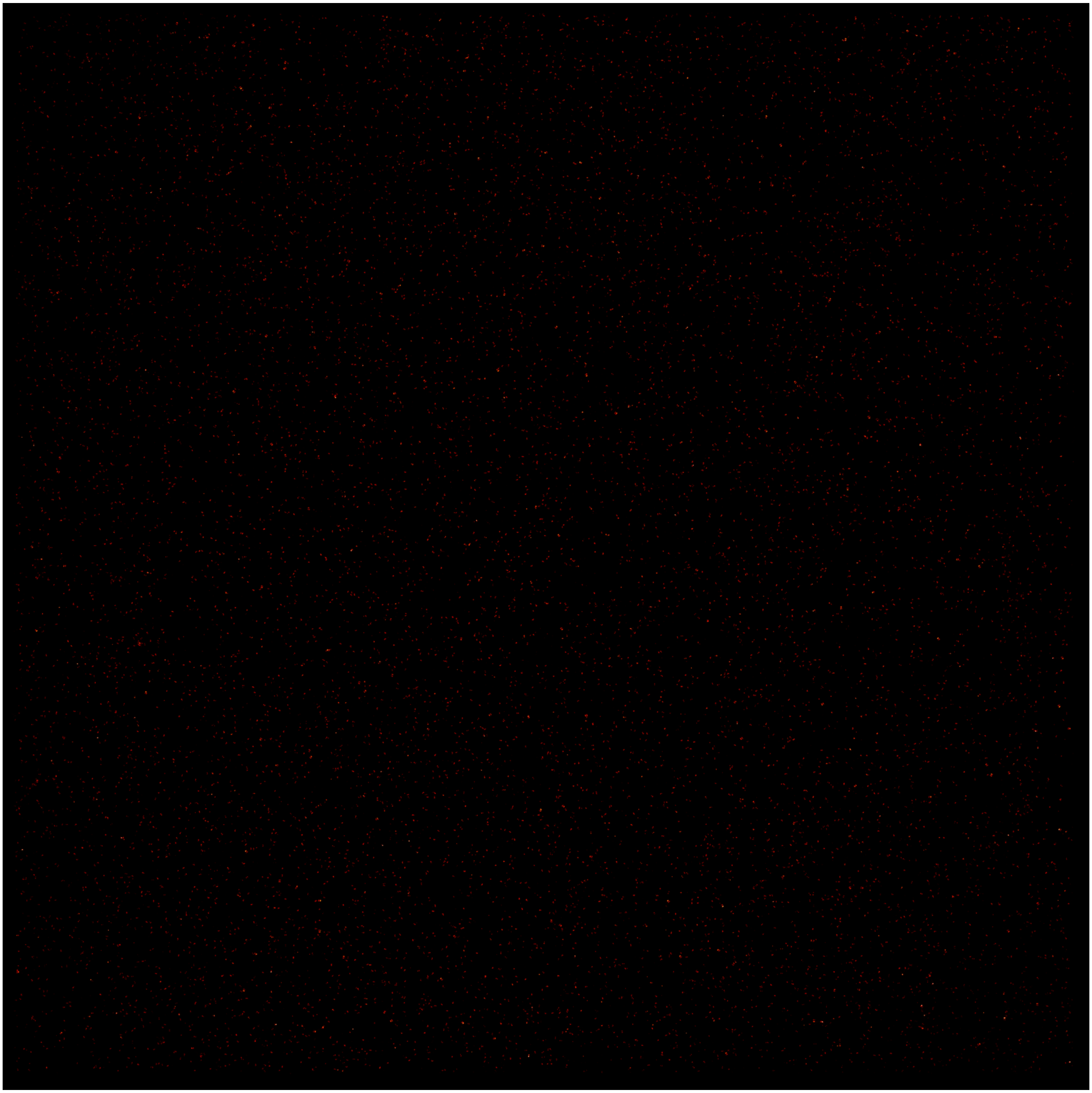

Supplementary Figure 52. Overview image of crosstalk experiment for imager sequence P52. Image size 40.96 µm.

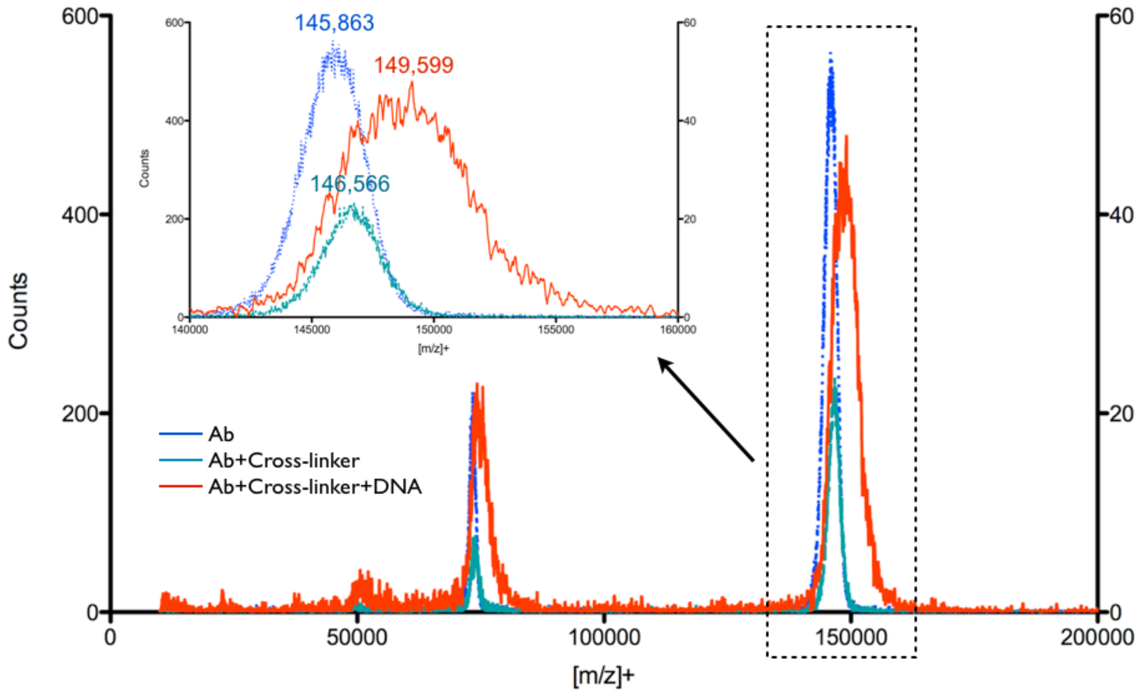

Supplementary Figure 53. Characterization of DNA-Antibody conjugates using MALDI-TOF mass spectrometry analysis. MALDI-TOF mass spectrometry data shows the increase of molecular mass following cross-linker conjugation and subsequent DNA attachment. The difference in mass between DNA-modified and unmodified antibody was used to calculate the number of DNA strands loaded onto a single antibody. Mass of unmodified Ab (Ab^m)=145863 and the mass of DNA modified Antibody (DNA-Ab^m)=149599. Mass difference (DNA-Ab^m-Ab^m) = 3736. Considering the mass of the DNA fragment of ~3306, the number of DNA per antibody was estimated ~1.

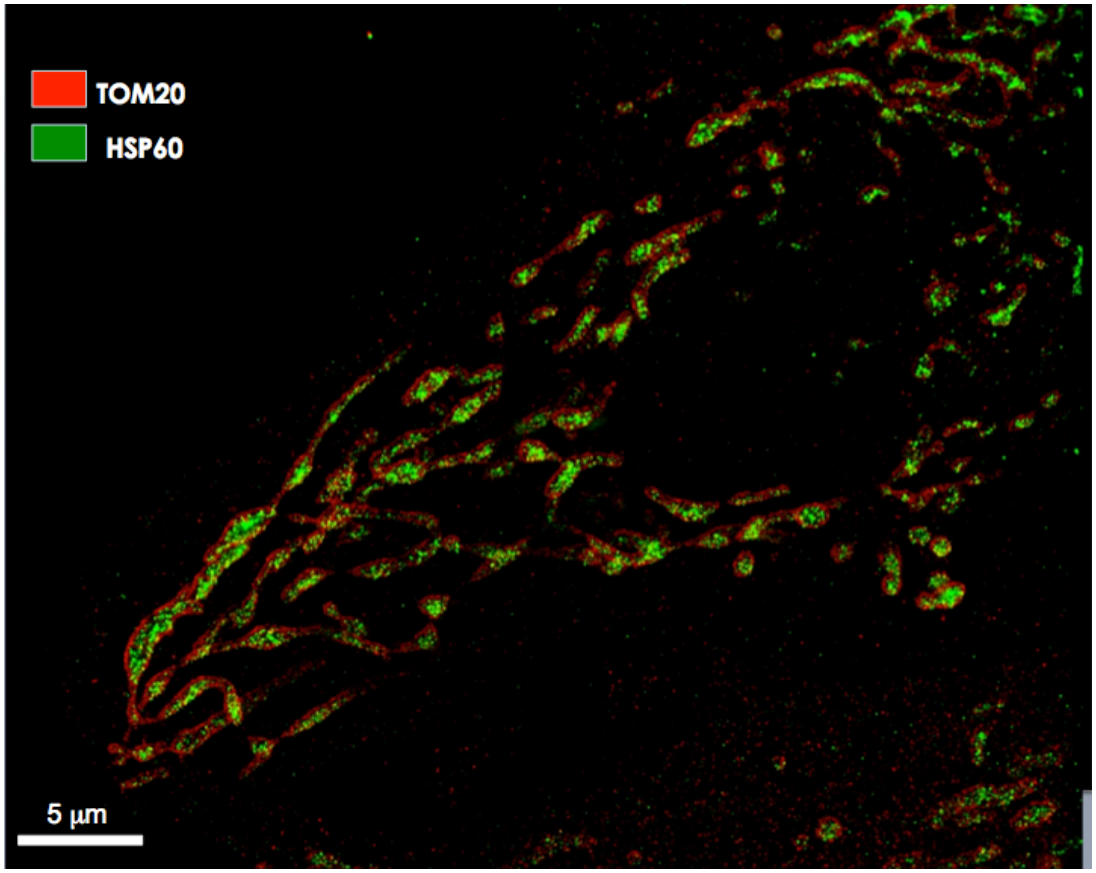

Supplementary Figure 54. Demonstration of spectral dual-color super-resolution imaging using DNA-conjugated secondary antibodies. We co-stained Tom20, a mitochondrial outer membrane protein, and HSP60, a mitochondrial matrix protein in fixed HeLa cells. The images were taken using ATTO655- and Cy3B-conjugated imager strands for Tom20 and HSP60, respectively, without buffer exchange. It can be seen that the HSP60 signal resides "inside" the Tom20 signal, consistent with their actual biological positions.

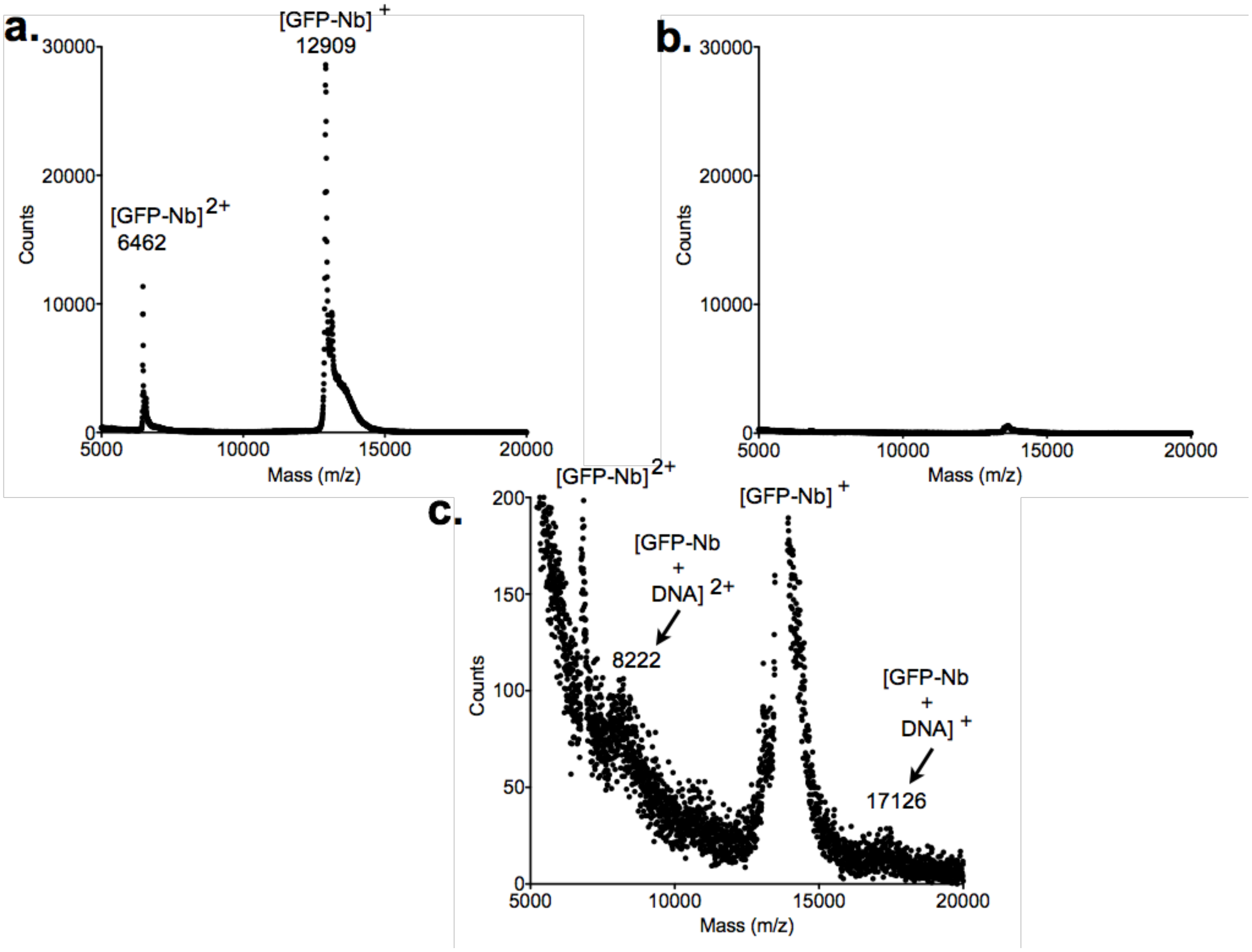

Supplementary Figure 55. Characterization of DNA-Nanobody conjugates using MALDI-TOF mass spectrometry analysis. Although we achieved proper MALDI-MS spectra with high signal to noise for unconjugated nanobody (**a**), the ionization efficiency decreased after conjugation with DNA (**b**). This observation indicates that the successful conjugation has been achieved but makes an accurate determination of the DNA vs. nanobody ratio more challenging as compared to the antibody case. However, upon magnifying the MALDI-MS spectra of the nanobody-DNA conjugate, we do observe two additional peaks (**c**). Upon calculating the molecular weight, we assigned these peaks as [nanobody+1 DNA]$^{2+}$ ion and [nanobody+1 DNA]$^{+}$ ion. This indicates the high probability of achieving single DNA attached nanobody from the conjugation method. The presence of unconjugated nanobody is also observed in the nanobody-DNA conjugate spectra. In this respect, we note that the presence of a trace amount of unconjugated nanobody can dominate the spectra due to its high ionization efficiency. Mass calculation was done using the following values: Mass of the nanobody: ~12909 Da, mass of the DNA strand: ~3469 Da, mass added due to Tz-TCO component: ~ 438 Da.

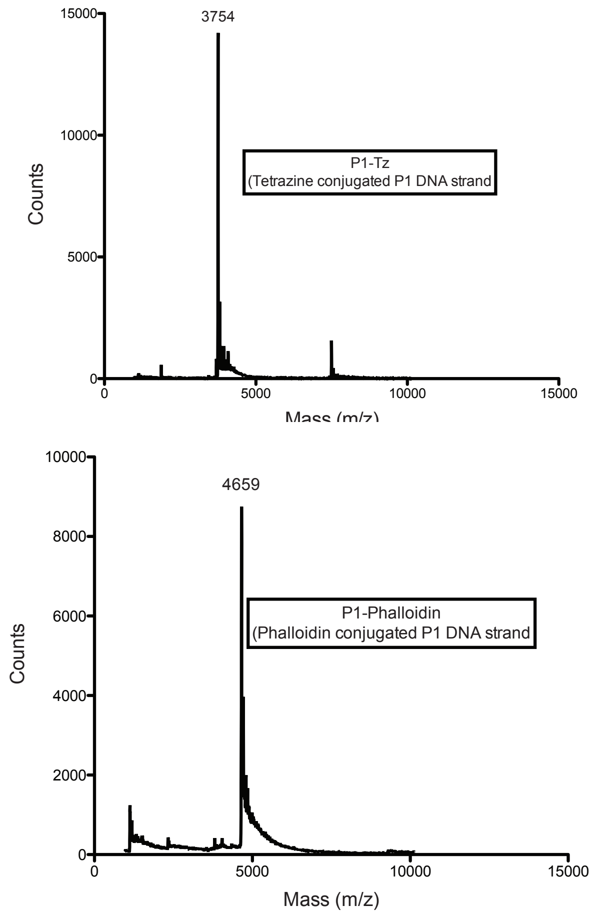

Supplementary Figure 56. Characterization of DNA-Phalloidin conjugates using MALDI-TOF mass spectrometry analysis
